# Lokiarchaea are close relatives of Euryarchaeota, not bridging the gap between prokaryotes and eukaryotes

**DOI:** 10.1371/journal.pgen.1006810

**Published:** 2017-06-12

**Authors:** Violette Da Cunha, Morgan Gaia, Daniele Gadelle, Arshan Nasir, Patrick Forterre

**Affiliations:** 1 Institut Pasteur, Unité de Biologie Moléculaire du Gène chez les Extrêmophiles (BMGE), Département de Microbiologie Paris, France; 2 Institute for Integrative Biology of the Cell (I2BC), CEA, CNRS, Univ. Paris‐Sud, Université Paris-Saclay, Gif-sur-Yvette cedex, France; 3 Department of Biosciences, COMSATS Institute of Information Technology, Islamabad, Pakistan; Vanderbilt University, UNITED STATES

## Abstract

The eocyte hypothesis, in which Eukarya emerged from within Archaea, has been boosted by the description of a new candidate archaeal phylum, “Lokiarchaeota”, from metagenomic data. Eukarya branch within Lokiarchaeota in a tree reconstructed from the concatenation of 36 universal proteins. However, individual phylogenies revealed that lokiarchaeal proteins sequences have different evolutionary histories. The individual markers phylogenies revealed at least two subsets of proteins, either supporting the Woese or the Eocyte tree of life. Strikingly, removal of a single protein, the elongation factor EF2, is sufficient to break the Eukaryotes-Lokiarchaea affiliation. Our analysis suggests that the three lokiarchaeal EF2 proteins have a chimeric organization that could be due to contamination and/or homologous recombination with patches of eukaryotic sequences. A robust phylogenetic analysis of RNA polymerases with a new dataset indicates that Lokiarchaeota and related phyla of the Asgard superphylum are sister group to Euryarchaeota, not to Eukarya, and supports the monophyly of Archaea with their rooting in the branch leading to Thaumarchaeota.

## Author summary

Two scenarios have been proposed to describe the history of cellular life on our planet. For some authors, two lineages emerged from the last universal cellular ancestor, one leading to Bacteria, the other one leading to a common ancestor of Archaea and Eukarya (Woese’s hypothesis), while others suggest that Eukaryotes emerged from within an archaeal subgroup (eocyte hypothesis). This latter hypothesis has been boosted by the reconstruction of new archaeal genomes from environmental DNA. These analyses have suggested that eukaryotes originated from complex archaea, called Lokiarchaeota, the first described members of the recently proposed Asgard superphylum. Considering the importance of this question, we performed new analyses of the universal proteins from Lokiarchaea and realized that their affiliation to Eukaryotes was most probably due to different biases, including chimeric sequences and unequal rate of protein evolution. From our results, we suggest here that Lokiarchaea and close relatives are sister group to Euryarchaeota, not to Eukarya. Notably, we also show that the choices of the universal markers to include in one’s analysis will critically impact the scenario supported and that some markers as the RNA polymerase support the traditional Woese’s tree.

## Introduction

The topology of the tree of Life (ToL), especially the evolutionary relationships between Archaea and Eukarya, is a major debated question in Biology that deeply impacts our understanding of the history of life on Earth [1–6]. Two main hypotheses are opposed: in the first, Archaea and Eukarya are sister groups sharing a common ancestor [7], whereas in the other, Eukarya emerge from within Archaea, as sister group to an archaeal subdivision (eocytes *sensu* Lake, 1984 [8]). In the first hypothesis, specific eukaryotic features such as spliceosomes, mitosis and meiosis, or else the nucleus and the nucleolus, could have originated at different periods in the history of life, some of them being already present in the last common ancestor of Archaea and Eukarya [4,9,10]. In contrast, the emergence of these features is more constrained in the eocyte hypothesis, since all specific eukaryotic features should have necessarily evolved rather recently in a particular ancestral archaeal lineage [3,6,11–13].

The eocyte hypothesis has been boosted two years ago by the publication of new archaeal genomes [14] that were attributed to organisms corresponding to a group of uncultivated archaea called the Deep Sea Archaeal Group (DSAG)[15,16]. The correlation between DSAG abundance and geochemical parameters, as well as FISH analyses, have suggested that DSAG are anaerobic or microaerobic archaea (small 0.2–0.4 μm coccoid-shaped cells) possibly involved in the cycling of iron and/or manganese compounds [17]. Spang and co-workers sequenced a metagenomic sample enriched in DSAG 16S rRNA from sediments recovered from the bottom of the Arctic Mid-Ocean Ridge near a hydrothermal site called the “Loki’s Castle”. Using *in silico* approaches, they reconstructed two partial genomes of DSAG organisms (renamed Lokiarchaea) (Loki 2 and 3) and one nearly complete genome (Loki 1)[14]. In the phylogenetic tree reconstructed from the concatenated alignment of 36 universal proteins, the three Lokiarchaea branched between Euryarchaeota and the putative ‘TACK’ superphylum, which groups Thaumarchaeota and Crenarchaeota with the candidate phyla Aigarchaeota and Korarchaeota [18]. Moreover, eukaryotes emerged within Lokiarchaeota, being sister group to Loki 3 (Fig 2b in [14]). In a strict cladistic view, this position implies that Eukarya are themselves a subdivision of the phylum Lokiarchaeota and extends the proposed TACK superphylum to TACKL (i.e. inclusion of Lokiarchaeota). Remarkably, the genomes of the three Lokiarchaea encode many eukaryote-specific proteins (ESPs) never before detected in Archaea, such as multiple G-proteins and novel components of the ESCRT-III vesicular transport system, supporting the idea that Eukarya originated from an ancestral Lokiarchaeon [14]. This result has been widely reported with Lokiarchaeota being presented as the “missing link” that bridges the gap between prokaryotes (simple life) and eukaryotes (complex life) and as an almost definitive argument supporting the eocyte hypothesis [6,13,19]. Indeed, several recent studies have already mined the Lokiarchaeota genomes to reconstruct the critical pathways of eukaryogenesis [20–24]. The proposed Lokiarchaeota-Eukarya affiliation was only challenged by Caetano-Anolles and colleagues who noticed that the lokiarchaeal proteomes added only 10 new members (0.1%) to the archaeal protein fold superfamilies [25]. More recently, these authors showed that the imbalanced number of species in the dataset studied by Spang *et al*. (10 Bacteria, 10 Eukarya and 87 archaea) could have impacted the topology of the Tree [26].

To position the Lokiarchaota within the Tree of Life, Spang and co-workers concatenated 36 universal markers [14]. They reported that most individual protein trees were not resolved, and suggested that it was due to the small amount of information contained in single-gene alignments [14]. Besides a lack of phylogenetic signal, we think that another possible explanation could be the inclusion of many sequences from taxa known to be fast-evolving in their dataset. In their main phylogenetic tree (thereafter called the *Loki ancestor tree*, Fig 2b in [14]), Archaea were rooted in the branch leading to *Methanopyrus kandleri*, a notorious fast-evolving archaeon that normally branches close to *Methanobacteriales* in the Euryarchaeota phylum [27]. It is well known that inclusion of fast-evolving species (FES) in datasets can lead to long-branch attraction (LBA) artefacts [28,29]. The presence of LBA that could impact Eukarya-Lokiarchaota association in the Loki ancestor tree is suggested by the tests performed by Spang *et al*. in which they selectively removed individual archaeal phyla (Supp Fig S13 in [14]). This analysis showed that the removal of slow-evolving and known phyla strengthen the Lokiarchaea-Eukarya association, whereas the removal of FES weaken it.

Hervé Philippe and co-workers recently confirmed that even recent Bayesian methods of tree reconstruction cannot eliminate LBA when the outgroup is very distant [9], which is precisely the case when Bacteria are used as outgroup to determine the relationships between Archaea and Eukarya. Another possible pitfall could be sequence contamination since the DSAG-enriched sample used for *in silico* reconstruction of the lokiarchaeal genomes also contained sequences from Bacteria and Archaea such as Thaumarchaeota, the DSAGs representing 10% of the microbial diversity observed by 16S rRNA sequencing [14]. The possibility of contamination cannot be easily dismissed because the authors did not reconstruct the lokiarchaeal genomes from DNA obtained from single-cells but from environmental DNA. In fact, the Loki 1 genome size was rather large (5.1 Mb and estimated to be 92% complete) for possible microaerobic archaea [17].

In this study, we reanalyzed the individual phylogenies of the 36 universal proteins used in the concatenated analysis of Spang and co-workers to reassess the robustness of the phylogenetic position of Lokiarchaea and its affiliation with Eukarya. We also investigated the position of the archaeal phylum ‘Thorachaeota’, shown to be sister group to Lokiarchaeota in a phylogenetic tree based on the concatenated alignment of 16 ribosomal proteins [30], and of more recently described related phyla (forming altogether the putative Asgard superphylum [31]). Removal of FES from the initial dataset revealed different stories for the lokiarchaeal proteins, as well as for different universal proteins. We identified a subset of proteins (hereafter called the Woese’s proteins) that support the three-domains ToL [7] in which Archaea are monophyletic, and another subset of proteins (hereafter called the eocyte proteins) that support trees in which Eukarya are sister group to various archaeal lineages (hereafter called the eocyte trees). Remarkably, exclusion of a single protein, Loki 3 Elongation Factor 2 (EF2; likely contaminated by eukaryotic sequences) was sufficient to break the Lokiarchaeota/Eukarya affiliation with the Spang *et al*. dataset, and to recover the Woesian ToL with the FES-curated dataset. Finally, we performed a robust phylogenetic analysis of the two largest RNA polymerase subunits using a new dataset containing an equal number of species from each domain, Archaea, Bacteria and Eukarya. Our results support the monophyly of Archaea and suggest that Lokiarchaeota and related phyla are sister group to Euryarchaeota and not to Eukaryotes. The RNA polymerase phylogeny branches the archaeal domain in Thaumarchaeota, suggesting that the TACK superphylum [18] might not be a valid phylogenetic unit.

## Results

### The universal lokiarchaeal proteins have different origins

We performed individual phylogenetic analyses of the 36 universal proteins used by Spang and co-workers, using the same methodology (Maximum likelihood, ML). The 36 phylogenetic trees obtained are presented in S1 Fig. We noticed, as stated by the authors, that most individual phylogenies provided trees without support at most nodes (summarized values for this initial dataset in Table 1, detailed values in S1 Table). Notably, the monophyly of at least one major archaeal phylum (Euryarchaeota, Crenarchaea or Thaumarchaeota) was never recovered with significant support, with very few exceptions (Table 1); one protein, the ribosomal protein L1, supported the Woese’s tree with 100% bootstrap (BS) value, whereas all the others supported an eocyte tree (S1 Table). However, Eukarya emerged from Archaea at very different positions in the 35 eocyte trees. Specifically, Eukarya were sister group to one or two Lokiarchaea in 9 trees, but always without statistical support (BS<70%) except in the case of SecY (BS value of 84% with Loki 1/3), and sister group to the three Lokiarchaeal proteins, as in the lokiarchaeal ancestor tree, only in the case of EF2 with strong support (BS values of 100% with Loki 3).

**Table 1:** Comparative analysis of the 36 individual phylogenetic trees obtained with the initial and the curated datasets.

	Initial dataset	Curated dataset
Monophyly of Euryarchaeota	**5**	**17**
BS > 50	0	8
BS > 80	0	3
Monophyly of Crenarchaeota	**14**	**18**
BS > 50	4	10
BS > 80	1	2
Monophyly of Thaumarchaeota	**14**	**22**
BS > 50	5	10
BS > 80	2	2
Monophyly of Archaea (BS = 100)	**1**	**11**
Loki-Eukarya sister group	**10**	**11**
BS > 50	3	5
BS > 80	2 (EF2, SecY)	1 (EF2)

Number of trees displaying the monophyly of Archaea and of the major archaeal phyla, as well as those in which Lokiarchaea and Eukarya are sister groups, with the initial dataset (10 Bacteria, 10 Eukaryotes and 84 Archaea) and the curated dataset (10 Bacteria, 10 Eukaryotes and 61 Archaea). The number of trees are indicated depending on the bootstrap support (BS) values supporting the corresponding nodes.

The positions of the 90 Lokiarchaeal universal proteins used in the concatenation of Spang and co-workers (two or three Loki per protein family) varied considerably in the 36 individual trees (green leaves in S1 Fig). The two or three Loki proteins branched closely together only in 15 of the 36 trees, but at different positions either within Archaea or in four cases as sister group to Eukarya. In the 21 other trees, the Loki proteins branched separately at very different positions, and in 5 cases, one (or two) Loki proteins branched within Archaea, whereas the other(s) branched as sister group to Eukarya.

Notably, in around a half of the phylogenies, Loki proteins branched within or as sister group to environmental archaeal sequences, to sequences of known archaeal fast-evolving species (FES) (as *M*. *kandleri* and *Nanoarchaeum equitans* [32–34]), or to sole representative of their lineage (as “*Candidatus* Korarchaeum cryptofilum” [35]). Previous analyses have clearly shown that *M*. *kandleri* and *N*. *equitans* are fast-evolving species whose correct position in the archaeal tree can be only recovered by very careful analyses [32,33]. *M*. *kandleri* turned out to be sister group of Methanobacteriales (they also are the only archaeal species containing pseudomurein) and *N*. *equitans* an early branching Euryarchaeon, possibly sister group of Thermococcales. These positions were not recovered in most of the individual trees obtained here. In contrast, *M*. *kandleri* and *N*. *equitans* were often grouped with environmental sequences and “*Ca*. K. cryptophylum”, and all these sequences were frequently located at the base of the archaeal tree, suggesting an attraction effect induced by the long bacterial branch. To test if the observed variability of lokiarchaeal proteins positions within most of the 36 universal trees could be explained by the presence of all these FES in the original dataset, we decided to reanalyze the data of Spang and co-workers after removing all species known to be fast-evolving (such as *M*. *kandleri*, *N*. *equitans*), or “*Ca*. K. cryptophylum” that was already mentioned as possible source of artefact (Supplementary data in [14]). We also removed all environmental and genomic sequences obtained by metagenomics reconstruction for which the presence of FES cannot be excluded (S1 Fig). Indeed, many of them, such as Parvarchaeota, correspond to nanosized archaea with small genomes and limited metabolic capacity, suggesting that they are evolving by genomic reduction [36]. This interpretation is supported by the fact that many of them branch with Nanoarchaea in archaeal trees and share with them instability regions in universal protein sequences that are not present in other archaea (see methods section for more details).

Inspection of individual ML phylogenies obtained without presumptive and *bona fide* FES (thereafter simply called FES) revealed a clear-cut improvement in trees resolution (summarized values for the curated dataset in Table 1, detailed values in S1 Table, and trees in S2 Fig). The monophyly of at least one major archaeal phyla was now recovered more frequently, especially in trees obtained with large proteins (S1 Table). Furthermore, with few exceptions, the BS values at the monophyletic nodes previously detected with the initial dataset were higher. However, the positions of the different lokiarchaeal proteins remained variable from one tree to another, even when major archaeal phyla were monophyletic, indicating that this odd behavior was not due to FES. We calculated that 71 of the 90 lokiarchaeal proteins branched within Archaea, whereas 19 branched between Archaea and Eukarya or as sister group to Eukarya, suggesting at least two different origins for lokiarchaeal proteins. Loki 1, Loki 2 or Loki 3 proteins were rather equally present in proteins with or without specific eukaryotic affinity, indicating that all three lokiarchaeal genomes included proteins from different sources. The extreme variability in the positions of Lokiarchaeal proteins in individual phylogenies should probably have prevented their use as concatenated markers to determine the position of Lokiarchaeota in the universal tree, since concatenation of protein sequences (i.e. the supermatrix method) can only increase statistical support as long as most of the genes have a congruent evolutionary history [37]. Indeed, sophisticated methods that have been developed these last decades for the phylogenetic analysis of concatenated datasets assume that most proteins have a congruent evolutionary history and hence were designed to deal with the few proteins included that could have been transferred and display a conflictual history. However, this does not seem to be the case here, with at least two opposite evolutionary histories embedded in lokiarchaeal proteins.

### Removing fast-evolving sequences revealed conflicting histories within universal protein markers

The removal of FES from the initial dataset dramatically increased the number of trees in which Archaea were monophyletic (curated dataset in Table 1, detailed in S1 Table). We obtained 11 protein trees in which the monophyly of Archaea was supported with 100% BS, as compared to one in the dataset with FES, and 25 proteins displaying an eocyte tree (35 in the dataset with FES)(S2 Fig, S1 Table). This clearly indicates that the addition of FES generally favors the eocyte *versus* the Woese’s trees in the analysis of universal proteins. Based on this analysis, we divided the 36 markers in two protein subsets: the 11 proteins that support a Woese’s ToL (thereafter called Woese’s proteins), and the 25 proteins that apparently favor an eocyte ToL (thereafter called eocyte proteins).

**Fig 1:**
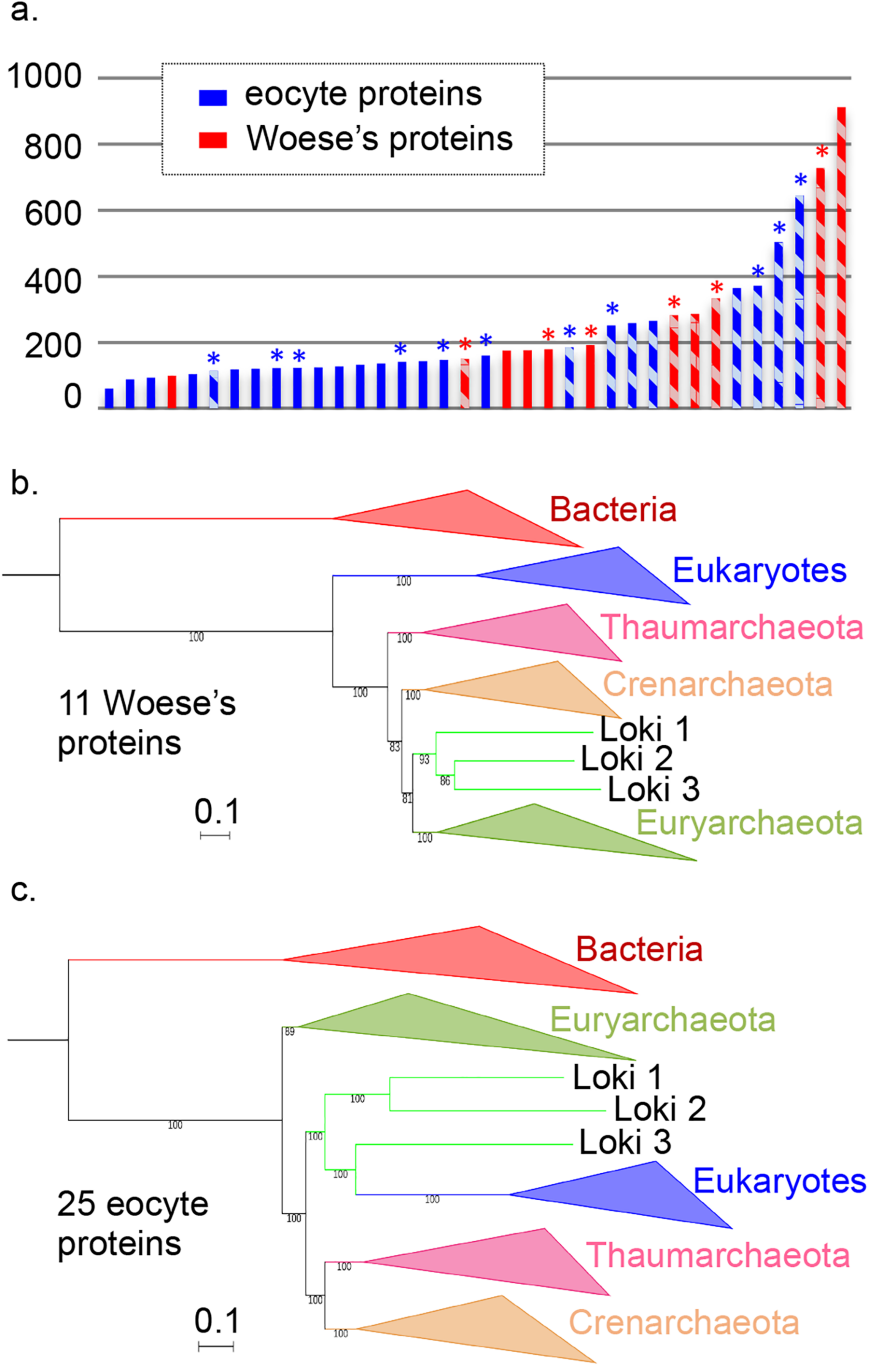
Comparison and concatenation of different subsets of the 36 universal proteins. **a.** Diagram of the amino-acid lengths of the 36 universal proteins, obtained after alignment and trimming from the curated dataset (details in S1 Table). Ribosomal and non-ribosomal proteins are indicated in solid and hashed-bars, respectively. The markers for which the monophyly of Archaea was obtained in their phylogenetic tree are indicated in red, whereas those related to the paraphyly of Archaea are indicated in blue. * indicates alignments that statistically support in AU test the Woese’s or eocyte topology (in red and blue, respectively) **b.** Maximum Likelihood (ML) phylogenetic tree of the concatenation of the 11 Woese’s proteins (3,499 positions). **c.** ML phylogenetic tree of the concatenation of the 25 eocyte proteins (4,868 positions). Detailed trees in S3 and S4 Figs. The scale-bars represent the average number of substitutions per site. Values at nodes represent support calculated by nonparametric bootstrap (out of 100).

The 11 Woese’s proteins were larger than the average universal proteins and represent a total of 3,499 positions after trimming, compared to the 4,869 positions for the 25 eocytes proteins (Fig 1A). In particular, they included the two largest universal proteins (the A and B DNA-dependent RNA polymerase subunits) and the four largest ribosomal proteins of the dataset. The consensus, internal archaeal phylogeny obtained using conserved archaeal ribosomal or core archaeal proteins [34,38] was recovered in several Woese trees (especially in the case of large proteins).

In contrast with the phylogenies obtained with Woese’s proteins, most of those obtained with the 25 individual eocyte proteins were still poorly resolved and often did not recover the monophyly of the major archaeal phyla (S2 Fig). For example, the monophyly of Euryarchaea was recovered in 9 of the 11 Woese’s trees but only in 8 of the 25 eocyte trees. Notably, Eukarya still branched at very different positions from one eocyte tree to another. The sisterhood between Eukarya and one or several Lokiarchaea was observed in 11 cases instead of 10 in the initial dataset, but again never strongly supported, except for EF2 (100% BS value with Loki 3), and for Kae1/YgjD (74% BS value with Loki 2). Interestingly, we even observed a significant decrease in BS support for the node grouping Loki 1 and 3 with Eukarya in the SecY tree (from 84 to 62%).

The ML tree produced by the concatenation of the 11 Woese’s proteins (Fig 1B, S3 Fig) not only recovered the monopyly of Archaea but also the monophyly of the three major archaeal phyla with 100% BS value. However, we did not recover the TACK (or proteoarchaeal) superphylum [18]. Instead, the archaeal tree was rooted between Thaumarchaeota and all other Archaea with strong support. This rooting was previously obtained in a tree based on conserved archaeal ribosomal proteins rooted with eukaryal sequences [39]. Importantly, in our analysis, we obtained this rooting when bacterial and eukaryal sequences were present together in the same dataset. When we removed the two large RNA polymerase A and B subunits (corresponding to the three proteins A’, A” and B) from our dataset and concatenated the remaining 8 Woese’s proteins, the lokiarchaeal sequences were attracted at the root of Archaea, but we also obtained the monophyly of Archaea and of the three major archaeal phyla (S5 Fig). This indicates that signal supporting the Woese tree is not limited to RNA polymerase proteins.

Concatenation of the 25 eocyte proteins produced a ML tree highly similar to the Loki ancestor tree with strong support (Fig 1C, S4 Fig). However, removal of the longest protein from this subset (EF2) produced a tree that failed to recover the monophyly of Euryarchaeota, with Archaea rooted in *Thermococcales* (with 100% BS value) using Bacteria as outgroup (S6 Fig). This observation indicates that the signal supporting the tree topology obtained with these 25 concatenated proteins probably exhibit some degrees of discrepancy among those proteins.

In order to assess the statistical robustness of the two well-supported phylogenies obtained in ML framework (from the concatenated Woese and eocyte proteins, respectively), we performed Approximately Unbiased test (AU test [40]) on the individual protein alignments. The results (S2 Table) indicated that 6 (1,857 positions) out of the 11 Woese protein alignments significantly reject the eocyte topology while significantly supporting the Woese topology (the others still supporting the latter but not significantly rejecting the former). Among the 25 eocyte protein alignments, 11 (2,750 positions) significantly reject the Woese topology and support the eocyte one (the others not significantly rejecting the Woese topology). None of the proteins we grouped in one or the other set supports the other topology. The two sets of alignments that were statistically relevant for one or the other topology according to the AU test (i.e. the 6 Woese and the 11 eocyte protein alignments, hereafter mentioned as AU-relevant) were concatenated and tree reconstruction was performed by both ML (LG model) and Bayesian inference (BI) analyses (CAT-GTR model)(S7–S10 Figs). As expected, the concatenated 6 AU-relevant Woese proteins support the Woese’s tree of life whereas the concatenation of the 11 AU-relevant eocyte proteins supports the eocyte’s one. The congruence of the results through the two approaches strongly supports the actual presence of conflicting data within the different markers. BI analysis of the concatenated 19 remaining protein alignments (those that were not relevant in AU test) did not yield conclusive result, but the ML tree displayed a three-domain topology with the Loki at the base of the Archaea (S11 Fig).

An additional AU test was made to check the robustness of most of the single genes phylogenies obtained in ML framework. The results (S3 Table) suggest that the different topologies obtained were not the result of stochastic variation, supporting the existence of genuine different stories among the 36 markers.

### Multiple signals within the Lokiarchaea genome

The fact that the position of the different lokiarchaeal proteins in the 36 individual trees remained highly variable after the removal of FES suggested different origins for some of these proteins in the three different Loki. This hypothesis was supported by examination of the individual trees. For instance, among the 71 loki proteins with archaeal affinity in our phylogenies without FES, we observed 15 proteins that branched within or close to Thaumarchaeota, suggesting that, besides possible horizontal gene transfers, some of these proteins could correspond to thaumarchaeal sequences, which represent up to 9% of the archaeal population present in the Loki Castle sample [14]. We thus decided to assess the quality of the Loki 1 genome reconstruction using the recent tools CheckM and Anvi’o [41,42] that were developed to analyze the completeness and contamination of genomes using lineage-specific marker genes (145 and 162 markers, respectively) (see Methods for more details; results in S12A Fig). They both estimated the lokiarchaeon 1 genome to be between 90.29% and 92.6% complete (CheckM and Anvi’o, respectively) in agreement with Spang and colleagues’ estimation (92%). However, they also evaluated the contamination to be superior to 45% (45.15 and 56.8% of contamination, respectively). CheckM additionally determined that the reconstructed Loki 1 genome was highly heterogeneous (index of 78.21). Similarly, using Anvi’o, we observed that Loki 1 contigs could be grouped in six different sets by hierarchical clustering based on their tetra-nucleotide sequence composition and their differential reads coverage across the different sequencing runs (S12B Fig). Selecting only three of them (sets 4 to 6) already allows to reach a completeness of 90% (with a contamination at 14%), but adding the set that accounts for the second largest number of archaeal markers (set 2) would only add 2% of completeness to the genome while bringing the contamination to more than 56%. Considering that all those sets are included in Loki 1 genome, these results suggest that the Lokiarchaeum (Loki 1) genome is a chimera of related strains and contaminated sequences (see Methods part for more details).

The genome quality classification scheme, proposed by CheckM authors [41], establishes a threshold at 15% above which a genome has to be considered as “very highly” contaminated. As a comparison, the contamination of Thorarchaea genomes, evaluated between 4.7 and 6.7%, would be considered as medium [30]. The quality of the Loki 2 and Loki 3 genome reconstructions could not be verified, because for these two lineages only 21 and 34 coding sequences (CDS) were available on the NCBI database (S4 Table), and analysis of the available reads coverage by reads mapping and BLASTn search against the SRA database were not conclusive (see Methods for more information).

### Chimeric signals among lokiarchaeal Elongation Factor 2 (EF2) proteins

**Fig 2:**
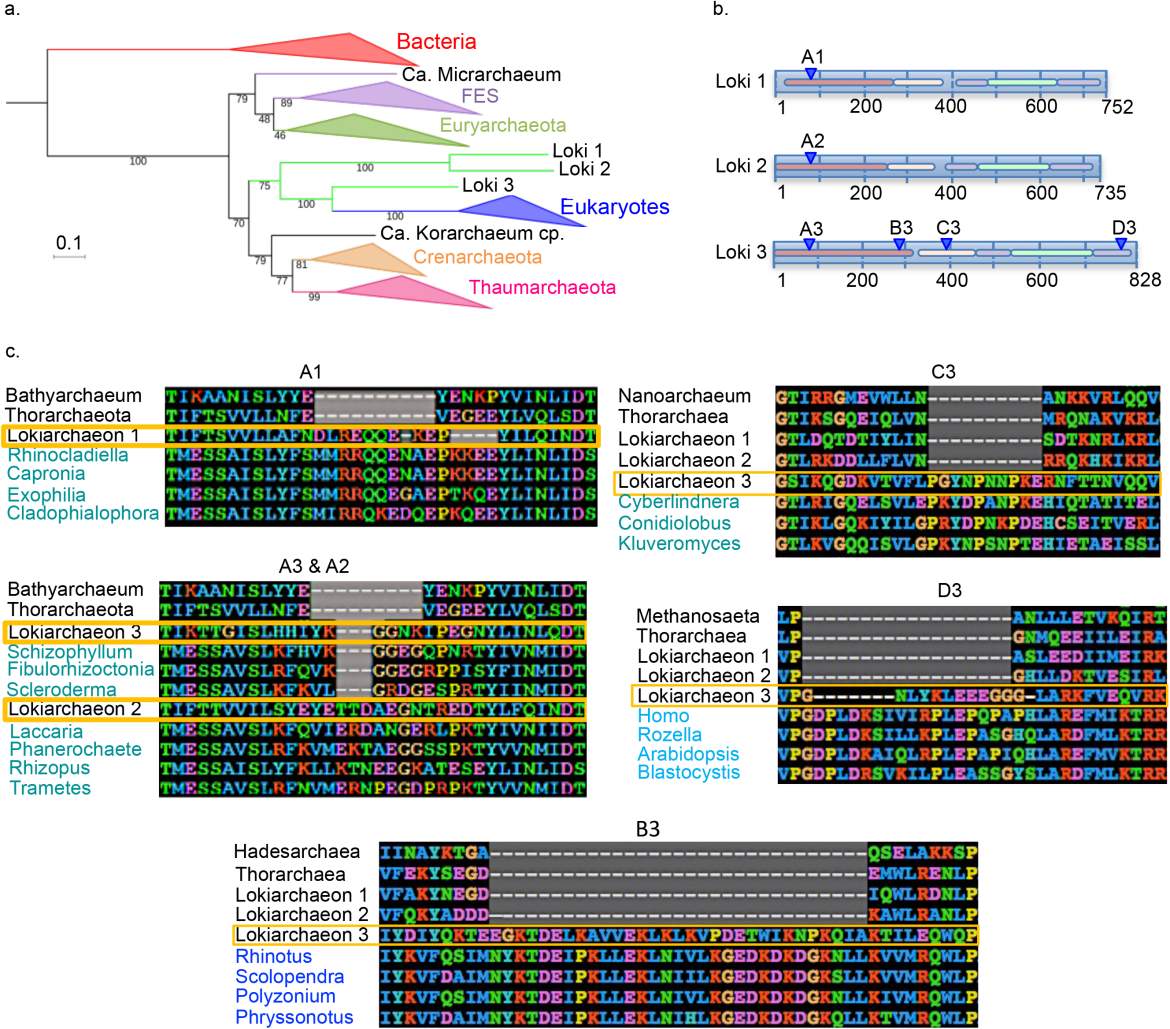
Eukaryotic-like insertions in the lokiarchaeal EF2 proteins. **a.** ML phylogenetic tree of EF2 with the initial dataset (626 positions). The scale-bar represents the average number of substitutions per site. Values at nodes represent support calculated by nonparametric bootstrap (out of 100). **b.** Schematic representations of the three lokiarchaeal EF2 proteins with the five different domains indicated by colored lines and the positions of the specific eukaryotic insertions indicated blue triangles. **c.** Alignments of the 6 observed insertions of the EF2 protein (arCOG01559) are showed. Organisms’ names corresponding to Archaea and Eukarya are respectively indicated in black and blue, and lokiarchaeal sequences are surrounded in yellow. The A1, 2, 3 and C3 insertions are aligned with eukaryotic Ria sequences (EF2 paralog), whereas B3 and D3 are aligned with eukaryotic EF2 and Snu5 sequences (EF2 paralog), respectively. Detailed alignments in S13–S16 Figs.

During our inspection of individual phylogenies obtained with or without FES, we noticed that a single protein, EF2, yielded ML trees similar to the Loki ancestor tree published by Spang and coworkers (Fig 2A, S1 and S2 Figs)[14]. In addition, whereas all other universal lokiarchaeal proteins (including Loki 1 and Loki 2 EF2) gave other Archaea as first hits in BLASTp analyses, BLASTp using the EF2 protein of Loki 3 as query only retrieved eukaryotic sequences (mainly fungi). This prompted us to carefully examine the alignments of lokiarchaeal EF2 proteins to look for specific sequences that could explain this observation. We readily identified several putative insertions shared by Eukarya and one or several Lokiarchaeal EF2 sequences from our multiple alignment. Another alignment software, PRANK, confirmed the presence of these regions matching with eukaryotes, even if obviously aligned differently. Three different insertions were located at the same position in the N-terminal regions of the three Loki (A1, A2, A3), and three others (B3, C3 and D3) were located in the central and C-terminal regions of Loki 3 EF2 protein (Fig 2B, S13–S16 Figs). Interestingly, these insertions are missing in EF2 sequences from Thorarchaeota, a putative archaeal phylum (also obtained from metagenomic data) sister group to lokiarchaeon 1 in a tree based on the concatenation of 16 ribosomal proteins [30]. All these lokiarchaeal-specific insertions were large (between 5 and 31 amino-acids long) and were surrounded by regions highly conserved within all Archaea, including FES. This conservation induced the presence of strong anchors that enabled us to retrieve the insertions and their surrounding positions from the alignment, and used them as queries in BLASTp analyses to search sequences matching best with these regions. Alignments including the best hits are presented in Fig 2C (S13–S16 Figs).

The N-terminal insertion located at the same position in the three Loki (A1, A2 and A3) was different in size and sequence and aligned better with the eukaryotic protein Ria1p, an EF2 paralog involved in ribosomal biogenesis, than with eukaryotic EF2 themselves (S13 Fig). Insertion C3 in Loki 3 also better aligned with Ria1p, whereas insertions B3 and D3 better aligned with EF2 and Snu5, another EF2 paralog, respectively (S14–S16 Figs). In all cases, these insertions better aligned with proteins from fungi than with other eukaryotic proteins. This was especially visible for insertion C3 that shares 7 out of 10 amino-acids of the same insertion with the yeast *Cyberlindnera jardinii* Ria1p (S15 Fig). The non-conservation of the N-terminal insertion between the three Loki and between the different insertions of Loki 3 that correspond to different eukaryotic proteins and/or phyla, strongly suggests that these insertions are not synapomorphies testifying for a Lokiarchaea-Eukarya affiliation, but more likely resulted from contamination of the three lokiarchaeal EF2 with sequences of eukaryotic origin that were present in the DSAG-enriched sample. We failed to detect such long indels in the other 87 universal proteins of Lokiarchaea.

The fact that Loki 1 and Loki 2 EF2 only contain one of the four insertions present in Loki 3 and that the different insertions in Loki 3 EF2 better aligned with different eukaryotic proteins suggests a chimeric organization of these proteins. Importantly, since these insertions were removed from the trimmed alignments, this suggests that Loki EF2 sequences, and especially Loki 3, probably still contain hidden patches of eukaryal sequences responsible of the attraction. We tentatively tried to detect these potential chimeric sites by different approaches, including by using HMM profiles, but differentiating them from genuine similar sites was particularly complex (Loki 1 and 2 EF2 have around 55% of identic sites, but only ca. 35% to Loki 3) and did not yield any conclusive result.

**Fig 3:**
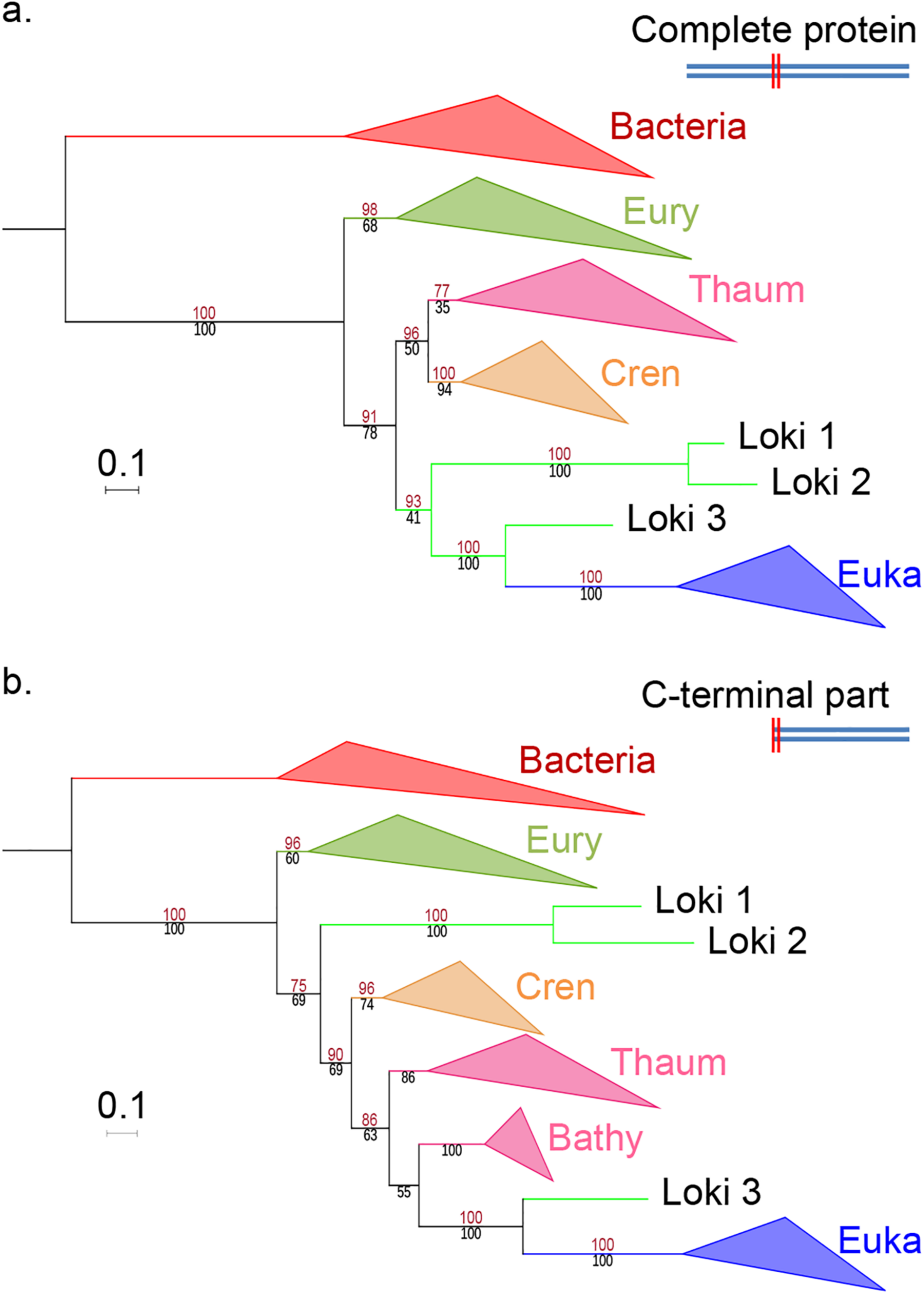
EF2 phylogenetic trees, based on the curated dataset after inclusion of bathyarchaeal sequences. **a.** ML phylogenetic tree of the complete sequence (626 positions). **b.** ML phylogenetic tree of the C-terminal part only (394 positions). Eury, Thaum, Cren and Euka stand for Euryarchaeota, Thaumarchaeota, Crenarchaeota and Eukaryotes. Detailed trees in S17 and S18 Figs. The scale-bars represent the average number of substitutions per site. Values at nodes represent support calculated by nonparametric bootstrap (out of 100) and ultrafast bootstrap approximation (1,000 replicates), in black and red, respectively.

We however observed that after trimming the sequences, Loki 1 and 2 N-terminal portions (up to approx. 230 amino-acids) matched to Lokiarchaea-related genomes in BLASTp searches, followed by Thaumarchaea and Cren- or Euryarchaea, whereas the same portion in Loki 3 gave best hits to various Euryarchaea. The rest of the EF2 sequence (approx. 370 aa) matched better to Lokiarchaea-related genomes and Crenarchaea for Loki 1 and 2, but to Bathyarchaea (a new putative phylum closely related to Thaumarchaea) followed by crenarchaeal sequences and even some eukaryotes for Loki 3. This suggested that the Loki EF2 were indeed reconstructed by combining at least two portions of sequences from different origins. To verify the putative relationship between Loki 3 EF2 and Bathyarchaea, we added bathyarchaeal EF2 sequences to the dataset and generated new ML phylogenies on the entire sequence and its putative two sub-portions. The trees obtained with the entire protein or its N-terminal portion still displayed the Lokiarchaea-Eukarya association (Fig 3A, S17 and S18 Figs). In contrast, the tree obtained with the C-terminal moiety showed only Loki 3 as sister group to Eukaryotes, while Loki 1 and 2 were located between monophyletic Euryarchaeota and Crenarchaeota (Fig 3B, S17 and S18 Figs). This supports the hypothesis of a global chimeric organization, with the largest portion of Loki 3 EF2 containing more eukaryotic-like signal, explaining its attraction toward Eukaryotes.

Spang and co-workers excluded *a priori* Eukaryotic contamination in the lokiarchaeal genomes because they did not detect 18S rRNA in this sample [14]. However, they also reported that sequences related to Mimiviruses were present in the Loki sample [14], suggesting the presence of DNA from their eukaryotic hosts. In fact, several analyses have detected various types of eukaryotes, especially fungi, in the deep subseafloor sedimentary biosphere [43–48]. The possible contamination hypothesis would also be compatible with the fact that in the Loki Castle environmental sample, up to 9% of the relative abundance of archaeal 16S reads correspond to Thaumarchaeota (Thaumarchaea, Bathyarchaea)[14].

The putative presence of short contaminating sequences in lokiarchaeal genomes could be explained by the fact that DNA used to reconstruct the Loki 2 and Loki 3 genomes was obtained using multiple displacement amplification (MDA). Sequences obtained after MDA were also used in the reconstruction process of the Loki 1 genome. MDA is prone to generate chimeric sequences and requires a correction step [49,50]. This step was performed by Spang *et al*. with a version of the SPADes software [51] designed for single-cell sequencing projects that can only be used in metagenomic analyses “*at your own risk*”, as stated in the user’s manual. Notably, chimeric sequences produced during single-cell genome assembly processes involving MDA have been shown to be quite small, with 98% being less than 250 nucleotides [49], i.e. in the range of the indel sizes that we detected in EF2.

Interestingly, EF2 is the only marker out of the 36 used in the concatenantion that has been grouped by Anvi’o in the set of contigs that critically increases the contamination level of Loki 1 genome without significantly improving the completeness (set 2; S12 Fig and S4 Table).

### Deep influence of EF2 from Loki 3 on the topologies obtained

**Fig 4:**
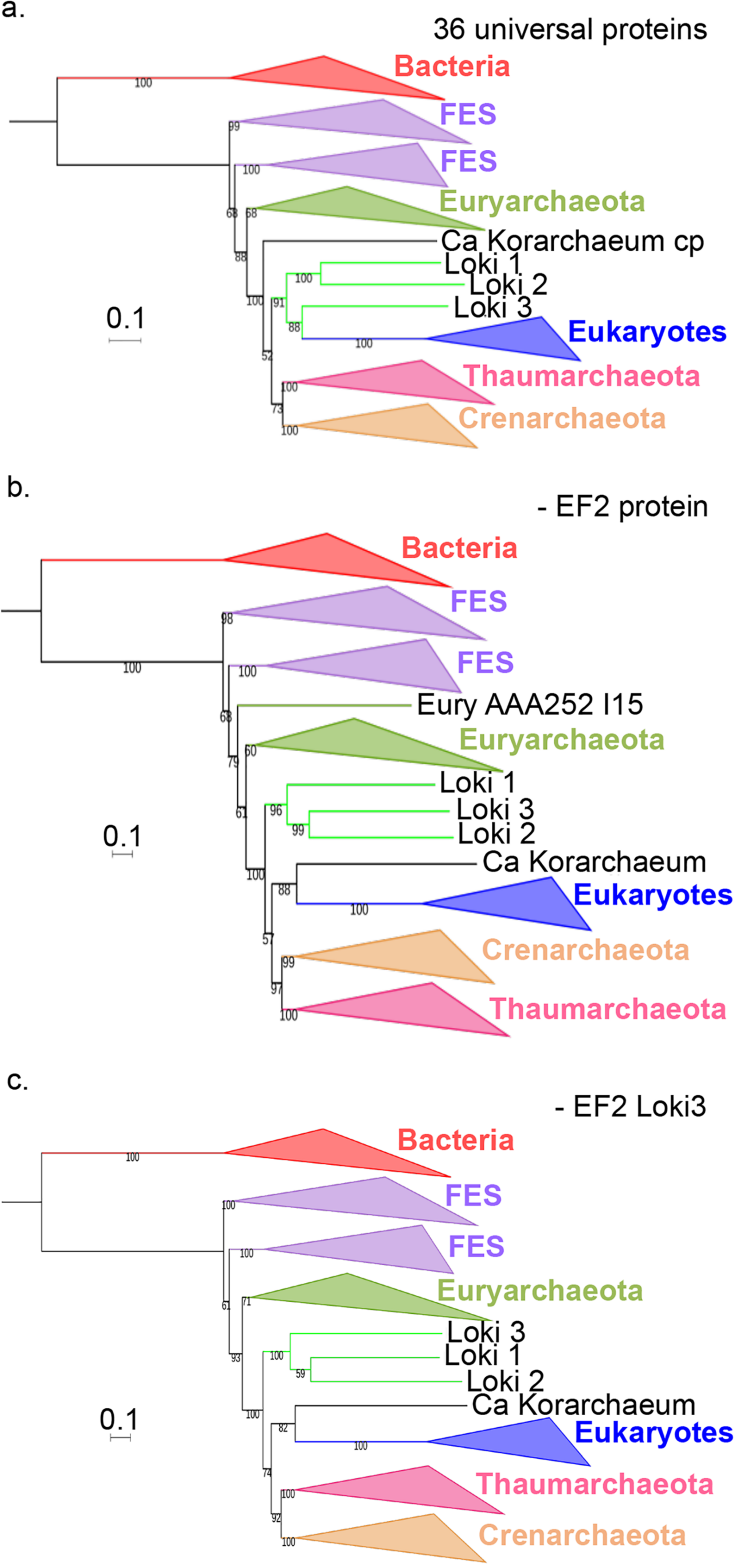
Impact of the EF2 protein on the original concatenated alignment. **a.** ML phylogenetic tree of the original concatenated alignment of the 36 markers (10,547 positions). **b.** ML phylogenetic tree of the original concatenated alignment after removal of the EF2 protein (9,831 positions). **c.** ML phylogenetic tree of the original concatenated alignment after removal of the Loki 3 EF2 sequence (10,547 positions). Detailed trees in S19, S21 and S23 Figs. The scale-bars represent the average number of substitutions per site. Values at nodes represent support calculated by nonparametric bootstrap (out of 100).

**Fig 5:**
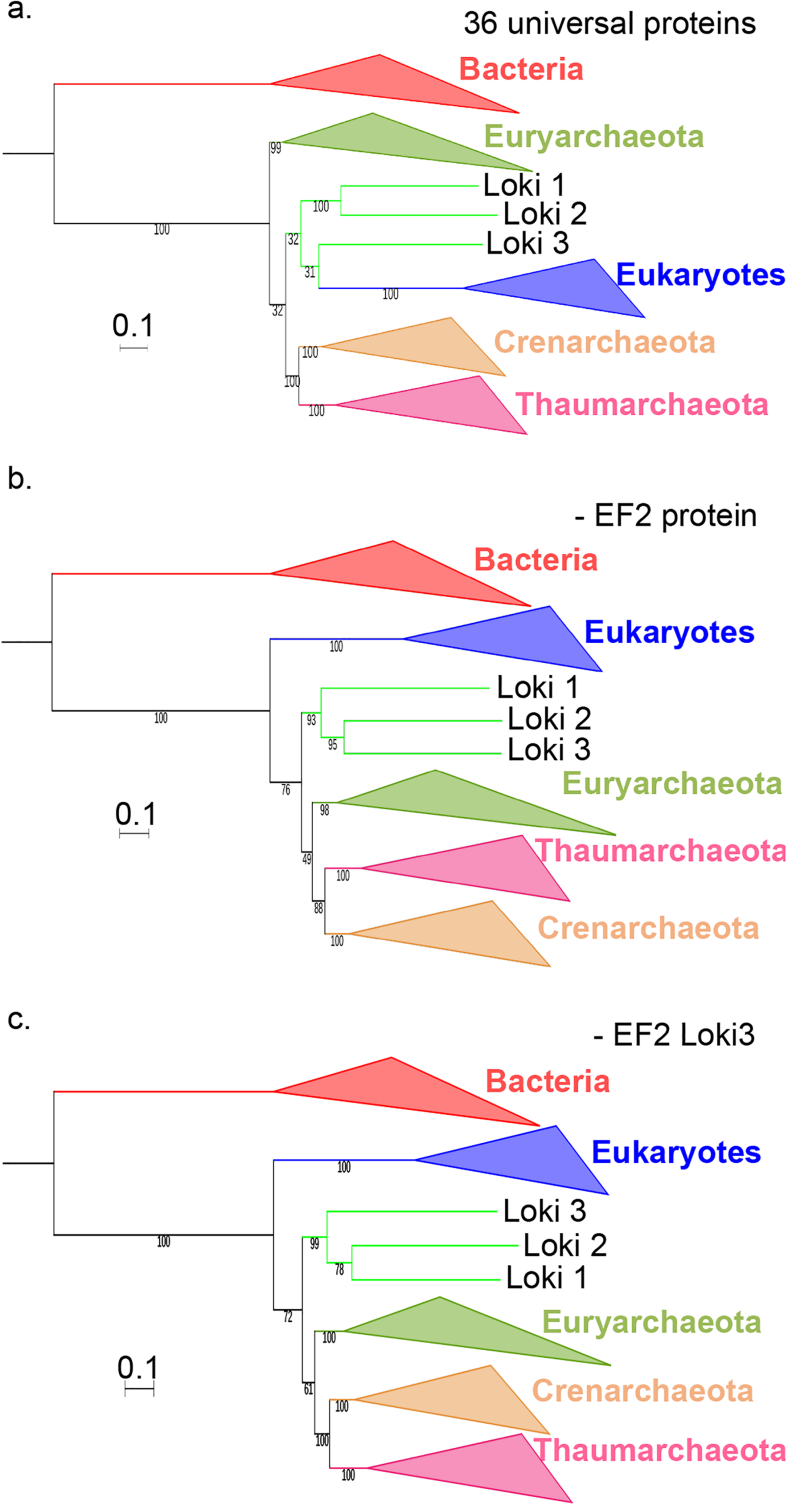
Impact of the EF2 protein on the concatenation of the curated datasets. **a.** ML phylogenetic tree of the concatenated curated datasets (8,367 positions). **b.** ML phylogenetic tree of the concatenated curated datasets after removal of the EF2 protein (7,724 positions). **c.** ML phylogenetic tree of the concatenated curated datasets after removal of the Loki 3 EF2 sequence (8,425 positions). Detailed trees in S20, S22 and S25 Figs. The scale-bars represent the average number of substitutions per site. Values at nodes represent support calculated by nonparametric bootstrap (out of 100).

Our analyses suggesting the presence of hidden eukaryotic contamination in the three Loki EF2 proteins prompted us to compare the concatenation of the 36 universal proteins with and without EF2. We decided to remove EF2 from the concatenations both with and without FES (i.e. the original concatenated alignment and the curated concatenated datasets, respectively). For the 36 concatenated proteins with and without FES (controls), we obtained ML phylogenies highly similar to the Bayesian Loki ancestor tree (Figs 4A and 5A, S19 and S20 Figs). However, the BS values at nodes supporting the Loki ancestor topology were slightly lower in the tree without FES (S20 Fig), indicating that this topology was partly supported by the presence of FES in the Spang *et al*. dataset. Surprisingly, we observed that *M*. *kandleri* was correctly located as sister group to *Methanobacteriales* in the ML tree with FES (S19 Fig), whereas it was mis-located at the base of Archaea in the Bayesian tree published by Spang and coworkers [14].

Remarkably, the Lokiarchaeota-Eukarya affiliation was lost after removing EF2 in our ML phylogenies with and without FES (Figs 4B and 5B, S21 and S22 Figs). Eukarya became sister group to “*Ca*. K. cryptophylum” in the tree with FES, and the three Lokiarchaea were now all located between Euryarchaeota and other Archaea (Fig 4B, S21 Fig). In the tree without FES, Archaea became monophyletic with 76% BS value (Fig 5B, S22 Fig). In this tree, the three Lokiarchaea branched between Eukarya and Archaea. These results indicate that some signal in EF2 is sufficient not only to trigger the specific Lokiarchaeota-Eukarya association, but also to break the monophyly of Archaea.

Since our indel analysis suggested that Loki 3 was more contaminated than Loki 1 and 2, we built a ML tree after removing Loki 3 sequences from the original concatenated alignment. Our results indicate that the removal of Loki 3 was sufficient to break the Lokiarchaeota-Eukarya association (S23 Fig). “*Ca*. K. cryptophylum” was again sister group to Eukarya whereas Loki 1 and 2 branched between Euryarchaea and other Archaea with maximum support.

As an additional step, we removed only Loki 3 EF2 from the original concatenated alignment and from the FES-curated datasets and kept the 89 other lokiarchaeal proteins. Stunningly, removal of this unique protein from the original concatenated alignment was again sufficient to break the Lokiarchaeota-Eukarya association and to produce a tree similar to those obtained without EF2 or without Loki 3 proteins (Fig 4C, S24 Fig). Removing it from the curated dataset led again to a tree displaying the monophyly of Archaea with significant support, and the three Loki at the most basal position in Archaea (Fig 5C, S25 Fig), similarly to the tree obtained with the curated dataset without EF2 protein. This clearly indicates that a single protein, out of the 90 lokiarchaeal ones, is sufficient to group the Lokiarchaea and the Eukaryotes together, and also to favor the eocyte tree in the absence of FES.

The impact of EF2 is also observable on the concatenation of the eocyte protein alignments that were statistically relevant in AU test (S26 and S27 Figs). While BI analysis of the concatenated 11 AU-relevant eocyte protein alignments yielded a highly supported Loki ancestor tree (S10 Fig), removing EF2 from it led to a globally less supported Bayesian tree with paraphyletic Euryarchaeota (S27 Fig). Interestingly, only Loki 3 was grouped with Eukaryotes, whereas Loki 1 and 2 were located within Archaea, sister group to a clade grouping Thaumarchaea and Crenarchaea.

### Comparison between Lokiarchaeota and Thorarchaeota

After the publication about Lokiarchaeota, three “partial to near-complete” genomes have been reconstructed from metagenomic data collected from estuary sedimentary samples, and were grouped within the candidate “Thorarchaeota” phylum, based on phylogenetic analyses of the 16 rRNA gene and ribosomal proteins [30]. In a ML tree based on the concatenated alignment of 16 ribosomal proteins and using Eukarya as outgroup, “*Candidatus* Thorarchaeaota archaeon” were shown to be sister group to Lokiarchaeota.

Considering this suggested relationship, we checked for the 36 universal proteins in the thorarchaeal genomes. We decided to focus on the two most complete genomes (SMTZ1-83 and SMTZ1-45, ~90% and ~87% complete, respectively), like Seitz and colleagues for their concatenation-based analysis. We could find 34 and 27 universal proteins out of the 36 used by Spang *et al*. in these two genomes, respectively (the two proteins systematically missing being the ribosomal proteins S3 and S4; S4 Table), and replaced the lokiarchaeal sequences by the thorarchaeal ones in the corresponding FES-curated datasets.

**Fig 6:**
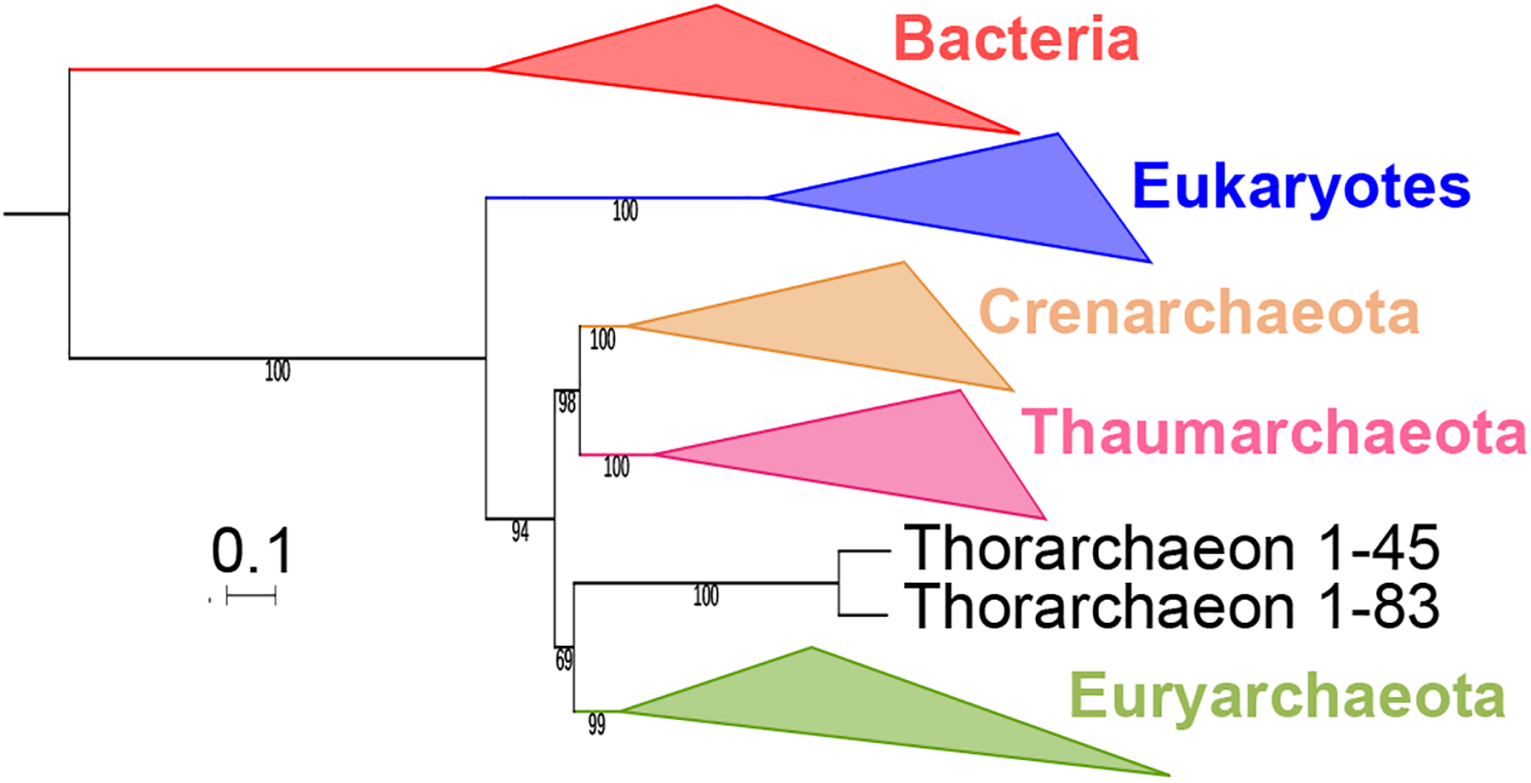
Position of *Candidatus* Thorarchaeota archaea in the Tree of Life. ML phylogenetic tree of the concatenated alignments of the 34 markers present in the two most complete thorarchaeal genomes. Detailed tree in S28 Fig. The scale-bar represents the average number of substitutions per site. Values at nodes represent support calculated by nonparametric bootstrap (out of 100).

We performed a ML analysis of the concatenated alignments of the 34 proteins without FES and obtained a tree in which Archaea were monophyletic and the “*Ca*. Thorarchaeaota archaeon” were sister group to Euryarchaeota (Fig 6, S28 Fig). Since the EF2 protein was included in the concatenation, and considering the probable relationship between Thorarchaeota and Lokiarchaeota, this result supports the idea that lokiarchaeal EF2, and more precisely the Loki 3 EF2 protein, brought a strong bias in the concatenation performed by Spang and co-workers. Notably, and as mentioned before, thorarchaeal EF2 protein sequences do not have any of the indels we could find in the lokiarchaeal EF2, suggesting a potentially better global quality of the thorarchaeal genomes.

### RNA polymerase phylogeny supports the monophyly of Archaea

In order to avoid, as much as possible, the pitfalls above-mentioned concerning the concatenation of many proteins from likely chimeric genomes, and to know if the position we obtained for the Thorarchaea by concatenating 34 universal proteins matches with Lokiarchaea, we decided to perform a robust phylogenetic analysis of the DNA-dependent RNA polymerase using a new species dataset. Indeed, we suspected that the original set of species used by Spang and co-workers was far from optimal for tree reconstruction, even after the removal of FES, because it was strongly unbalanced with 10 Bacteria, 10 Eukarya and 84 Archaea. This imbalance could lead to technical issues in downstream analysis such as alignment, trimming and selection of phylogenetically informative regions for tree reconstruction [26]. To avoid similar issues, we constructed a new set of species sampling 39 different taxa from each of the three domains, trying best to select a range of species covering all major recognized phyla within each domain and only using sequences obtained from well-characterized genomes and avoiding archaeal FES (S5 Table).

This choice of the DNA-dependent RNA polymerase was motivated because they are the longest universal proteins (more than 1,200 amino acids for each of the two largest subunits). Furthermore, we have previously shown that this enzyme is a reliable marker for archaeal phylogeny since the archaeal RNA polymerase phylogeny is fully congruent with the phylogeny obtained with ribosomal proteins, except for the fast-evolving *M*. *kandleri* [27,32]. In fact, considering the multimeric nature of the RNA polymerase, one could assume a rate of substitution relatively homogeneous for the two large subunits that are both involved in the catalytic activity of the protein and both important to conserve the global structure and the interaction with DNA and RNA. These characteristics stand well compared to ribosomal proteins that are much smaller and occupy external positions on the ribosome, explaining why some of them could lack phylogenetic signal to analyze the divergence between domains.

Archaeal RNA polymerase A subunits exist in two versions, a single polypeptide (A-type) as in most Bacteria and Eukarya, and a two subunits version (A’A”-type) in which the A subunit is split (S29 Fig). The A’A”-type is present in Euryarchaeota and Crenarchaeota, whereas the A-type is present in Thaumarchaeota and Korarchaeota. Surprisingly we found both types within the Lokiarchaeota, with Loki 1 and 2 containing the A’A” type (although the A” subunit is missing for Loki 2) whereas Loki 3 contains the A-type, once more confirming the diverse origin of universal lokiarchaeal proteins. The Loki 1 and 2 RNA polymerases A are closely related to each other and to Thorarchaea, suggesting that these proteins are the *bona fide* lokiarchaeal RNA polymerases. In contrast, the Loki 3 RNA polymerase A subunit is divergent from the A’ and A” subunits of Loki 1 and seems more related to the fused Thaumarchaea and Bathyarchaea sequences, suggesting that it corresponds to a contaminant. We thus decided to consider the only complete Lokiarchaeal A’A”-type RNA polymerase (Loki 1) as the representative of the Lokiarchaeota for our phylogenetic analysis.

**Fig 7:**
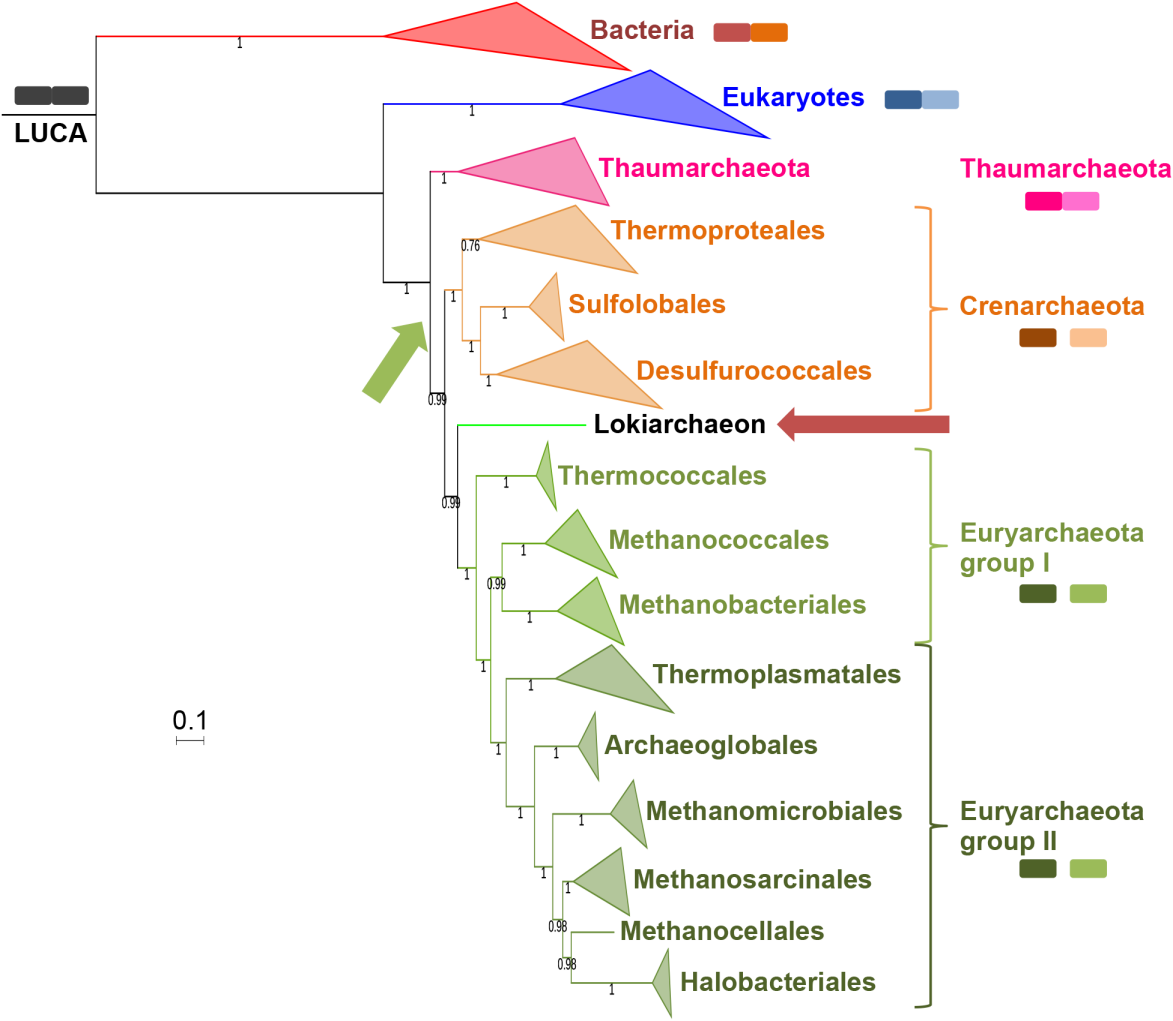
RNA polymerase phylogeny. Bayesian phylogeny (LG model + Γ4) of the concatenated alignments of the two largest RNA polymerase subunits (1,463 positions) from an equal number (39) of Archaea, Eukaryotes (blue) and Bacteria (red). Among the Archaea, Thaumarchaeota, Crenarchaeota, group I Euryarchaeota and group II Euryarchaeota are indicated in pink, orange, light-green and dark-green, respectively. Values at nodes represent the Bayesian posterior probabilities. Detailed tree in S30 Fig. See S31 Fig for CAT-GTR model tree, and S32 Fig for ML tree. The scale-bar represents the average number of substitutions per site. A red arrow indicates the Lokiarchaea position in the tree. The A subunit status (split or fused) is indicated by adjacency of colored squares. The green arrow indicates the position of the split event among the archaeal phylogeny.

We performed a ML and two independent BI analyses (LG and CAT-GTR models) of the concatenation of RNA polymerase A and B subunits using our new species dataset. All trees were highly similar, they all recovered the monophyly of Archaea and were fully congruent with the consensus internal archaeal phylogeny [34,38] (Fig 7, S30–S32 Figs). The internal topologies of domains Bacteria and Eukarya were also rather well resolved. In particular, we recovered the monophyly of Proteobacteria in Bacteria and of Amorpha in Eukarya. The BI analyses, after convergence, only slightly improved support of some basal positions.

In all trees, the RNA polymerase of Lokiarchaea branched as a sister group to Euryarchaeota, with strong support (77% BS in ML; 0.99 and 0.95 posterior probabilities in BI with LG and CAT-GTR models, respectively) (S30–S32 Figs). In agreement with this result, Spang *et al*. previously noticed that most lokiarchaeal proteins with archaeal affinity in Best-BLASTP-hit analysis were related to Euryarchaeota (75%), as opposed to other phyla (13% and 7% for Crenarchaeota and Thaumarchaeota, respectively). We also observed the same tendency for proteins with archaeal affinity whose genes are located on the contig containing the 16S DSAG rRNA gene (64%, 21% and 10% for Euryarchaeota, Crenarchaeota and Thaumarchaeota, respectively). Interestingly, the phylogenetic position for Lokiarchaea proposed here is congruent with the position of the Thorarchaeota in the ML tree obtained from the concatenation of 34 universal proteins (Fig 6). It is also coherent with an analysis of its metabolism based on enzymes with clear archaeal affinity, which has suggested that Loki was a hydrogen producer with a metabolism close to those of autotrophic Euryarchaea [23].

The archaeal domain was rooted in the branch leading to Thaumarchaeota in all trees, as previously observed with the concatenation of the 11 Woese’s proteins (Fig 1B). Importantly, this rooting explains the distribution of A and A’A”-type RNA polymerases by a single splitting event that has taken place after the divergence between “*Ca*. K. cryptophylum” and other archaea, whereas alternative roots require additional events of either fusion and/or split (Fig 7). In particular, four events (splits and/or fusions) are necessary to explain the distribution of A and A’A”-type RNA polymerases in the Loki ancestor tree. This is clearly less parsimonious because such events seem to be rare in the history of RNA polymerases. A secondary split only happened in Cyanobacteria and Mimiviridae, but at different position, and a secondary fusion in Pacearchaeaota, a recently described phylum of fast-evolving archaea with small genomes (S29 Fig)[52].

We performed ML phylogenetic analyses of the RNA polymerase after integration of the sequences from Bathyarchaea, “*Candidatus* Thorarchaeaota archaeon”, Hadesarchaea and candidate Division MSBL1 Archaea (all obtained from metagenomic data) [53–55], using both Bacteria and Eukarya as outgroups. In these new trees, the newly added sequences had identical relative positions (S33–S35 Figs). We obtained a strong support in favor of a clade grouping Thaumarchaea, Aigarchaea and Bathyarchaea. We thus suggest considering all these lineages as members of the phylum Thaumarchaeota, to be consistent with the original definition of this major archaeal phylum that was proposed to include all archaea previously considered to be mesophilic Crenarchaeota, as long as they form a monophyletic group [39]. We also obtained a strong support for a clade grouping Thorarchaea with Lokiarchaea that branched between Crenarchaeota and Euryarchaea whereas Hadesarchaea and MSBL1 branched between Lokiarchaeota/Thorarchaeota and Euryarchaeota.

**Fig 8:**
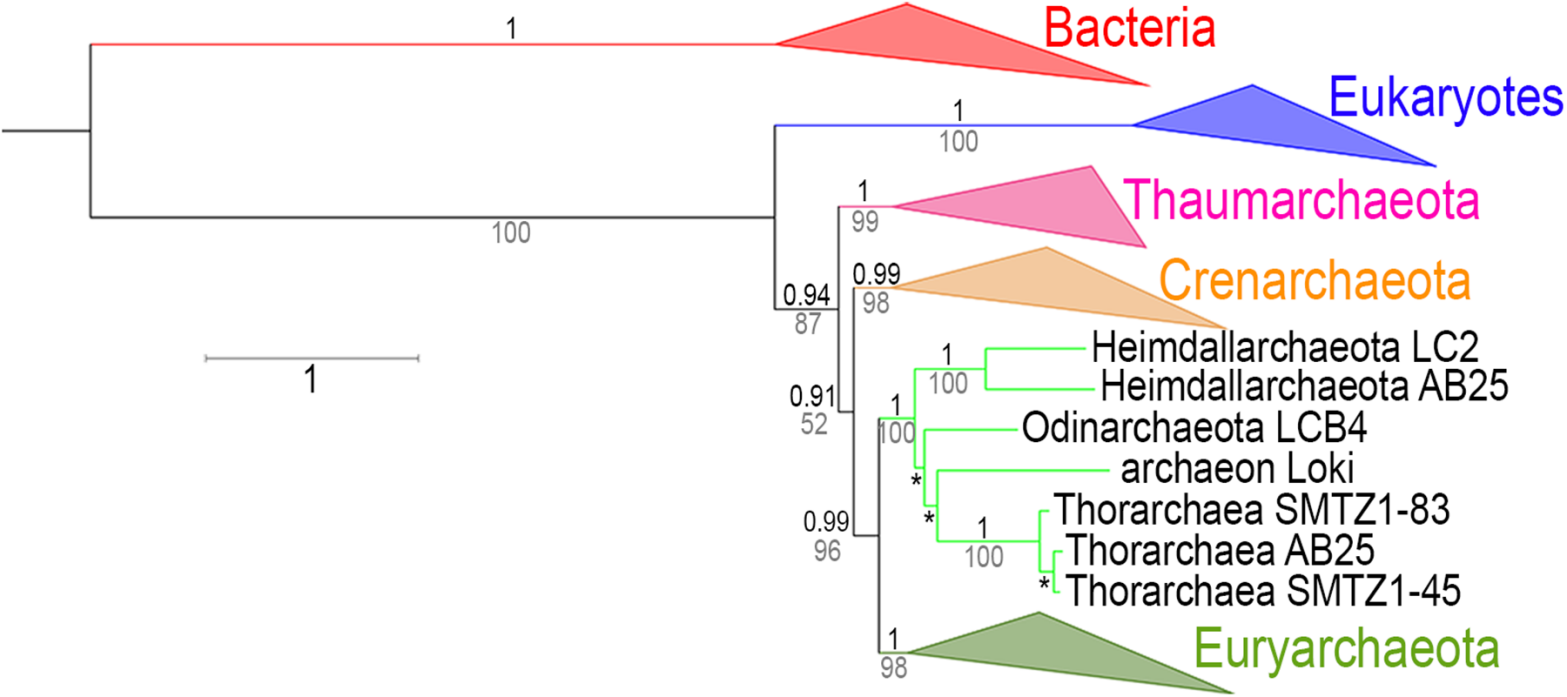
RNA polymerase phylogeny with the Asgards archaea. Tree representing the combined phylogenies obtained in ML (LG model + Γ4) and Bayesian inference (CAT-GTR model) analyses of the two largest RNA polymerase subunits after inclusion of the Asgards archaea in the dataset (detailed trees in S36 and S37 Figs). Bacterial and eukaryotic sequences are indicated in red and blue, respectively. Among the Archaea, Thaumarchaeota, Crenarchaeota, and Euryarchaeota are indicated in pink, orange, and olive-green respectively. Values over the branches (in black) correspond to the posterior probabilities (PP) of the corresponding nodes obtained from Bayesian inferences, while the values below the branches (in grey) represent supports calculated by non parametric bootstrap (BS) from the ML analysis. Branch lengths in this tree are derived from the tree obtained from the Bayesian inference (S37 Fig), and the scale-bar represents the average number of substitutions per site. From base to tips, the three * correspond to 0.95/53, 0.92/61, and 1/100, respectively (PP/BS).

Finally, including sequences from recently described genomes related to Lokiarchaeota (forming altogether the putative Asgard superphylum [31]) to our RNA polymerase dataset supports our conclusion. The ML and BI phylogenies obtained displayed the same topology, with the monophyly of the Asgards (including Lokiarchaea and Thorarchaea) at the base of the Euryarchaeota with strong support (Fig 8, S36 and S37 Figs) and the rooting of the archaeal tree between Thaumarchaeota and all other Archaea. We suggest that Thorarchaea and Lokiarchaea, and probably the other Asgards, should not be considered as different new phyla but either as members of the same new phylum or as early branches of the phylum Euryarchaeota.

## Discussion

Our detailed reanalysis of the Spang *et al*. dataset revealed that the Woese *versus* eocyte topology was likely determined by a combination of i) the choice of protein markers to include in the supermatrix, and ii) the inclusion of fast-evolving species (FES) in the dataset that could lead to biases. In addition, we show here that the emergence of Eukarya specifically within paraphyletic Lokiarchaeota, and to some extent the global Eukarya-Lokiarchaeota association, was likely due to the lokiarchaeal EF2 protein and its very probable chimeric structure. We identified insertions similar to eukaryotic EF2 proteins and paralogs in the lokiarchaeal EF2 proteins, especially in Loki 3 EF2 protein. The artefactual branching of lokiarchaeal EF2 proteins between Archaea and Eukarya could originate from sequences of EF2 paralog from eukaryotes and/or other Archaea present in the Arctic sample. These sequences may have been acquired by horizontal gene transfer followed by recombination and/or during the *in silico* assemblage, by combining archaeal and eukaryotic sequences. Notably, it was possible to break the Lokiarchaea-Eukarya association by removing this unique protein from the initial data set, but also to retrieve the monophyly of Archaea when removing it from the FES-curated dataset. Interestingly, it has been shown that EF2 has probably a very complex evolutionary history, with at least 8 duplications in Bacteria and two in Eukaryotes (predating their last common ancestor), and a possible EF2 duplication before LUCA could not be excluded [56].

Our conclusions over the impact of this single protein, EF2, on the global topology reminds the observations made recently by Shen and colleagues [57]. These authors have studied the distribution of the phylogenetic signal in a data matrix used to identify the earliest-branching phylum among Metazoa. They showed that the resolution of specific nodes in ML analyses can be very sensitive to small subsets of very large data matrice. They also demonstrated that the resolution of some branches can rely on a single gene (or even a few sites) and that its removal from concatenation analyses can alter the inferred topology. In our case, we observed the loss of the Eukaryotes-Lokiarchaea affiliation we observed after removing EF2 from our concatenations in both ML and Bayesian analyses. It should be therefore interesting to repeat the simulations performed by Shen and coworkers in a Bayesian framework.

We still recovered eocyte topologies after removing EF2 from the original dataset containing FES and from the 25 eocyte proteins of the curated datasets. This clearly indicates that, beside EF2, other universal proteins favor the eocyte scenario. Which topology is favored by a given protein seems however strongly dependent on the presence of FES in the dataset, and our analysis also revealed that FES present in the dataset favor eocyte trees. Removal of species we presumed to be FES from the initial dataset improved individual phylogenies (somehow supporting their fast-evolving status), and suggested the high heterogeneity between the three Loki. This was confirmed by the results of quality control of the Lokiarchaeum genome by CheckM and Anvi’o that indicate that this genome is highly heterogeneous and very highly contaminated.

In addition to the biases introduced by FES proteins, the choice of markers also strongly influences the outcome of the concatenation, favoring either the Woese or eocyte scenario. Remarkably, our analysis of the 36 universal markers after removal of FES revealed at least two different evolutionary histories within universal proteins. Notably, we obtained highly similar topologies with both ML and Bayesian inferences (CAT-GTR model) in our analyses of the AU-relevant concatenations (Woese and eocyte proteins). This adds credit to these results and confirms that recovering the phylogenetic signal strongly depends on the dataset [9] (taxon sampling, markers selection, so on), either with ML or Bayesian methods.

The fact that the support provided to the eocyte or the Woese scenario depends on the universal markers included could perhaps explain why several authors keep recovering the overall same eocyte tree in their analyses. They indeed often use datasets containing FES and/or lacking some of the proteins that gave strong support to the Woese’s tree in our analysis, such as the two RNA polymerase subunits or some ribosomal proteins [18,34,58,59]. Some of these authors justified the presence of FES in their dataset by arguing that taxon sampling should be as broad as possible to break up long branches and to minimize LBA [60]. However, simulation analyses have shown that even Bayesian methods with recent models cannot correct strong LBA when the outgroup sequences are too divergent [9]. The addition of taxa that break up long branches is valid as long as the added taxa are not themselves FES with long branches and/or unknown taxonomic affiliation, as it is the case for archaea such as *M*. *kandleri*, Nanoarchaea or *Ca*. K. cryptophylum.

It is often argued that probabilistic methods that model sequence heterogeneity in a Bayesian framework are essential to recover eocyte trees [60,61]. Our result shows that it is not the case since both ML and Bayesian analyses recovered the eocyte tree with the concatenation of the 36 protein datasets. Notably, we notice that in that case, the position of the FES *M*. *kandleri* is not correct in the Bayesian tree published by Spang *et al*. (at the base of the Archaea) whereas it is correct in the ML tree (sister group to Methanobacteriales). We suspect that the Bayesian analysis could be more sensitive to the presence of FES and possible artefacts when outgroup have long branches, in agreement with simulation data recently reported by Gouy and colleagues [9].

In opposition with the general assumption that most universal proteins have a congruent evolutionary history (hence leading to a majority rule to overcome the impact from conflictual evolutionary histories), our results in reanalysing the Loki dataset suggest instead that separate clusters of universal proteins have their own congruent history. The decision over which proteins to include then seems really critical, especially when considering the shortcomings of the incongruence tests [62], comforting the necessity to carefully analyse each individual protein before considering them suitable for concatenation. One of us was confronted to the same situation when analysing the position of *N*. *equitans* [33]. Even though *N*. *equitans* branched between Euryarchaeota and Crenarchaeota in a tree obtained from the concatenation of ribosomal proteins, analysis of individual trees recovered two distinct histories, one supporting the affiliation of *N*. *equitans* to Euryarchaeota (that turned out later to be most likely correct [38]), and another one in which *N*. *equitans* branched within Crenarchaeota, possibly reflecting horizontal gene transfer from its crenarchaeal host, *Ignicoccus hospitalis* [32,33].

Finally, our analyses of the concatenated two largest DNA-dependent RNA polymerase subunits with a new balanced dataset yielded highly similar trees with both ML (LG model) and Bayesian inferences (LG and CAT-GTR models), displaying a topology congruent with a parsimonious scenario of the A subunit split distribution. Interestingly, despite the long bacterial branch, the phylogenies allowed recovering not only the internal consensus phylogeny of Archaea, but also the monophyly of several internal groups that are often difficult to obtain in phylogenetic analyses, such as the Proteobacteria in Bacteria and the Amorpha in Eukaryotes. This supports the robustness of RNA polymerase large subunits in deep phylogeny, and gives additional weight to the Woese scenario. Consequently, this suggests that the same characteristic should be sought in other universal markers to check the scenario they support.

Regarding the position of Lokiarchaea in the Tree of Life, their specific affiliation with Eukaryotes is here supported by a subset of markers that notably comprises many small ribosomal proteins and a long, probably chimeric protein, EF2. Our ML and Bayesian inferences results obtained with the other subset of proteins, and independently with the RNA polymerase subunits, rather indicate that the Lokiarchaeota, the close related Thorarchaeota, and probably the other recently described members of the putative Asgard superphylum, correspond to a new monophyletic archaeal lineage sister group to Euryarchaeota, not to Eukarya. We propose to consider this lineage as a new major archaeal phylum, the Asgardarchaeota. The analyses of genomes obtained from isolated organisms will however be critical to eventually figure out their position without controversy.

Our results question to some extent the validity of eukaryotic specific proteins (ESP) described by Spang *et al*. in lokiarchaeal genomes, suggesting that some could have arisen from contamination. These authors have argued against this possibility because ESP-encoding genes were interspersed in the same contigs with genes encoding proteins with archaeal and/or bacterial affinity [14]. However, this argument is still questionable since our analysis of lokiarchaeal EF2 suggested that insertions of small patches of foreign sequences could likely occur within individual genes. One cannot therefore exclude that genes encoding some ESPs were reconstructed from small patches of eukaryotic sequences that were combined with the homologous archaeal sequences present in the sample; this is especially troubling for the 33 ESPs located on the same set of contigs than EF2 (S6 Table). Altogether, these observations raise major questions concerning the reconstruction of genomes from metagenomic data, especially if a MDA amplification was made during the sequencing process. A troublesome implication of the likely presence of hidden contaminating sequences in the lokiarchaeal genomes is that sequences of artificial hybrid proteins could start to accumulate in public databases. Some biochemists are thus probably already working without awareness on proteins that do not exist in nature.

However, it is also possible that some ESPs genuinely belong to lokiarchaeal genomes, as it has been shown for thaumarchaeal genomes, and were lost during evolution in the other archaeal branches. Indeed, it has been suggested that reductive evolution could be the major direction in archaeal evolution [4,63]. Analysis of further genomes from isolated and cultivated organisms belonging to these new putative archaeal phyla already discovered or yet to be discovered are now prerequisite to definitively settle all questions surrounding their physiology and evolutionary position.

### Concluding remarks

Our analyses demonstrate here that the specific affiliation between Eukarya and Lokiarchaeota previously described is most likely an artefact of genome reconstruction and phylogenetic analyses. Several recent publications based on the lokiarchaeal genomes should thus be revisited and scientists mining these genomes should be particularly cautious. Our work emphasizes the importance to carefully analyze individual protein datasets and trees before drawing any conclusion from phylogenies based on concatenations. It appears especially important to check for the presence of different congruent histories among the universal markers that can be mixed in global analyses, as we observed in the 36 universal proteins used in the Lokiarchaea analysis. Our results indicated that the Lokiarchaea, and probably the other Asgards, correspond to a new monophyletic archaeal lineage sister group to Euryarchaeota, not to Eukarya.

## Methods

### 1-Datasets

#### *Initial dataset*, and *original concatenated alignment*

The initial dataset used for the original Lokiarchaea analysis [14] was kindly provided by Guy L. and Ettema T.J.G., and comprises a maximum of 10 species for both Bacteria and Eukarya and 84 species for Archaea (some species are missing in some proteins; e.g. in the arCOG4064 where there are only 3 eukaryotic species).

The original concatenated alignment, already trimmed was also provided.

#### Curated dataset

To reanalyze the original phylogenies obtained for the different arCOGs, the initial datasets were trimmed of ambiguous sequences that could provide a bias in the phylogenetic analyses (fast-evolving species, FES; sole representatives of their family; sequences not related to specific species; metagenomics reconstructions). Notably:

- Sequences from *Methanopyrus kandleri* were removed because it has been previously shown that its RNA polymerase evolves very rapidly compared to other Archaea, with very long branches and an accumulation of indels [27,32]. As a consequence, *M*. *kandleri* branches between Euryarchaeota and Crenarchaeota in the archaeal RNA polymerase tree, whereas it branches as sister group to *Methanobacteriales* in archaeal trees based on ribosomal proteins [27,32]. This latter position, which has been strongly supported by further analyses [34,38,64], is also coherent with the presence of pseudomurein in the cell wall of *Methanobacteriales* and *M*. *kandleri* [65,66].
- Sequences from *Nanoarchaea* were removed because it has been shown that these parasitic archaea with extremely reduced genomes are fast-evolving species that induce long-branch artefacts [33,34]. In particular, *Nanoarchaeum equitans* is positioned with a long branch between Crenarchaeota and Euryarchaeota in a ribosomal tree [33]. Analysis of individual *N*. *equitans* ribosomal protein phylogenies revealed two distinct histories, similarly to the situation described herein with Lokiarchaeal proteins, and suggested that *N*. *equitans* is an early branching Euryarchaeota, possibly sister group to *Thermococcales* [33]. The specific affiliation of *N*. *equitans* to *Thermococcales* was supported by best-BLAST hits analysis of all *N*. *equitans* proteins, as well as phylogenetic analyses of several informational proteins (topoisomerase VI, reverse gyrase, EFG) and identification of a strong synapomorphy [33].
- Sequences from other nanosized archaea recently detected in metagenomics and single cell analyses (Parvarchaea, Nanohaloarchaea, Micrarchaea, Pacearchaea, Woesearchaea, Aenigmarchaea and Diapherotrites) were removed because they have all been described as fast-evolving species [34]. They often cluster together with *Nanoarchaea* in phylogenetic analyses because of LBA, and group into a putative DPANN (Diapherotrites, Parvarchaeota, Aenigmarchaeota, Nanohaloarchaeota, and Nanoarchaeota) superphylum [36]. These archaea correspond to organisms with small genomes, most of them uncomplete, which are probably symbionts or parasites (their genomes lack essential genes), possibly explaining why they are fast-evolving. Furthermore, their metagenomics origin could be a source of possible contaminations. As an example, S38B Fig shows indels in Kae1 protein within a region that is strictly conserved in all Eukarya and Archaea, except in Nanoarchaea and related nano-sized archaea of the “DPANN superphylum”, and in *M*. *kandleri* where this region is highly variable.
- Sequences from “*Candidatus* Koarchaeum cryptophylum” were removed because this lineage is represented by a single species that display a long branch in phylogenetic trees (possibly fast-evolving), and contains an unusual amino acid bias (supplementary discussion in [14]). We also noticed that the RNA polymerase of “*Ca*. K. cryptophylum” contains long specific insertions reminding those of *M*. *kandleri* RNA polymerases [32]. However, these indels are not homologous to those of *M*. *kandleri*, confirming that these two fast-evolving species are not evolutionarily related (S38A Fig). Notably, *Ca*. K. cryptophylum also exhibits an indel in the region of the Kae1 protein strictly conserved, except in fast-evolving species (S38B Fig).

#### EF2 dataset

We added the amino-acid sequences of four EF2 proteins of Bathyarchaeota to the initial dataset curated of FES (see S5 Table for additional information on taxon sampling).

#### Thorarchaeota dataset

We replaced the lokiarchaeal sequences in the curated dataset by the sequences corresponding to the same proteins from the two most complete thorarchaeal genomes («*Candidatus* Thorarchaeota archaeon » SMTZ1-83 and SMTZ1-45) [30]. Two out of the 36 universal proteins could not be found in any of these genomes: the ribosomal proteins S3 and S4 (arCOG04097 and arCOG04239, respectively). The list and access numbers of the 34 proteins included is presented in supplementary S4 Table.

#### RNA polymerase dataset

The new dataset built to analyze the phylogeny of the largest RNA polymerase subunits was based on datasets used in recent publications [67,68] and on the NCBI taxonomic online platform (http://www.ncbi.nlm.nih.gov/Taxonomy/taxonomyhome.html). The amino-acid sequences were retrieved from the protein database on the NCBI server. For Lokiarchaea, we observed that the initial dataset contained one A-type (Loki 3) and two A’A”-type RNA polymerases (Loki 1 and 2). In addition, we observed only A’A”-type RNA polymerases in the genomes available for the new proposed ‘Thorarchaeota’ phylum, shown to be sister group to Lokiarchaeota [30]. The A-type RNA polymerase is now attributed to an “uncultured organism” in the NCBI database, and only one of the A’A”-type is complete and still annotated as Lokiarchaeum sp. GC14_75 (KKK42229-30). For all these reasons, we decided to use this sequence to represent the Lokiarchaeota in our analyses. We avoided ambiguous species, i.e. sole representatives of their family and fast-evolving species with long branches such as those described before [27,33,34]. When possible, we replaced these fast-evolving species with slow-evolving ones from the same phylum. The final database contained 39 species of each domain.

To find the position of the recently described phyla Bathyarchaeota, Thorarchaeota, Hadesarchaeota and candidate Division MSBL1 archaea (Candidate division “Mediterranean Sea Brine Lakes 1”) [30,53–55], we added 3, 2, 3 and 2 sequences of each respectively in the dataset (see S5 Table for more information). In parallel, in order to position the recently proposed Asgard superphylum, we added sequences from Thorarchaeota, Heimdallarchaeota and Odinarchaeota (see S5 Table).

### 2-Phylogenetic analyses

#### Indels analysis

Lokiarchaeal indels were detected after alignment of the initial dataset. The screening of similar indels in other species was made with a BlastP search against the NCBI non-redundant sequence database using the insertions and their surrounding 40-amino-acids regions, corresponding to strong anchors. The alignments presented in the figures (S13–S16 Figs) were done on these restricted regions. The presentation of the insertions conservation is visualized with SeaView [69]. The presence of indels was also checked by aligning the sequences with PRANK, a probability alignment software [70].

#### Alignments and trimming

Each alignment used for phylogenetic analyses was performed using MAFFT v7 with default settings [71] and trimmed with BMGE [2] with a BLOSUM30 matrix.

#### Maximum likelihood trees

PhyML v3.1 [72] was used to calculate maximum likelihood (ML) trees with the LG amino-acid substitution model and four categories of evolutionary rates (Γ4). The tree search topology operations were based on the BEST option (both NNI and SPR algorithms). Model choice was determined by the Akaike Information Criterion from ProtTest v3 [73]. Branch robustness was estimated with the nonparametric bootstrap procedure (100 replicates). Considering the long lengths of the potentially very distant sequences of the original concatenated alignment (around 10,000 positions with many FES), the ML phylogenetic trees based on it (Fig 4, and detailed trees in S19, S21, S23 and S24 Figs) were performed with IQ-TREE v1.4.2 (http://www.iqtree.org/) with the LG+F+R10 model as suggested by the model selection [74]. The same software was used to investigate the chimeric organization of EF2 (Fig 3 and S17 and S18 Figs) with the TESTNEW option for model selection, and with both nonparametric bootstrap (100 replicates) and ultrafast bootstrap approximation (1,000 replicates).

#### Tree topology selection

Approximately Unbiased (AU) test [40] was used to assess the statistical support of the individual 36 alignments toward the two main topologies discussed in this article (the Woese and eocyte topology) (S2 Table). IQ-TREE v1.4.2 was used for this purpose, with the parameters suggested in the Advanced tutorial for the tree topology selection. The trees tested to represent the Woese and eocyte scenarios were the one obtained from the concatenation of the 11 Woese’s proteins (topologically identical to the RNA polymerase one) and from the concatenation of the 25 eocyte proteins (topologically identical to the 36 proteins tree), respectively, since the taxa need to match between trees and the alignments. Relative certainty, or uncertainty, in tree selection can also be represented as the confidence set that represents the set of trees that are not rejected by the tests. The confidence set of trees is obtained by collecting trees with Pi > = alpha (here 0.05), and a Pi < alpha denote significant exclusion of the tested tree.

To check if the different individual tree topologies obtained were the result of stochastic variation, we performed an additional AU test using PhyML v3.1 and Consel v0.2 [75] (S3 Table). Since the taxa in the alignments need to match the leaves in the trees, only the alignments with relatively similar taxon composition were selected; removing *Borrelia burgdorferi*, *Fervidococcus fontis*, and Loki 2 from all the alignments allowed having 27 of them with identical taxon composition. These were re-aligned, trimmed and their ML trees were reconstructed with the same approaches as described in Methods. Among this new set of trees, 7 and 6 trees that were previously Woese and eocyte trees respectively, still had similar topologies. The other trees, previously low supported, had different eocyte topologies (including 3 that were previously Woese trees). The parameters for the AU test were the same as described above.

#### Bayesian inference

We performed Bayesian inference phylogenies with PhyloBayes v3.3 [76] with the CAT-GTR model and a gamma distribution with four categories of evolutionary rates on the concatenated alignments of the 11 Woese proteins, the 6 AU-relevant Woese proteins, the 25 eocyte proteins (and 24, i.e. without EF2), the 11 AU-relevant eocyte proteins (and 10, i.e. without EF2), the 19 proteins without statistical support in AU test, and all the markers with or without EF2. Four chains were run in parallel, and convergence was checked daily, between every combination of two independent chains (with the first 25% of trees removed as burn-in). Despite extensive computational time and resources, no stationary convergence was observed except for the AU-relevant concatenated protein alignments (6 Woese and 11/10 eocyte proteins). This could be due to an overfit of the model.

Bayesian inference phylogenies were also performed with the same software on the concatenated alignments of the two largest RNA polymerase subunits with both the LG and the CAT-GTR models and a gamma distribution with four categories of evolutionary rates. In both cases, two independent chains were run until they reached convergence with a maximum difference value <0.1. The first 25% of trees were removed as burn-in.

For the RNA polymerase subunits and the AU-relevant proteins, the consensus trees were obtained by selecting one out of every four trees (S9, S10, S27, S30, S31 and S37 Figs). Bayesian posterior probabilities were calculated to estimate the robustness of each branch.

#### Root

We systematically used Bacteria to root the trees [5].

#### Visualization

The phylogenetic trees were analyzed using FigTree software (http://tree.bio.ed.ac.uk/software/figtree/), and iTOL [77].

### 3-Genome quality assessment

The completeness, contamination and heterogeneity of Lokiarchaeon 1 genome were estimated using lineage marker genes with CheckM v1.0.6 [41] and Anvi’o v2.0.2 [42] with standard parameters. For Anvi’o, the markers chosen were those described by Rinke and colleagues [36].

The results obtained with Anvi’o and CheckM indicate that the Lokiarchaeum genome is a chimera of related strains and contaminated sequences (see S12 Fig). An analysis with Anvi’o of the different Loki 1 contigs suggested, by hierarchical clustering based on their tetra-nucleotide sequence composition and their differential reads coverage across the different sequencing runs (SRR1555743, SRR1555748, SRR1555750), that the Loki 1 genome present in the NCBI database (Lokiarchaeum sp. GC14_75) can be divided into separated sets of contigs (S12B Fig). The observed heterogeneity could not be only due to gene duplication because most duplicated markers observed are not located within the same contig. As shown in S12B Fig, we observed that the number of redondant markers detected by Anvi’o increased with the addition of new sets, meaning that similar marker genes are located within different contigs. This observation reflects the fact that the Loki 1 genome was formed by the accretion or assembly of at least two related lokiarchaeum genomes.

This can be illustrated in the case of the RNA polymerase B genes. The Loki 1 genome contains two complete RNA polymerase B genes located on two separate contigs that are located in two separated sets of contigs in S12 Fig, the first one in the set 2 (JYIM01000268) and the second one in the set 4 (JYIM01000029). The Loki castle metagenome assembly contains 4 contigs encoding a lokiarchaeal closely related RNA polymerase B gene (%identity > = 94% on 98% with the Loki 1 protein LAZR01000733-LAZR01000946-LAZR01003597-LAZR01002170). The comparison of the two contigs from the Loki 1 genome to the related contigs of the Loki Castle metagenome is shown S39 Fig. The gene content and the syntheny conservation among these contigs were visualized by tBLASTx approaches in the Easyfig 2.1 program [78]. This comparative analysis revealed at least two subpopulations with different versions of this contig (S39 Fig), with one containing an additional insertion of 5 genes located between a duplicated gene of unknown function. The presence of these two subpopulations was also confirmed by the analysis of the pair-end reads (S39B Fig). The presence of closely related strains in the Loki castle metagenome can thus explain why we observed a high number of single nucleotide variants in the read mapping on the Loki 1 genome. All theses results can be explained by the observation of Spang *et al*. who reported that Lokiarchaeum was the only clade for which four to six distinct but closely related strains were present in the MDA amplified sample [14].

The quality of Loki 2 and Loki 3 genomes, corresponding to two low-abundant distinct DSAG-related lineages obtained from MDA amplified sample (GC content of 32.8% and 29.9%, respectively), could not be verified, at the time of the Lokiarchaeum (Loki 1) genome publication. Indeed, for these two lineages, only 21 and 34 coding sequences (CDS) were available on the NCBI database (S4 Table). From the 57 Gbp produced from the MDA of the Loki Castle sample, only 226 Mbp were available on the NCBI database (SRA access: SRX684860). We looked with Anvi’o for the reads coverage onto the metagenome assembly, the genome of Loki 1, and the CDS of Loki 2 and Loki 3, using read mapping with Bowtie 2 [79], and BlastN search against the non-amplified and the MDA amplified reads from the SRA databases (SRX684860 and SRX684858, respectively). This showed that most of the 226 Mbp available reads correspond to the MDA amplified reads that map on the Lokiarchaeum genome (Loki 1).

### 4-Proteome analysis

The Best BLASTP hit was made for all Loki 1 proteins against the NCBI Reference Sequence Database (available the 4 August 2015 in the Pasteur Server at http://mobyle.pasteur.fr), with 0.001 as limiting expect value (as in [14]). The E-utilities Application Programming Interface (https://www.ncbi.nlm.nih.gov/home/tools.shtml) was used to access the NCBI databases: Taxonomy IDs for all best hits were extracted using efetch and xtract functions on the protein database. Then the taxonomic lineages of best-hit proteins were extracted from the taxonomy NCBI database using efetch and xtract functions.

## Supporting information

S1 Fig Maximum likelihood (ML) single protein trees for the 36 genes included in the concatenated alignment of Spang *et al*. 2015. For all trees, the scale-bar indicates the average number of substitutions per site, and values at nodes represent support calculated by nonparametric bootstrap (out of 100). Bacterial and eukaryotic sequences are indicated in red and blue respectively, while Loki sequences are indicated in green. In each tree, a red arrow indicates the lokiarchaeal sequence corresponding to Lokiarchaeon 1.
(PDF)

S2 Fig ML single protein trees of the 36 genes with the curated datasets. For all trees, the scale-bar indicates the average number of substitutions per site, and values at nodes represent support calculated by nonparametric bootstrap (out of 100). Bacterial and eukaryotic sequences are indicated in red and blue respectively, while lokiarchaeal sequences are indicated in green. In each tree, a red arrow indicates the lokiarchaeal sequence corresponding to Lokiarchaeon 1. The trees corresponding to the arCOG00412, arCOG01183, and arCOG01559 display more colours as they are representative of the different patterns observed among the trees: the lokiarchaeal sequences within Archaea, the lokiarchaeal sequences at different positions with one being sister group to Eukaryotes, and all the lokiarchaeal sequences sister group to Eukarya, respectively. In these trees, Crenarchaeota, Euryarchaeota, and Thaumarchaeota are indicated in orange, green, and pink, respectively.
(PDF)

S3 Fig ML phylogenetic tree of the concatenation of the 11 Woese’s proteins from the curated datasets (3,499 positions). In this tree, bacterial and eukaryotic sequences are indicated in red and blue, respectively. For Archaea, Thaumarchaeota and Aigarchaeota are indicated in pink, Crenarchaeota in orange and Euryarchaeota in olive-green. The Lokiarchaeota are indicated in light-green. The scale-bar represents the average number of substitutions per site. Values at nodes represent support calculated by nonparametric bootstrap (out of 100).
(PDF)

S4 Fig ML phylogenetic tree of the concatenation of the 25 eocyte proteins from the curated datasets (4,868 positions). In this tree, bacterial and eukaryotic sequences are indicated in red and blue, respectively. For Archaea, Thaumarchaeota and Aigarchaeota are indicated in pink, Crenarchaeota in orange and Euryarchaeota in olive-green. The Lokiarchaeota are indicated in light-green. The scale-bar represents the average number of substitutions per site. Values at nodes represent support calculated by nonparametric bootstrap (out of 100).
(PDF)

S5 Fig ML phylogenetic tree of the concatenation of 8 Woese’s proteins from the curated datasets (1,582 positions). The 8 proteins correspond to all the Woese’s proteins minus the RNA polymerase subunits A’/A” and B. In this tree, bacterial and eukaryotic sequences are indicated in red and blue, respectively. For Archaea, Thaumarchaeota and Aigarchaeota are indicated in pink, Crenarchaeota in orange and Euryarchaeota in olive-green. The Lokiarchaeota are indicated in light-green. The scale-bar represents the average number of substitutions per site. Values at nodes represent support calculated by nonparametric bootstrap (out of 100).
(PDF)

S6 Fig ML phylogenetic tree of the concatenation of 24 eocyte proteins from the curated datasets (4,225 positions). The 24 proteins correspond to all the eocyte proteins minus EF2. In this tree, bacterial and eukaryotic sequences are indicated in red and blue, respectively. For Archaea, Thaumarchaeota and Aigarchaeota are indicated in pink, Crenarchaeota in orange and Euryarchaeota in olive-green. The Lokiarchaeota are indicated in light-green. The scale-bar represents the average number of substitutions per site. Values at nodes represent support calculated by nonparametric bootstrap (out of 100).
(PDF)

S7 Fig ML phylogenetic tree of the concatenation of the 6 AU-relevant Woese’s proteins from the curated datasets (1,857 positions). In this tree, bacterial and eukaryotic sequences are indicated in red and blue, respectively. For Archaea, Thaumarchaeota and Aigarchaeota are indicated in pink, Crenarchaeota in orange and Euryarchaeota in olive-green. The Lokiarchaeota are indicated in light-green. The scale-bar represents the average number of substitutions per site. Values at nodes represent support calculated by nonparametric bootstrap (out of 100).
(PDF)

S8 Fig ML phylogenetic tree of the concatenation of the 11 AU-relevant eocyte proteins from the curated datasets (2,750 positions). In this tree, bacterial and eukaryotic sequences are indicated in red and blue, respectively. For Archaea, Thaumarchaeota and Aigarchaeota are indicated in pink, Crenarchaeota in orange and Euryarchaeota in olive-green. The Lokiarchaeota are indicated in light-green. The scale-bar represents the average number of substitutions per site. Values at nodes represent support calculated by nonparametric bootstrap (out of 100).
(PDF)

S9 Fig Bayesian inference phylogeny of the concatenation of the 6 AU-relevant Woese’s proteins from the curated datasets (1,857 positions). In this tree, bacterial and eukaryotic sequences are indicated in red and blue, respectively. For Archaea, Thaumarchaeota and Aigarchaeota are indicated in pink, Crenarchaeota in orange and Euryarchaeota in olive-green. The Lokiarchaeota are indicated in light-green. Values at nodes indicate the Bayesian posterior probabilities. The scale-bar represents the average number of substitutions per site.
(PDF)

S10 Fig Bayesian inference phylogeny of the concatenation of the 11 AU-relevant eocyte proteins from the curated datasets (2,750 positions). In this tree, bacterial and eukaryotic sequences are indicated in red and blue, respectively. For Archaea, Thaumarchaeota and Aigarchaeota are indicated in pink, Crenarchaeota in orange and Euryarchaeota in olive-green. The Lokiarchaeota are indicated in light-green. Values at nodes indicate the Bayesian posterior probabilities. The scale-bar represents the average number of substitutions per site.
(PDF)

S11 Fig ML phylogenetic tree of the concatenation of the 19 proteins from the curated datasets that are not significant in AU test (3,760 positions). In this tree, bacterial and eukaryotic sequences are indicated in red and blue, respectively. For Archaea, Thaumarchaeota and Aigarchaeota are indicated in pink, Crenarchaeota in orange and Euryarchaeota in olive-green. The Lokiarchaeota are indicated in light-green. The scale-bar represents the average number of substitutions per site. Values at nodes represent support calculated by nonparametric bootstrap (out of 100).
(PDF)

S12 Fig The Loki 1 genome quality. **a.** Table summarizing the results obtained with CheckM and Anvi’o on Loki 1 genome quality. **b.** Graphical view of the Anvi’o interactive display of the Lokiarchaeum genome (Loki 1). The clustering dendrogram in the center displays the hierarchical contigs clustering based on their tetra-nucleotide sequence composition and their differential reads coverage across the different sequencing runs. Each of the 513 tips represents a contig or a split contig as Anvi’o splits contigs too long. These are still located together and noticed by a grey bar on the upper layer (“parent” layer). The length and GC layers show the relative length and GC-content of a contig. The additional layers represent the relative abundance (coverage) of each contig in the different sequencing runs (SRR1555743, SRR1555748, SRR1555750). The green stars indicate the position of the two contigs encoding the RNA polymerase subunits A and B genes used in the different concatenations. The orange star indicate the position of the contig encoding EF2. The table on the bottom gives additional information regarding the sets suggested by this analysis, notably their length and composition, and the results of different combinations of sets.
(PDF)

S13 Fig Alignment of the N-terminal lokiarchaeal EF2 insertion (A1, A2, A3). Alignment of the region corresponding to the insertion A1, A2 and A3 in lokiarchaeal EF2 sequences, with archaeal EF2 sequences and eukaryotic Ria sequences (EF2 paralog), and with Ria sequences from a subgroup of fungi (bottom alignment). Organisms’ names corresponding to Lokiarchaea/Thorarchaea, Archaea, and Eukarya are respectively indicated in brown, green, and blue.
(PDF)

S14 Fig Alignment of insertion B3 of the Loki 3 EF2 protein. Alignment of the region corresponding to the B3 insertion (located in positions 268 to 323 of the Loki 3 EF2 protein) with archaeal EF2 sequences and eukaryotic Ria sequences (EF2 paralog). Organisms’ names corresponding to Lokiarchaea/Thorarchaea, Archaea, and Eukarya are respectively indicated in brown, green, and blue.
(PDF)

S15 Fig Alignment of insertion C3 of the Loki 3 EF2 protein. Alignment of the region corresponding to the C3 insertion (located in positions 373 to 406 of the Loki 3 EF2 protein) with archaeal and eukaryotic EF2 sequences and eukaryotic Ria sequences (EF2 paralog). Organisms’ names corresponding to Bacteria, Lokiarchaea/Thorarchaea, Archaea, and Eukarya are respectively indicated in red, brown, green, and blue.
(PDF)

S16 Fig Alignment of insertion D3 of the Loki 3 EF2 protein. Alignment of the region corresponding to the D3 insertion (located in positions 780 to 818 of the Loki 3 EF2 protein) with archaeal and eukaryotic EF2 sequences and eukaryotic snu5 sequences (EF2 paralog). Sequences corresponding to Bacteria, Lokiarchaea/Thorarchaea, Archaea, and Eukarya are respectively indicated in red, brown, green, and blue.
(PDF)

S17 Fig ML phylogenetic trees of the Elongation Factor 2 (EF2) after inclusion of bathyarchaeal sequences (ultrafast bootstrap approximation). **a.** ML phylogeny obtained with the N-terminal section of EF2 (232 sites). **b.** ML phylogeny obtained with the C-terminal section of the protein (394 sites). **c.** ML phylogeny obtained with the entire EF2 protein (626 sites). In these trees, bacterial and eukaryotic sequences are indicated in red and blue, respectively. For Archaea, Thaumarchaeota and Aigarchaeota are indicated in pink, Crenarchaeota in orange and Euryarchaeota in olive-green. The Lokiarchaea are indicated in light-green. The scale-bar represents the average number of substitutions per site. Values at nodes represent support calculated by ultrafast bootstrap approximation (out of 100; 1,000 replicates).
(PDF)

S18 Fig ML phylogenetic trees of the Elongation Factor 2 (EF2) after inclusion of bathyarchaeal sequences (nonparametric bootstrap). **a.** ML phylogeny obtained with the N-terminal section of EF2 (232 sites). **b.** ML phylogeny obtained with the C-terminal section of the protein (394 sites). **c.** ML phylogeny obtained with the entire EF2 protein (626 sites). In these trees, bacterial and eukaryotic sequences are indicated in red and blue, respectively. For Archaea, Thaumarchaeota and Aigarchaeota are indicated in pink, Crenarchaeota in orange and Euryarchaeota in olive-green. The Lokiarchaea are indicated in light-green. The scale-bar represents the average number of substitutions per site. Values at nodes represent support calculated by nonparametric bootstrap (out of 100; 100 replicates).
(PDF)

S19 Fig ML phylogenetic tree of the original concatenated alignment (36 arCOGs; 10,547 positions). In this tree, bacterial and eukaryotic sequences are indicated in red and blue, respectively. For Archaea, Thaumarchaeota and Aigarchaeota are indicated in pink, Crenarchaeota in orange and Euryarchaeota in olive-green. The lokiarchaea are indicated in light-green, and their position is pointed on the figure. The scale-bar represents the average number of substitutions per site. Values at nodes represent support calculated by nonparametric bootstrap (out of 100).
(PDF)

S20 Fig ML phylogenetic tree of the concatenation of the 36 arCOGs from the curated datasets (8,367 positions). In this tree, bacterial and eukaryotic sequences are indicated in red and blue, respectively. For Archaea, Thaumarchaeota and Aigarchaeota are indicated in pink, Crenarchaeota in orange and Euryarchaeota in olive-green. Lokiarchaeal sequences are indicated in light-green. The scale-bar represents the average number of substitutions per site. Values at nodes represent support calculated by nonparametric bootstrap (out of 100).
(PDF)

S21 Fig ML phylogenetic tree of the original concatenated alignment after removal of the EF2 protein (35 arCOGs; 9,831 positions). In this tree, bacterial and eukaryotic sequences are indicated in red and blue, respectively. For Archaea, Thaumarchaeota and Aigarchaeota are indicated in pink, Crenarchaeota in orange and Euryarchaeota in olive-green. Lokiarchaea are indicated in light-green and their position is pointed on the figure. The scale-bar represents the average number of substitutions per site. Values at nodes represent support calculated by nonparametric bootstrap (out of 100).
(PDF)

S22 Fig ML phylogenetic tree of the concatenation of 35 arCOGs from the curated datasets (all markers except EF2; 7,724 positions). In this tree, bacterial and eukaryotic sequences are indicated in red and blue, respectively. For Archaea, Thaumarchaeota and Aigarchaeota are indicated in pink, Crenarchaeota in orange and Euryarchaeota in olive-green. The Lokiarchaeota are indicated in light-green. The scale-bar represents the average number of substitutions per site. Values at nodes represent support calculated by nonparametric bootstrap (out of 100).
(PDF)

S23 Fig ML phylogenetic tree of the original concatenated alignment after removal of Lokiarchaeon 3 sequences (36 arCOGs; 10,547 positions). In this tree, bacterial and eukaryotic sequences are indicated in red and blue, respectively. For Archaea, Thaumarchaeota and Aigarchaeota are indicated in pink, Crenarchaeota in orange and Euryarchaeota in olive-green. The Lokiarchaeota are indicated in light-green. The scale-bar represents the average number of substitutions per site. Values at nodes represent support calculated by nonparametric bootstrap (out of 100).
(PDF)

S24 Fig ML phylogenetic tree of the original concatenated alignment after removal of the Loki 3 EF2 sequence (36 arCOGs; 10,547 positions). In this tree, bacterial and eukaryotic sequences are indicated in red and blue, respectively. For Archaea, Thaumarchaeota and Aigarchaeota are indicated in pink, Crenarchaeota in orange and Euryarchaeota in olive-green. The Lokiarchaeota are indicated in light-green and their position is pointed on the figure. The scale-bar represents the average number of substitutions per site. Values at nodes represent support calculated by nonparametric bootstrap (out of 100).
(PDF)

S25 Fig ML phylogenetic tree of the concatenation of the 36 arCOGs from the curated datasets after removal of Loki 3 EF2 sequence (8,425 positions). In this tree, bacterial and eukaryotic sequences are indicated in red and blue, respectively. For Archaea, Thaumarchaeota and Aigarchaeota are indicated in pink, Crenarchaeota in orange and Euryarchaeota in olive-green. The Lokiarchaeota are indicated in light-green. The scale-bar represents the average number of substitutions per site. Values at nodes represent support calculated by nonparametric bootstrap (out of 100).
(PDF)

S26 Fig ML phylogenetic tree of the concatenation of 10 AU-relevant eocyte proteins (all AU-relevant eocyte proteins minus EF2; 2,107 positions). In this tree, bacterial and eukaryotic sequences are indicated in red and blue, respectively. For Archaea, Thaumarchaeota and Aigarchaeota are indicated in pink, Crenarchaeota in orange and Euryarchaeota in olive-green. The Lokiarchaeota are indicated in light-green. The scale-bar represents the average number of substitutions per site. Values at nodes represent support calculated by nonparametric bootstrap (out of 100).
(PDF)

S27 Fig Bayesian inference phylogeny of the concatenation of 10 AU-relevant eocyte proteins (all AU-relevant eocyte proteins minus EF2; 2,107 positions). In this tree, bacterial and eukaryotic sequences are indicated in red and blue, respectively. For Archaea, Thaumarchaeota and Aigarchaeota are indicated in pink, Crenarchaeota in orange and Euryarchaeota in olive-green. The Lokiarchaeota are indicated in light-green. Values at nodes indicate the Bayesian posterior probabilities. The scale-bar represents the average number of substitutions per site.
(PDF)

S28 Fig ML phylogenetic tree of the concatenation of the 34 arCOGs from the curated datasets present in the two most complete thorarchaeal genomes (8,840 positions). In this tree, bacterial and eukaryotic sequences are indicated in red and blue, respectively. For Archaea, Thaumarchaeota and Aigarchaeota are indicated in pink, Crenarchaeota in orange and Euryarchaeota in olive-green. The Thorarchaea (*Candidatus* Thorarchaeota archaea) are indicated in black. The scale-bar represents the average number of substitutions per site. Values at nodes represent support calculated by nonparametric bootstrap (out of 100).
(PDF)

S29 Fig Alignment of the region corresponding to the split in the RNA polymerase subunit A protein sequence. Organisms’ name corresponding to Bacteria, Lokiarchaea/Thorarchaea, Archaea, and Eukarya are respectively indicated in red, brown, green, and blue.
(PDF)

S30 Fig Bayesian inference phylogeny of the concatenation of the two largest RNA polymerase subunits with LG substitution model (Γ4) on the new dataset. The same number (39) of Archaea (green), Eukaryotes (blue) and Bacteria (red) were selected (1,463 positions; see S5 Table for the dataset). Values at nodes indicate the Bayesian posterior probabilities. The scale-bar represents the average number of substitutions per site.
(PDF)

S31 Fig Bayesian inference phylogeny of the concatenation of the two largest RNA polymerase subunits with CAT-GTR evolution model (Γ4) on the new dataset. The same number (39) of Archaea (green), Eukaryotes (blue) and Bacteria (red) were selected (1,463 positions; see S5 Table for the dataset). Values at nodes indicate the Bayesian posterior probabilities. The scale-bar represents the average number of substitutions per site.
(PDF)

S32 Fig ML phylogeny of the concatenation of the two largest RNA polymerase subunits on the new dataset. The same number (39) of Archaea (green), Eukaryotes (blue) and Bacteria (red) were selected (1,463 positions). Values at nodes indicate support calculated by nonparametric bootstrap (out of 100). The scale-bar represents the average number of substitutions per site.
(PDF)

S33 Fig Positions of Bathyarchaeota, Thorarchaeota, Hadesarchaeota and candidate division MSBL1 archaea based on the concatenation of the two largest RNA polymerase subunits on the new dataset. **a and b.** ML phylogenetic trees of the concatenation of the two largest RNA polymerase subunits, using Bacteria as outgroup (1,670 positions) (**a**) or Eukaryotes (bacterial sequences removed; 2,175 positions) (**b**). Detailed trees in S34 and S35 Figs. Values at nodes indicate support calculated by nonparametric bootstrap (out of 100). The scale-bars represent the average number of substitutions per site.
(PDF)

S34 Fig ML phylogenetic tree of the concatenation of the two largest RNA polymerase subunits with the new dataset after inclusion of Bathyarchaeota, Thorarchaeota, Hadesarchaeota, and candidate division MSBL1 archaea. In this tree, bacterial and eukaryotic sequences are indicated in red and blue, respectively. For Archaea, Thaumarchaeota and Aigarchaeota are indicated in pink, Crenarchaeota in orange and Euryarchaeota in olive-green. The scale-bar represents the average number of substitutions per site. Values at nodes represent support calculated by nonparametric bootstrap (out of 100).
(PDF)

S35 Fig ML phylogenetic tree of the concatenation of the two largest RNA polymerase subunits with the new dataset after inclusion of Bathyarchaeota, Thorarchaeota, Hadesarchaeota, and candidate division MSBL1 archaea, and removal of bacterial sequences. In this tree, eukaryotic sequences are indicated in blue, and are used as outgroup. For Archaea, Thaumarchaeota and Aigarchaeota are indicated in pink, Crenarchaeota in orange and Euryarchaeota in olive-green. The scale-bar represents the average number of substitutions per site. Values at nodes represent support calculated by nonparametric bootstrap (out of 100).
(PDF)

S36 Fig ML phylogenetic tree of the concatenation of the two largest RNA polymerase subunits with the new dataset after inclusion of Asgard archaea. In this tree, bacterial and eukaryotic sequences are indicated in red and blue, respectively. For Archaea, Thaumarchaeota and Aigarchaeota are indicated in pink, Crenarchaeota in orange and Euryarchaeota in olive-green. The scale-bar represents the average number of substitutions per site. Values at nodes represent support calculated by nonparametric bootstrap (out of 100).
(PDF)

S37 Fig Bayesian inference phylogeny of the concatenation of the two largest RNA polymerase subunits on the new dataset after inclusion of Asgard archaea with CAT-GTR evolution model (Γ4). In this tree, bacterial and eukaryotic sequences are indicated in red and blue, respectively. For Archaea, Thaumarchaeota and Aigarchaeota are indicated in pink, Crenarchaeota in orange and Euryarchaeota in olive-green. Values at nodes indicate the Bayesian posterior probabilities. The scale-bar represents the average number of substitutions per site.
(PDF)

S38 Fig Alignments of indels of *Candidatus* Korarchaeum cryptofilum and *Methanopyrus kandleri*. a. Alignments of the regions corresponding to two indels located on the RNA polymerase subunit A (on the left, starting position around 750, on the right around 1200). b. Alignment of the region corresponding to the indel located at the end of the Kae1 protein, with archaeal and eukaryotic sequences. Organisms’ names corresponding to Archaea and Eukaryotes are indicated in black and blue, respectively. The archaea presenting an indel are indicated in pink.
(PDF)

S39 Fig Comparison of the lokiarchaeal contigs encoding the RNA polymerase subunit B gene in the Loki Castle metagenome assembly and in the Loki 1 genome. The gene encoding the RNA polymerase subunit is colored in light green. **a.** Comparison of the two Loki 1 contigs encoding the RNA polymerase B gene, and to their related contigs in the metagenome assembly. The names of the contigs corresponding to the metagenome are indicated in purple and those corresponding to the Loki 1 genome are indicated in pink or green based on their position (Set 2 and Set 4, respectively) in the S12 Fig on the analysis of the quality of the Loki 1 genome. The identity percentage between the contigs by tBLASTx approaches is also indicated. **b.** The two pairs of graphs correspond to reads coverage of Loki 1 contig 29 and Loki Metagenome contig 946, across the SRR1555743 and SRR1555748 sequencing runs (abbreviated SRR743 and SRR748, and in light blue and mauve, respectively). In theses graphs, the grey bars represent the base frequencies of small nucleotide variants (SNVs) observed in the contigs. The comparison of theses two contigs showed an insertion of five putative genes in the loki 1 contig 29 compared to the loki metagenome contig 946. The values indicated over the red arrows correspond to the total number of mapped pair-end reads (SRR1555743 and SRR1555748 runs combined) that support the absence on the five putative genes insertion and those that support both sides of the insertion.
(PDF)

S1 Dataset Concatenated alignments. Concatenated alignments used in this study: the 6 AU-relevant Woese’s proteins (phylip format), the 25 AU-relevant eocyte proteins (phylip format), the 36 universal markers (phylip format) used for the Lokiarchaea analyses, the 34 universal markers (phylip format) used for the Thorarchaea analysis, and the two DNA-dependent RNA polymerase large subunits with the new dataset (fasta format) and with the Asgards archaea (phylip format). The positions of each individual markers within the concantenations are indicated within a text file. The alignment of EF2 performed with PRANK is included (fasta format).
(ZIP)

S1 Supporting information Detailed results of the Loki 1 genome quality analysis with Anvi’o. The data related to the Loki 1 genome quality analysis can be interactively browsed through the included index.html file with an internet browser software.
(ZIP)

S1 Table Comparative analysis of the 36 universal proteins phylogenetic trees obtained with the initial dataset (ID) and the FES-curated dataset (CD). (PDF)

S2 Table Results of Approximately Unbiased test with single protein alignments on the 11 or 25 proteins tree topology selection. (PDF)

S3 Table Results of Approximately Unbiased test with 27 single protein alignments on the different single protein topologies. (PDF)

S4 Table List of accession numbers of the Lokiarchaeal and Thorarchaeal proteins included in the concatenation-based analyses. (PDF)

S5 Table List of the species sampled for the different phylogenetic analyses in this study. (PDF)

S6 Table Number of ESPs located in the different sets of contigs suggested from the genome quality analysis. (PDF)
