# Supplementary material for: Lokiarchaea are close relatives of Euryarchaeota, not bridging the gap between prokaryotes and eukaryotes
Source: PLoS Genet. 2017 Jun 12;13(6):e1006810. doi: 10.1371/journal.pgen.1006810 (PMC5484517; doi:10.1371/journal.pgen.1006810)
Supplement: S1 Fig — For all trees, the scale-bar indicates the average number of substitutions per site, and values at nodes represent support calculated by nonparametric bootstrap (out of 100). Bacterial and eukaryotic sequences are indicated in red and blue respectively, while Loki sequences are indicated in green. In each tree, a red arrow indicates the lokiarchaeal sequence corresponding to Lokiarchaeon 1. (PDF) [file pgen.1006810.s001.pdf]

S1 Fig – Maximum likelihood (ML) single protein trees for the 36 genes included in the concatenated alignment of Spang *et al.* 2015.

For all trees, the scale-bar indicates the average number of substitutions per site, and values at nodes represent support calculated by nonparametric bootstrap (out of 100). Bacterial and eukaryotic sequences are indicated in red and blue respectively, while Loki sequences are indicated in green. In each tree, a red arrow indicates the lokiarchaeal sequence corresponding to Lokiarchaeon 1.

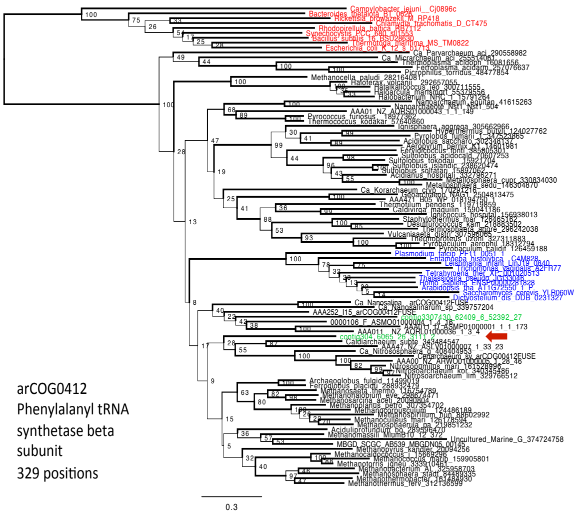

arCOG0412
Phenylalanyl tRNA synthetase beta subunit
329 positions

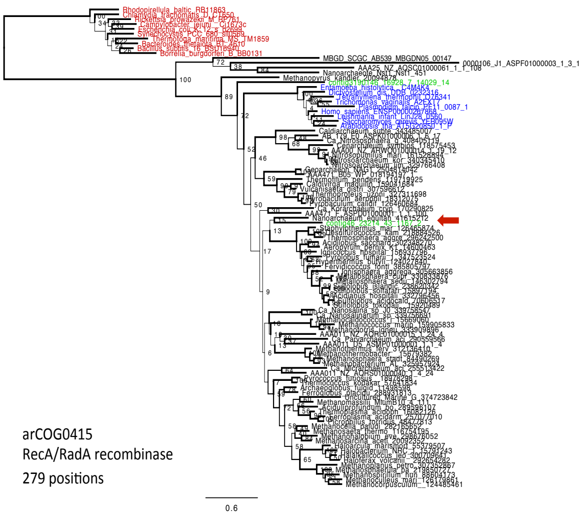

arCOG0415
RecA/RadA recombinase
279 positions

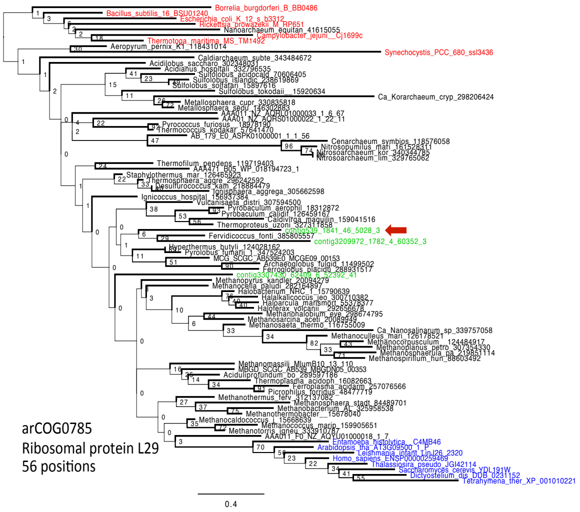

arCOG0785
Ribosomal protein L29
56 positions

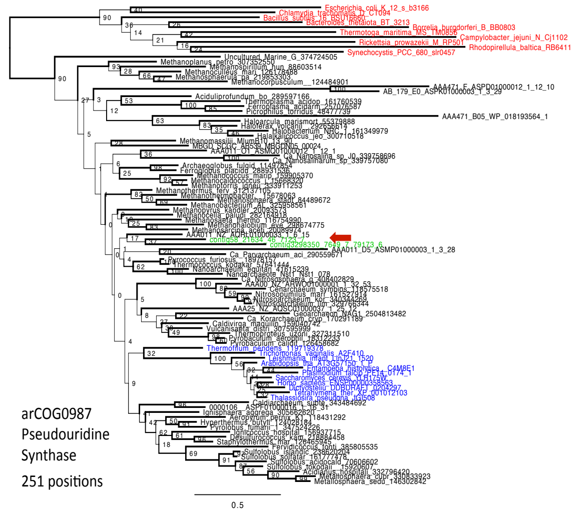

arCOG0987
Pseudouridine Synthase
251 positions

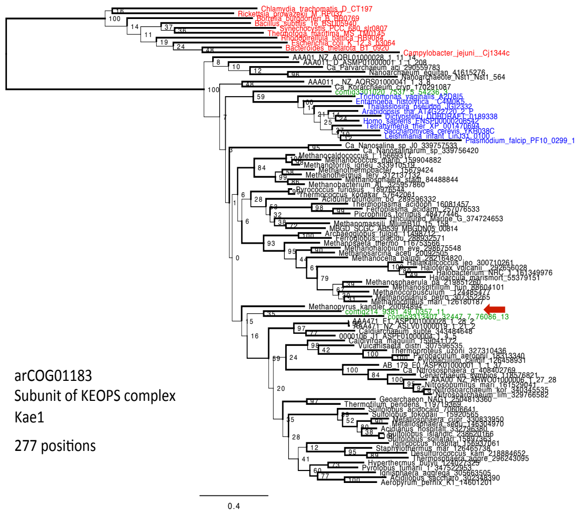

arCOG01183
Subunit of KEOPS complex Kae1
277 positions

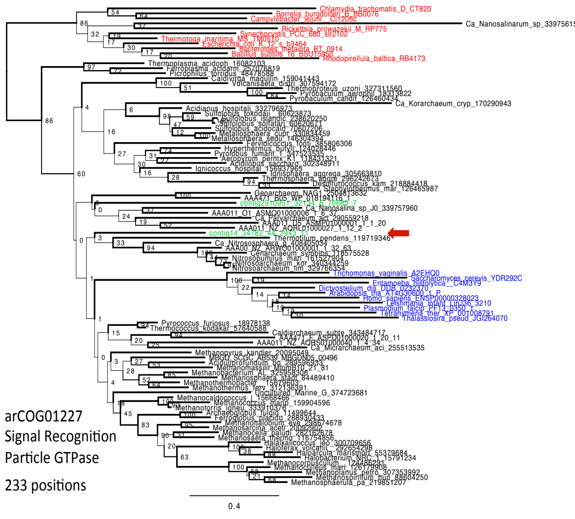

arCOG01227
Signal Recognition Particle GTPase
233 positions

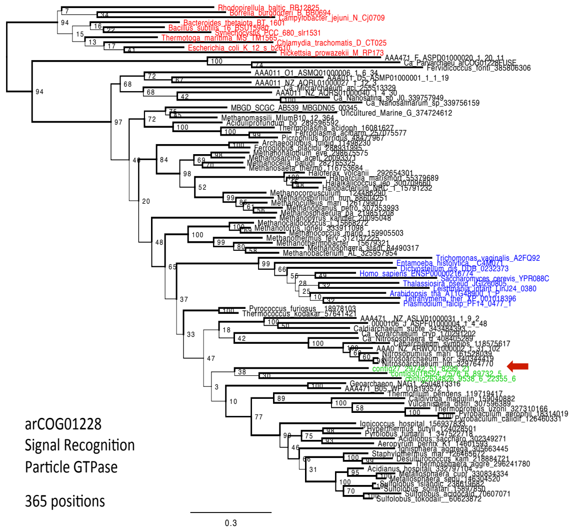

arCOG01228
Signal Recognition
Particle GTPase

365 positions

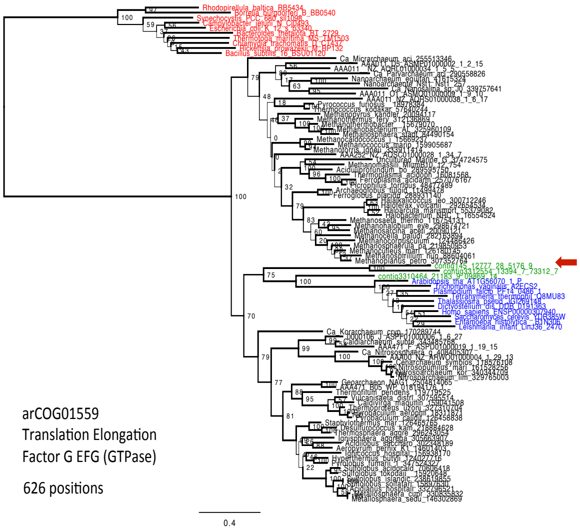

arCOG01559
Translation Elongation
Factor G EFG (GTPase)

626 positions

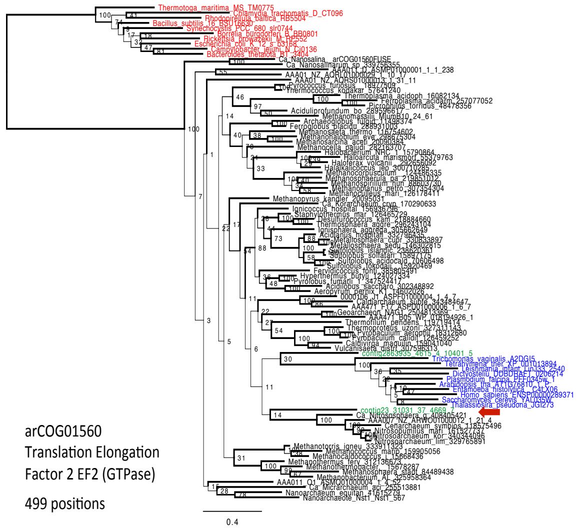

arCOG01560
Translation Elongation
Factor 2 EF2 (GTPase)

499 positions

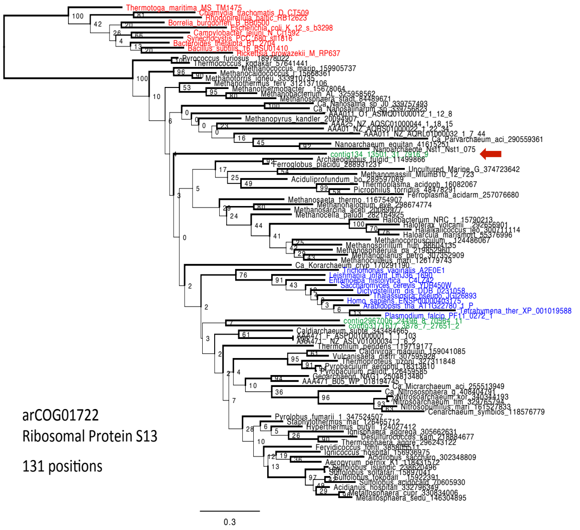

arCOG01722
Ribosomal Protein S13

131 positions

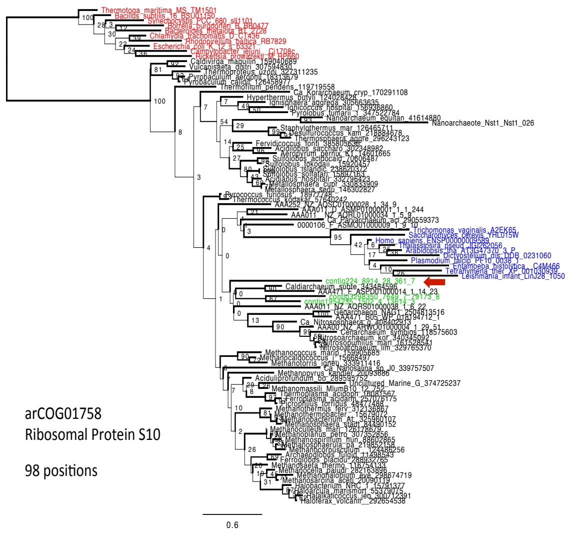

arCOG01758
Ribosomal Protein S10

98 positions

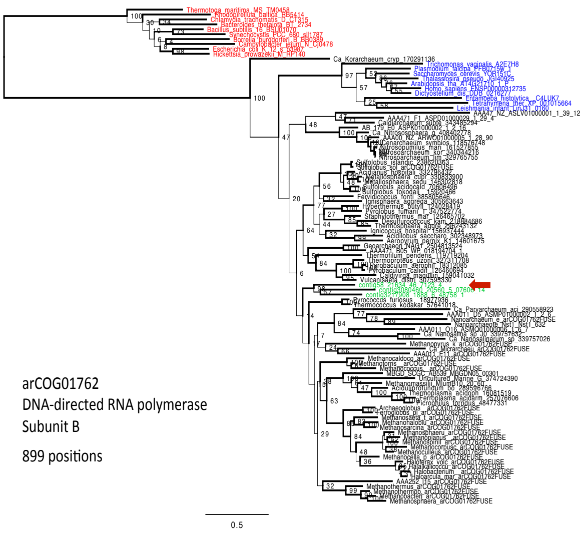

arCOG01762
DNA-directed RNA polymerase
Subunit B

899 positions

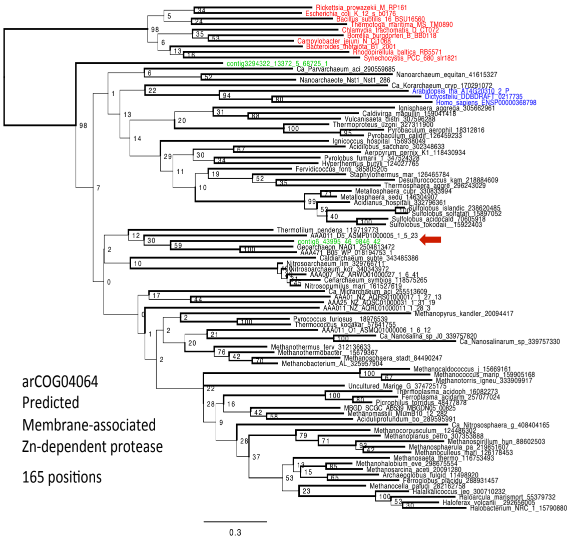

arCOG04064
Predicted
Membrane-associated
Zn-dependent protease

165 positions

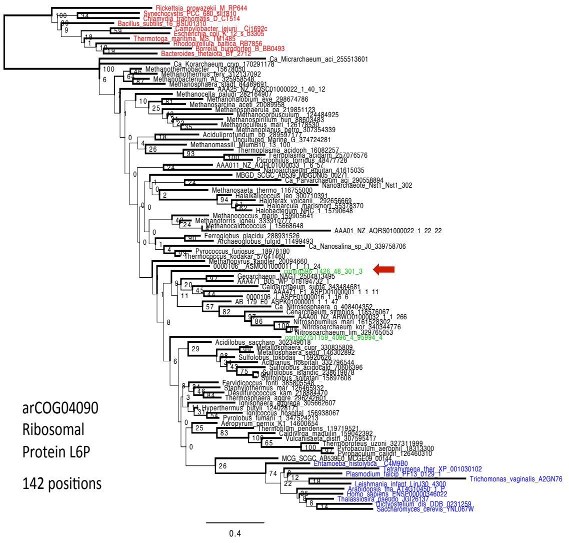

arCOG04090
Ribosomal
Protein L6P

142 positions

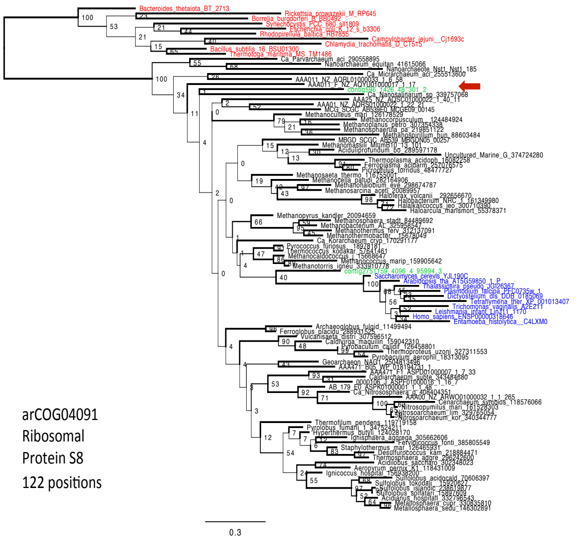

arCOG04091
Ribosomal
Protein S8

122 positions

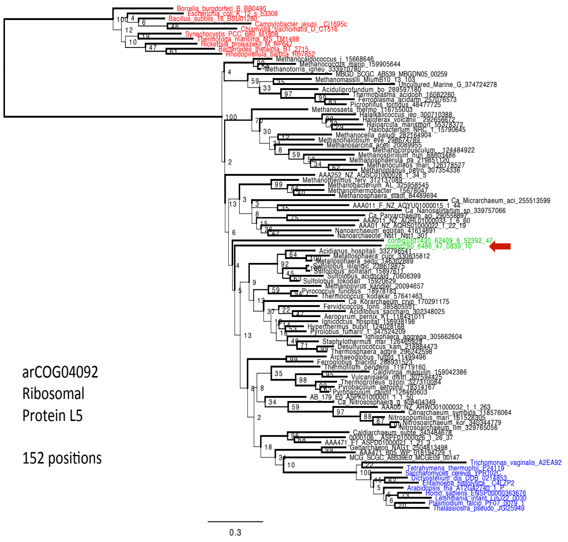

arCOG04092
Ribosomal
Protein L5

152 positions

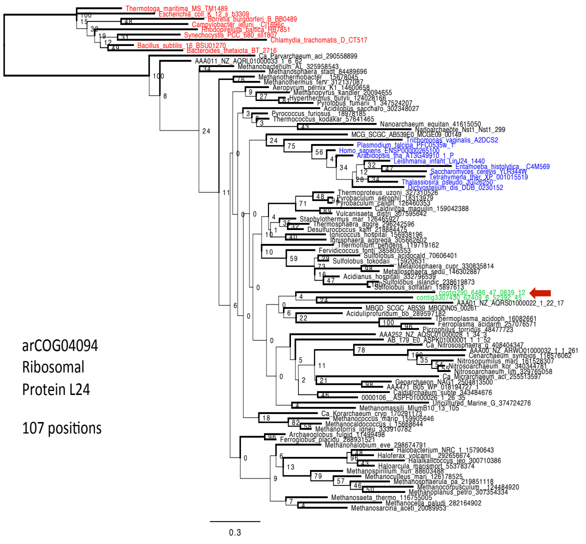

arCOG04094
Ribosomal
Protein L24

107 positions

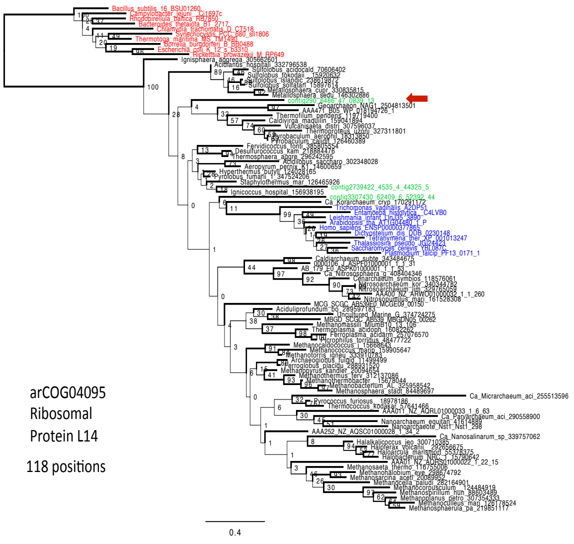

arCOG04095
Ribosomal
Protein L14

118 positions

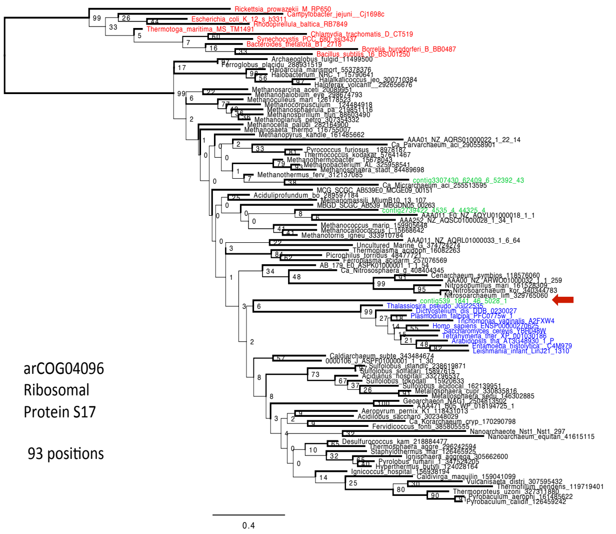

arCOG04096
Ribosomal
Protein S17

93 positions

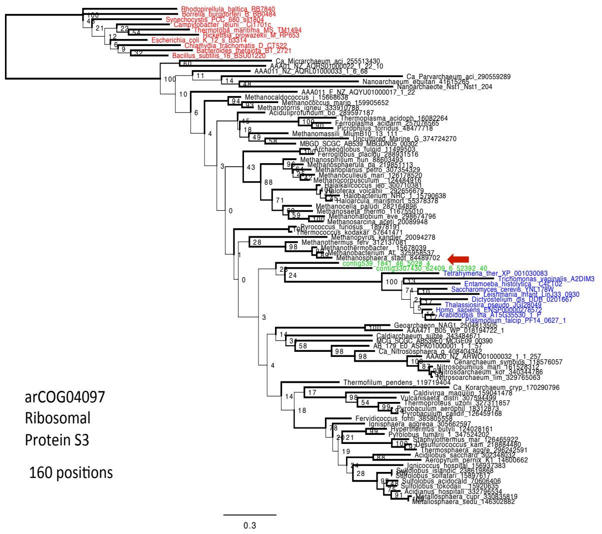

arCOG04097
Ribosomal
Protein S3

160 positions

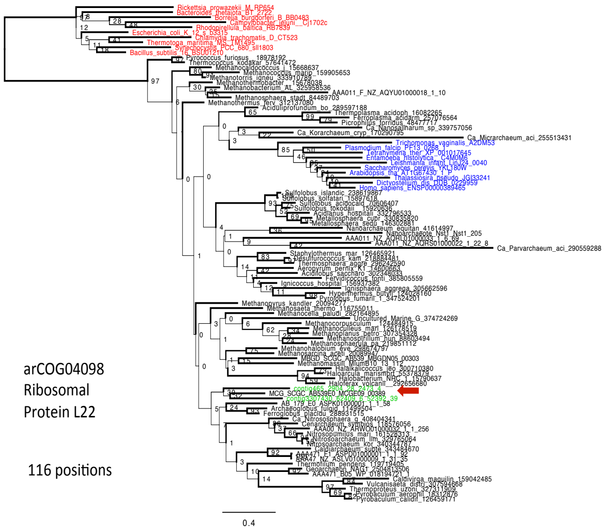

arCOG04098
Ribosomal
Protein L22

116 positions

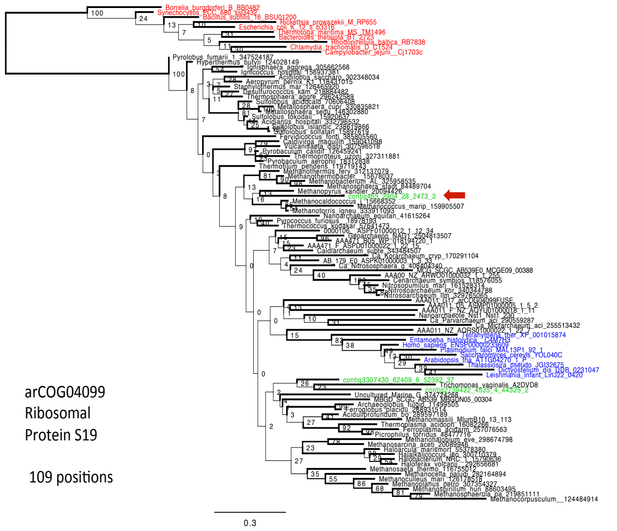

arCOG04099
Ribosomal
Protein S19

109 positions

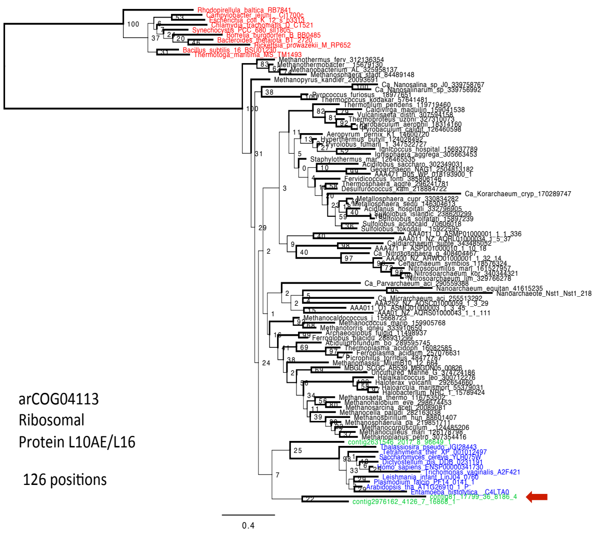

arCOG04113
Ribosomal
Protein L10AE/L16

126 positions

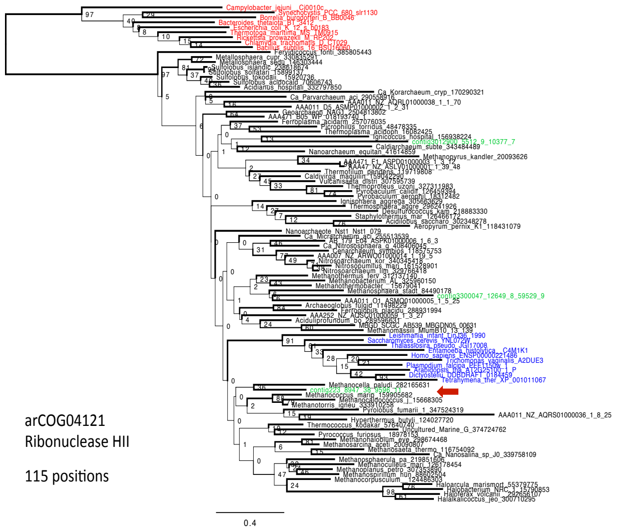

arCOG04121
Ribonuclease HII

115 positions

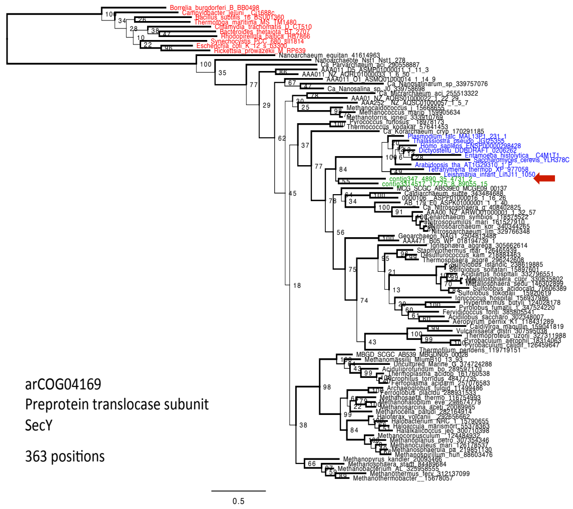
arCOG04169
Preprotein translocase subunit
SecY
363 positions
0.5

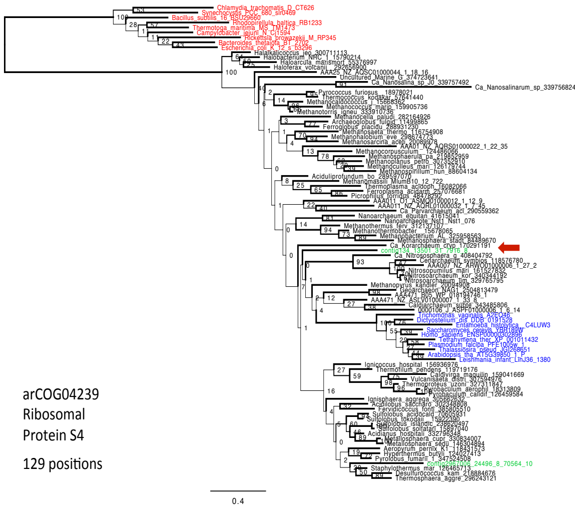
arCOG04239
Ribosomal
Protein S4
129 positions
0.4

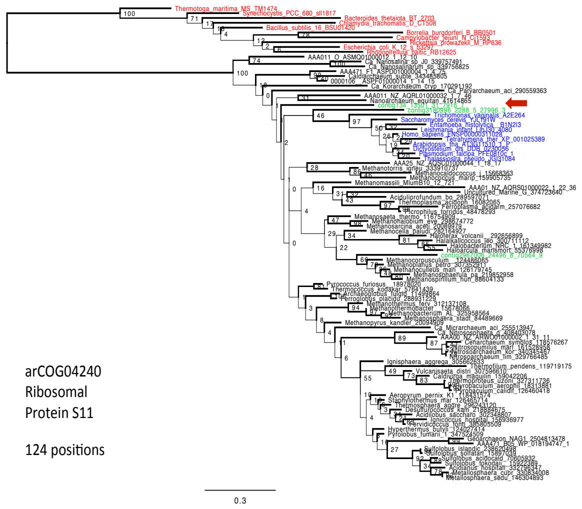
arCOG04240
Ribosomal
Protein S11
124 positions
0.3

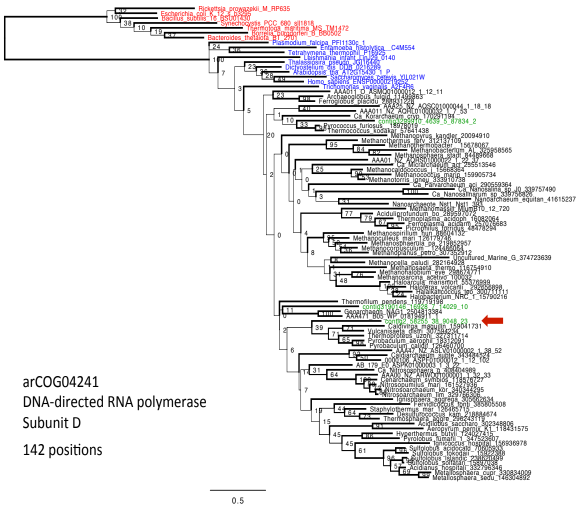
arCOG04241
DNA-directed RNA polymerase
Subunit D
142 positions
0.5

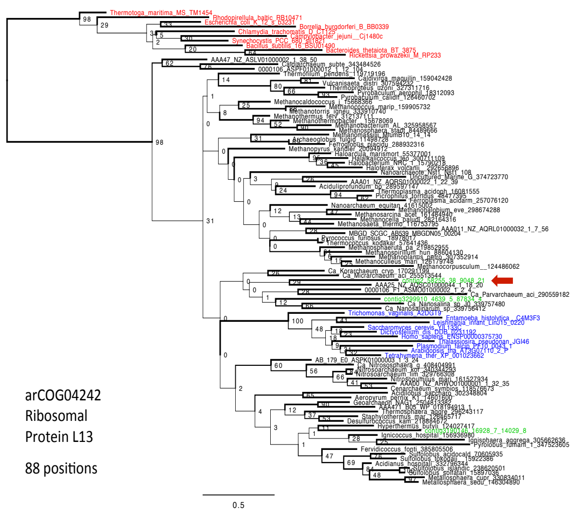
arCOG04242
Ribosomal
Protein L13
88 positions
0.5

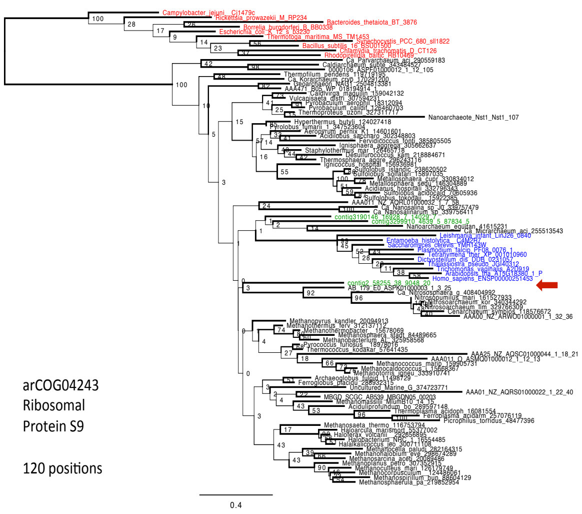
arCOG04243
Ribosomal
Protein S9
120 positions
0.4

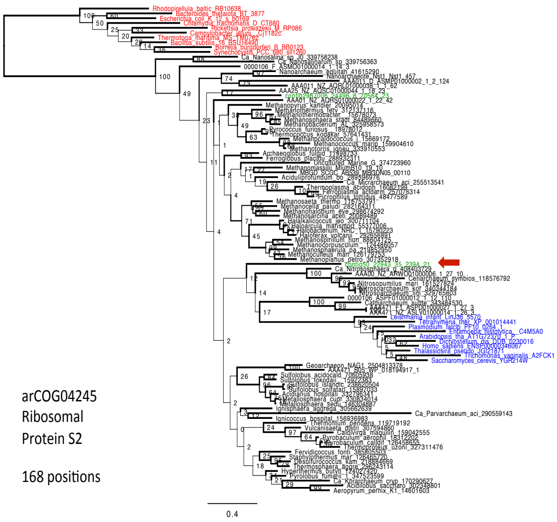
arCOG04245
Ribosomal
Protein S2

168 positions

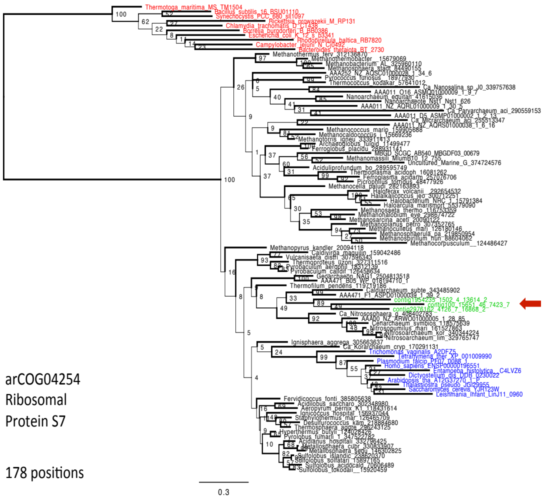
arCOG04254
Ribosomal
Protein S7

178 positions

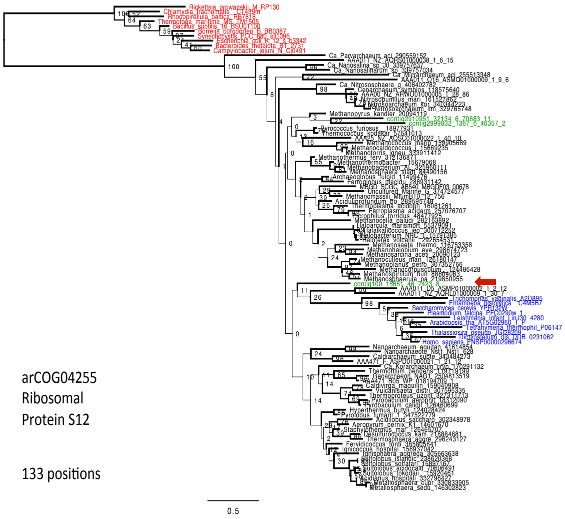
arCOG04255
Ribosomal
Protein S12

133 positions

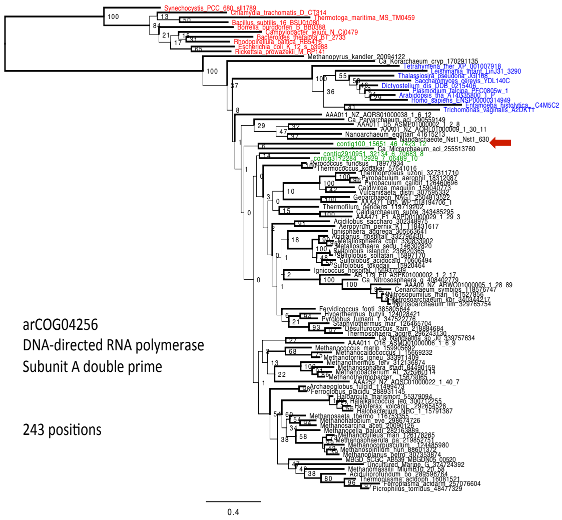
arCOG04256
DNA-directed RNA polymerase
Subunit A double prime

243 positions

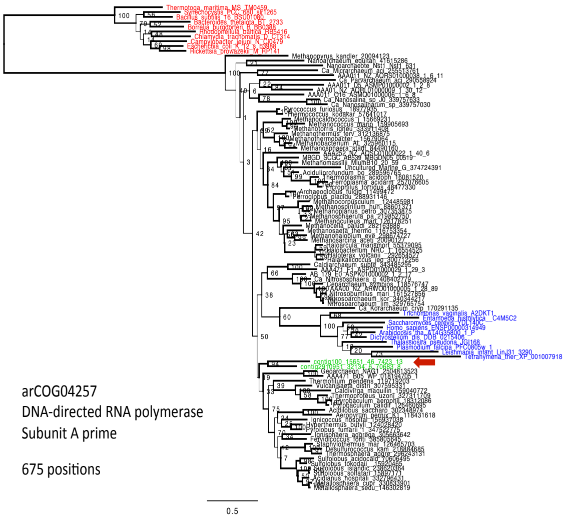
arCOG04257
DNA-directed RNA polymerase
Subunit A prime

675 positions

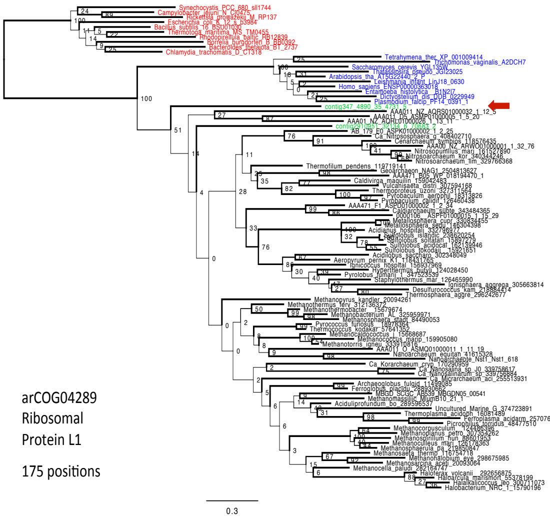
arCOG04289
Ribosomal
Protein L1

175 positions
