# Supplementary material for: Lokiarchaea are close relatives of Euryarchaeota, not bridging the gap between prokaryotes and eukaryotes
Source: PLoS Genet. 2017 Jun 12;13(6):e1006810. doi: 10.1371/journal.pgen.1006810 (PMC5484517; doi:10.1371/journal.pgen.1006810)

S2 Fig - ML single protein trees of the 36 genes with the curated datasets.
For all trees, the scale-bar indicates the average number of substitutions per site, and values at nodes represent support calculated by nonparametric bootstrap (out of 100). Bacterial and eukaryotic sequences are indicated in red and blue respectively, while lokiarchaeal sequences are indicated in green. In each tree, a red arrow indicates the lokiarchaeal sequence corresponding to Lokiarchaeon 1. The trees corresponding to the arCOG00412, arCOG01183, and arCOG01559 display more colours as they are representative of the different patterns observed among the trees: the lokiarchaeal sequences within Archaea, the lokiarchaeal sequences at different positions with one being sister group to Eukaryotes, and all the lokiarchaeal sequences sister group to Eukarya, respectively. In these trees, Crenarchaeota, Euryarchaeota, and Thaumarchaeota are indicated in orange, green, and pink, respectively.

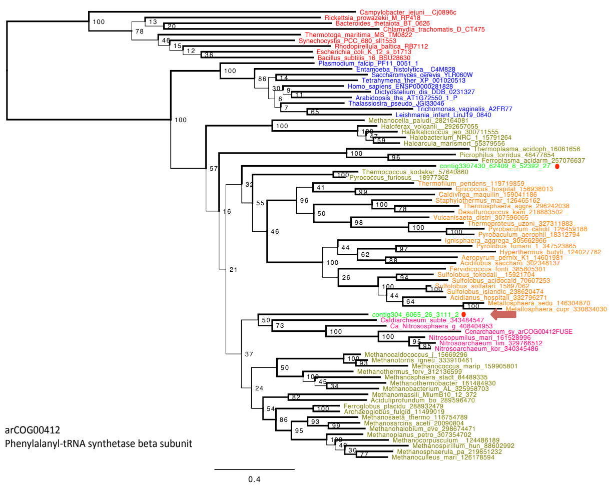

arCOG00412
Phenylalanyl-tRNA synthetase beta subunit

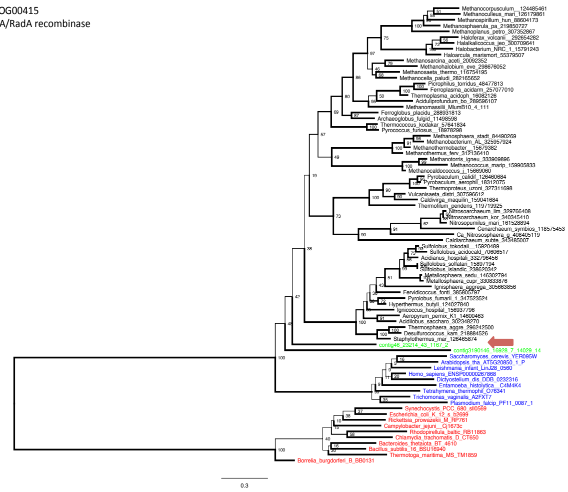

arCOG00415
RecA/RadA recombinase

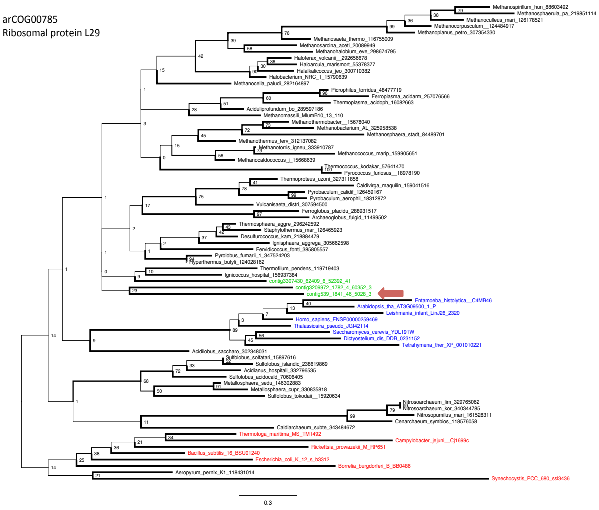

arCOG00785
Ribosomal protein L29

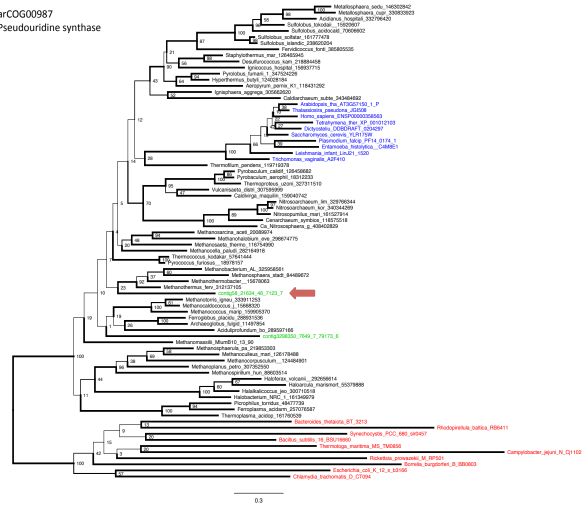

arCOG00987
Pseuduridine synthase

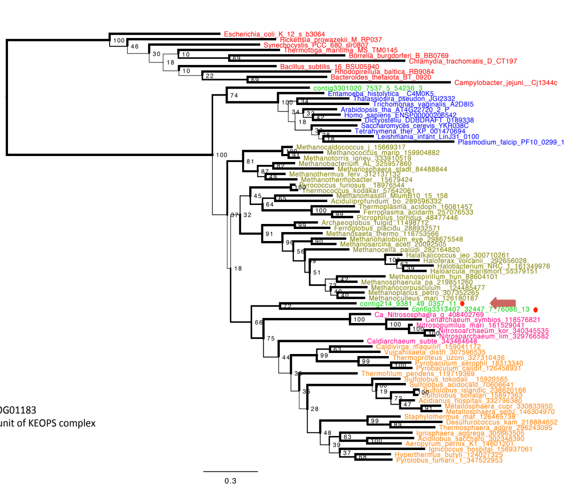

arCOG01183
Subunit of KEOPS complex

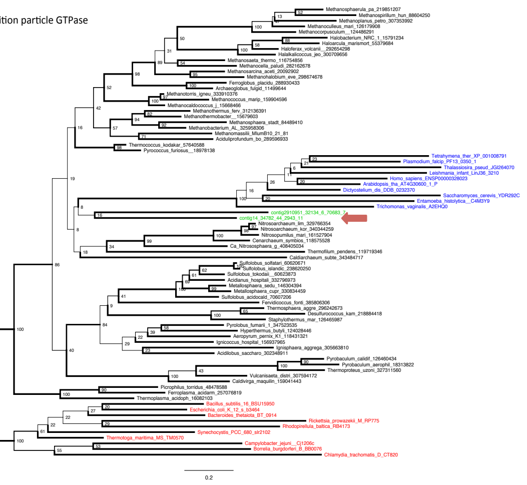

arCOG01227
Signal recognition particle GTPase

arCOG01228
Signal recognition particle GTPase

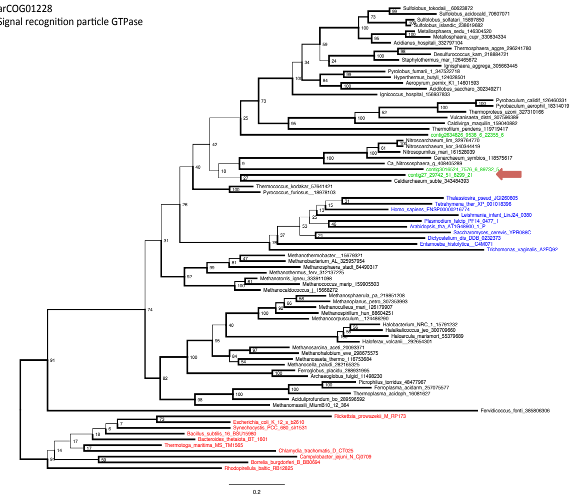

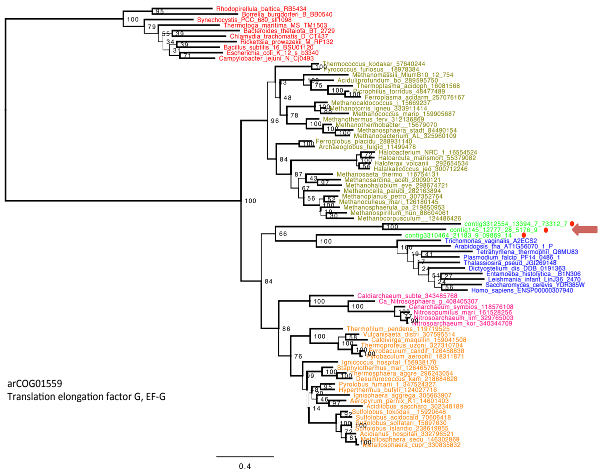

arCOG01559
Translation elongation factor G, EF-G

arCOG01560
Translation initiation factor 2, IF-2

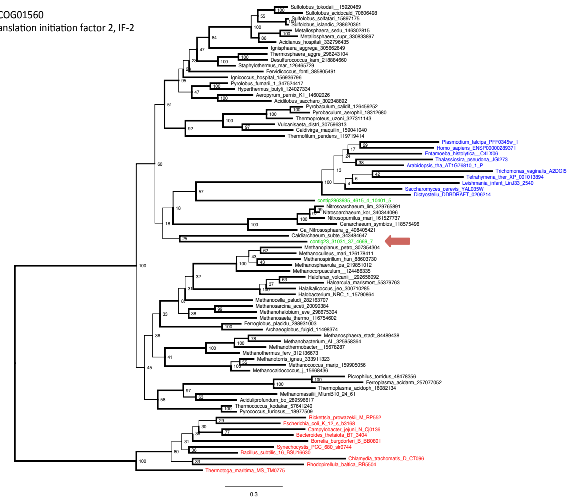

arCOG01722
Ribosomal protein S13

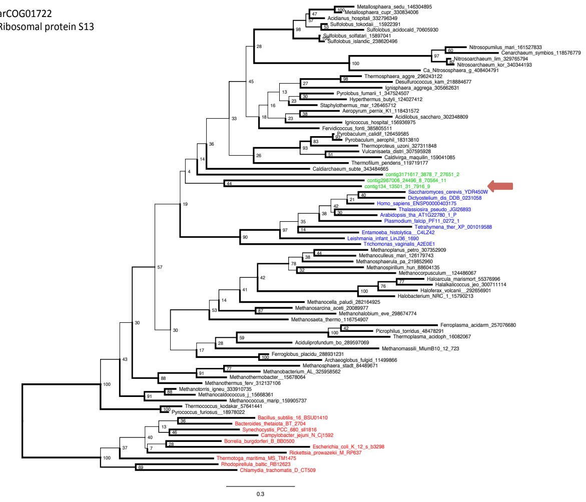

arCOG01758
Ribosomal protein S10

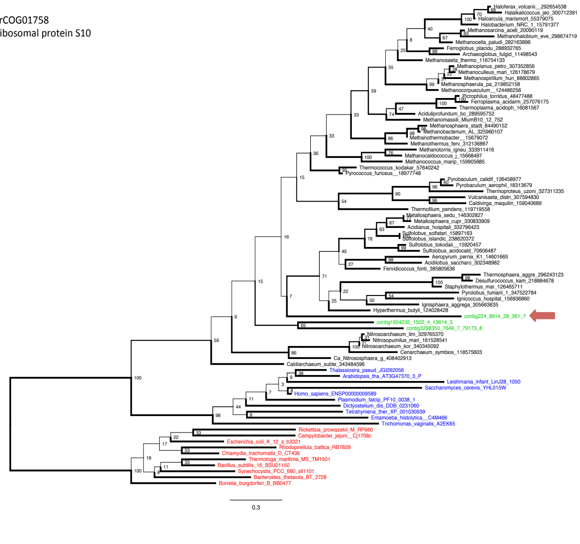

arCOG01762
DNA-directed RNA polymerase subunit B

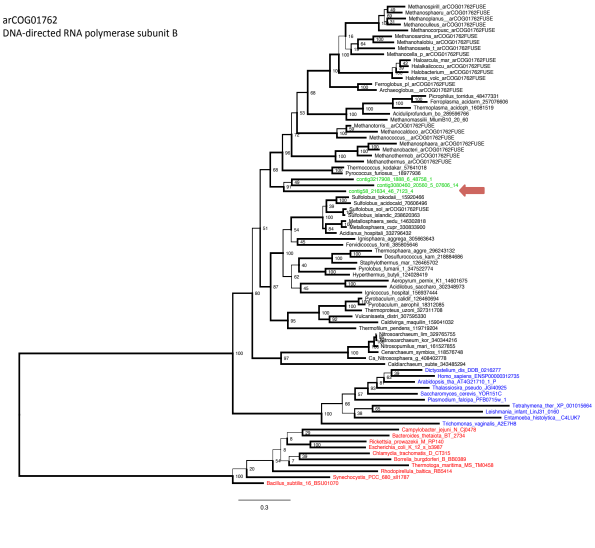

arCOG04064
Predicted membrane-associated Zn-dependent protease

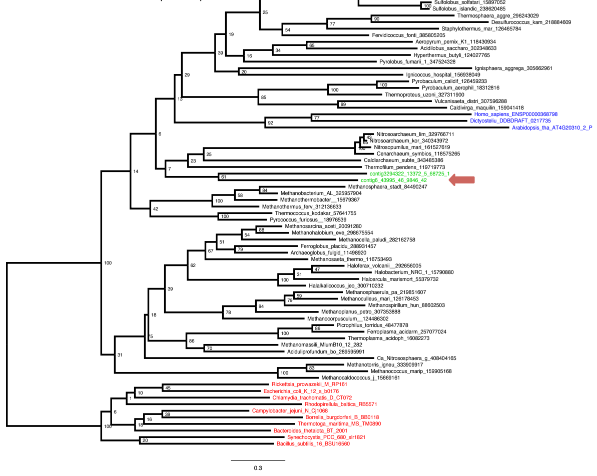

arCOG04090
Ribosomal protein L6P

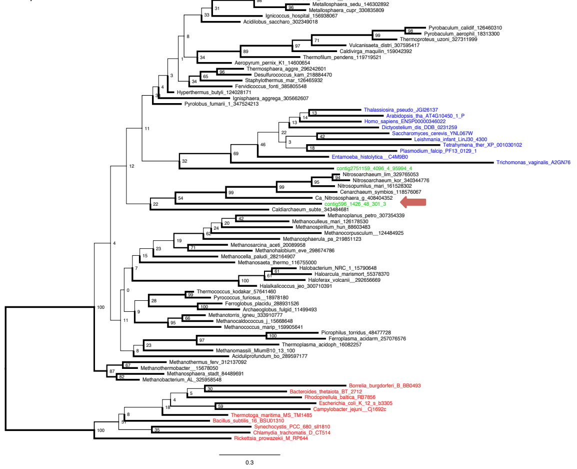

arCOG04091
Ribosomal protein S8

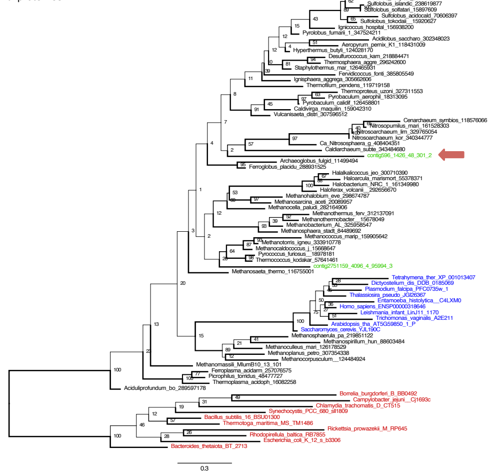

arCOG04092
Ribosomal protein L5

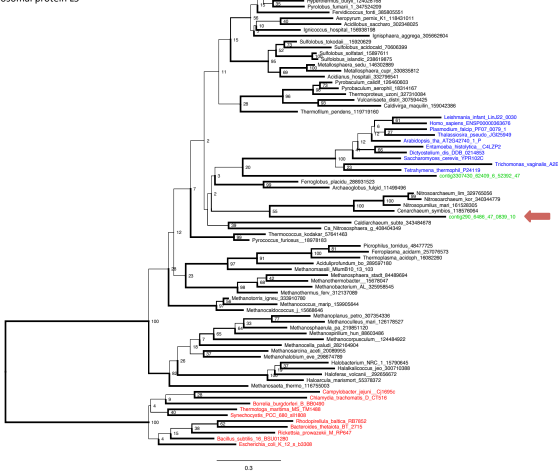

arCOG04094
Ribosomal protein L24

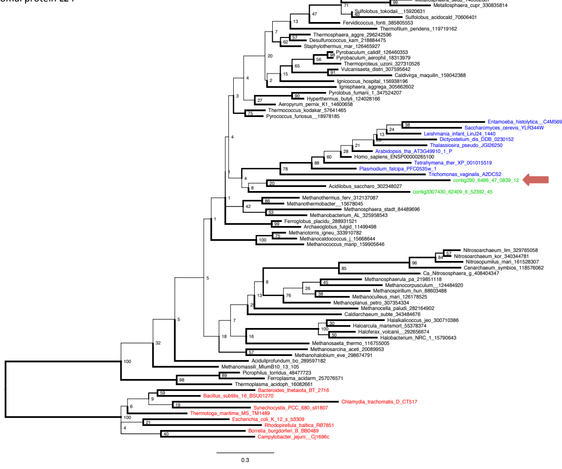

arCOG04095
Ribosomal protein L14

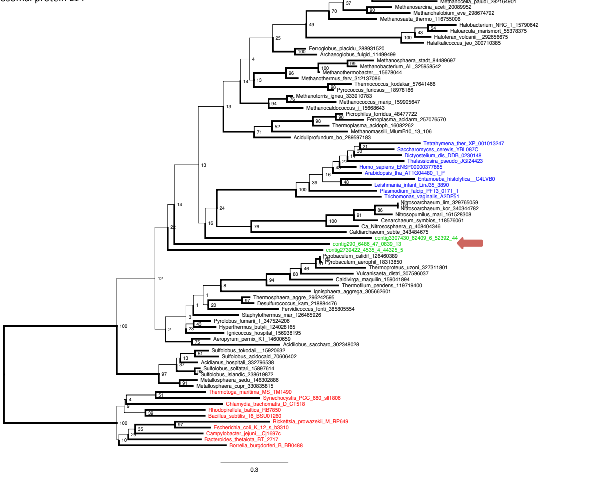

arCOG04096
Ribosomal protein S17

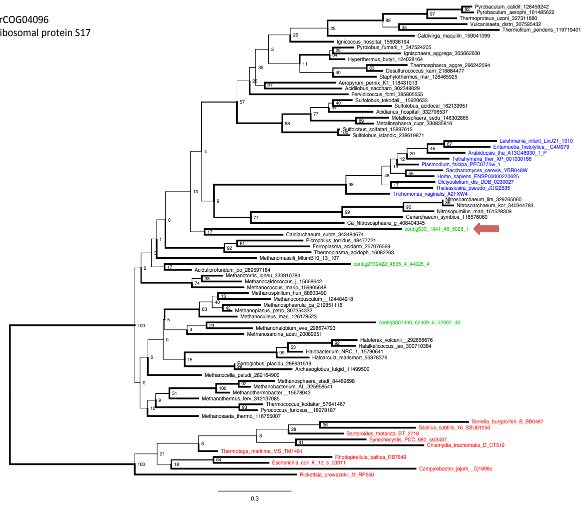

arCOG04097
Ribosomal protein S3

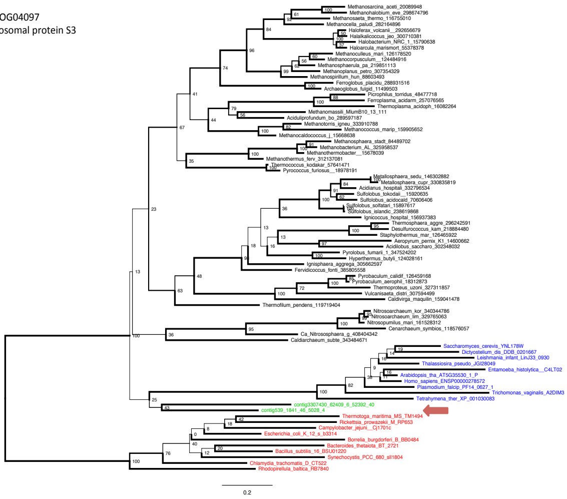

arCOG04098
Ribosomal protein L22

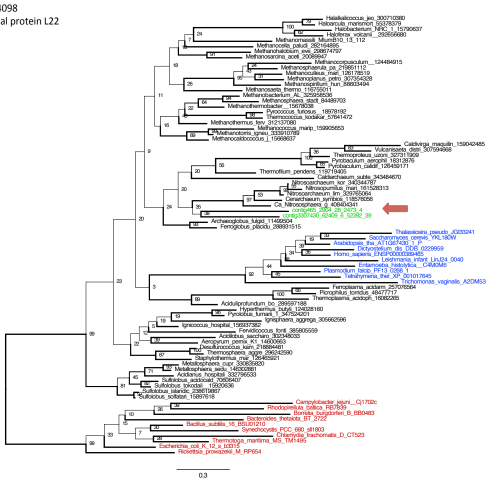

arCOG04099
Ribosomal protein S19

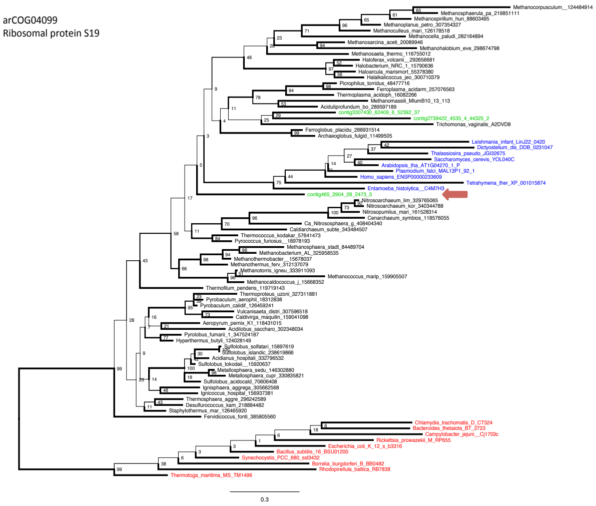

arCOG04113
Ribosomal protein L10AE/16

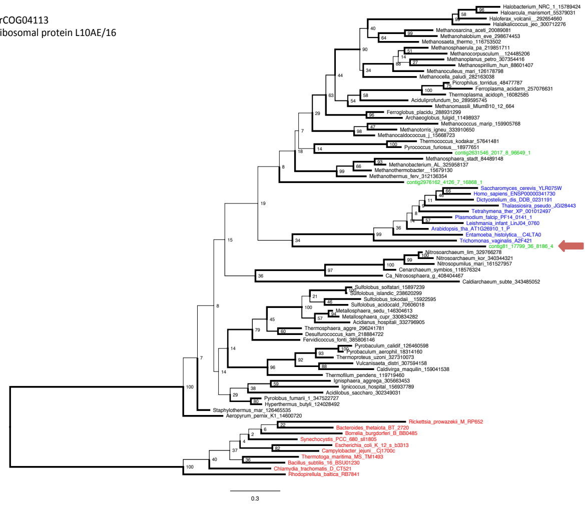

arCOG04121
Ribonuclease HII

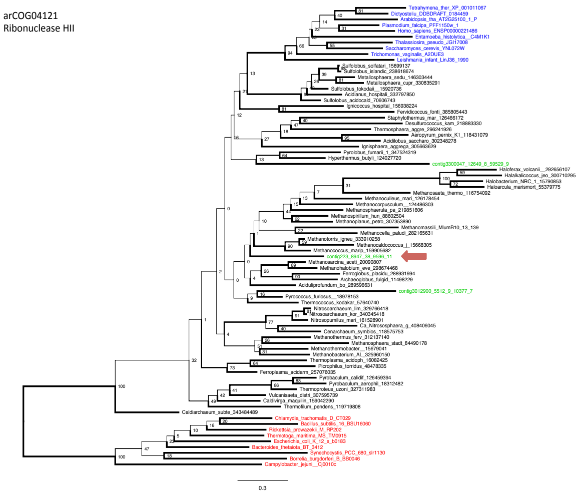

arCOG04169
Preprotein translocase subunit SecY

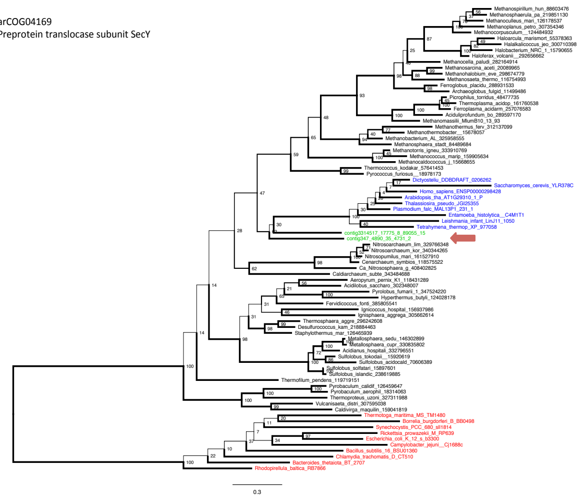

arCOG04239
Ribosomal protein S4

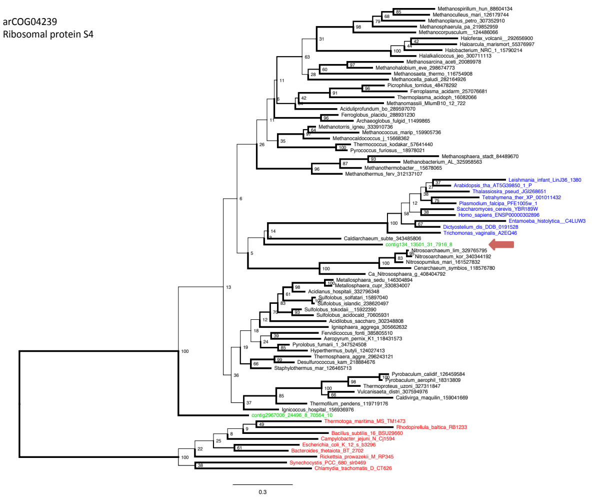

arCOG04240
Ribosomal protein S11

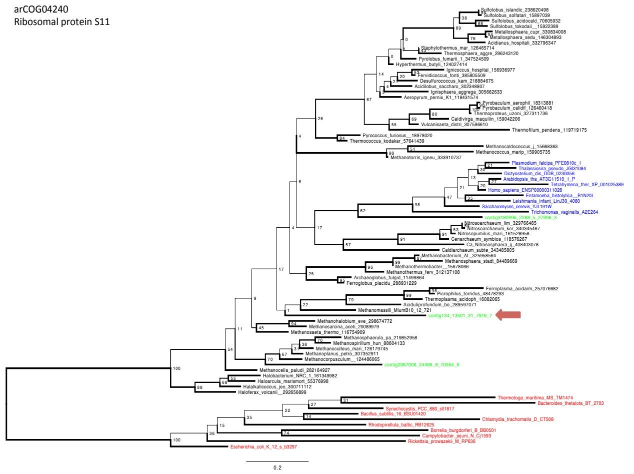

arCOG04241
DNA-directed RNA polymerase subunit D

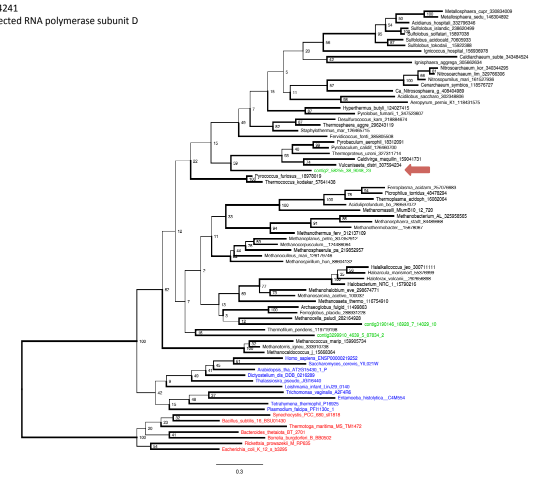

arCOG04242
Ribosomal protein L13

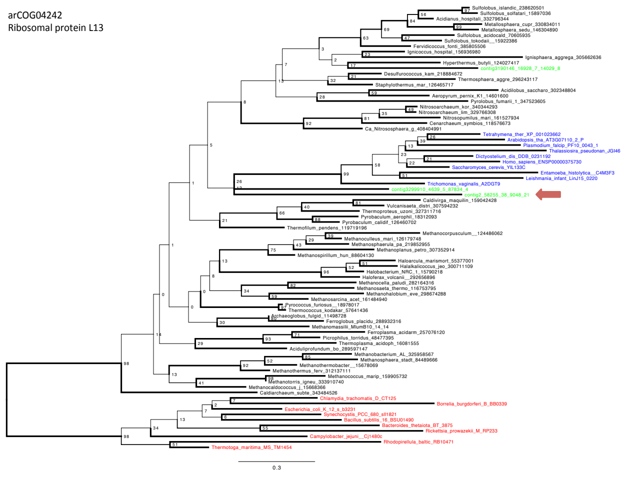

arCOG04243
Ribosomal protein S9

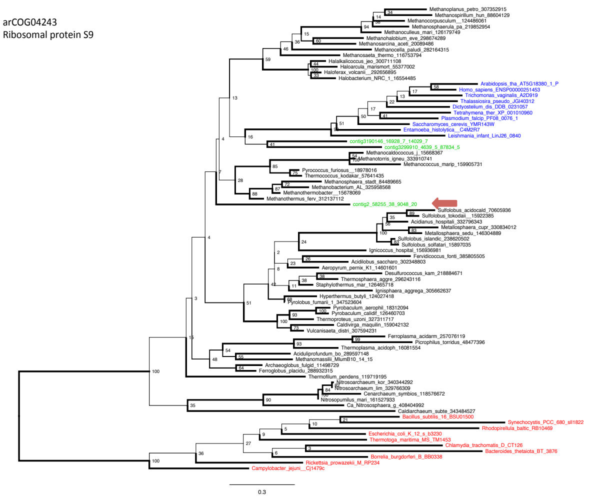

arCOG04245
Ribosomal protein S2

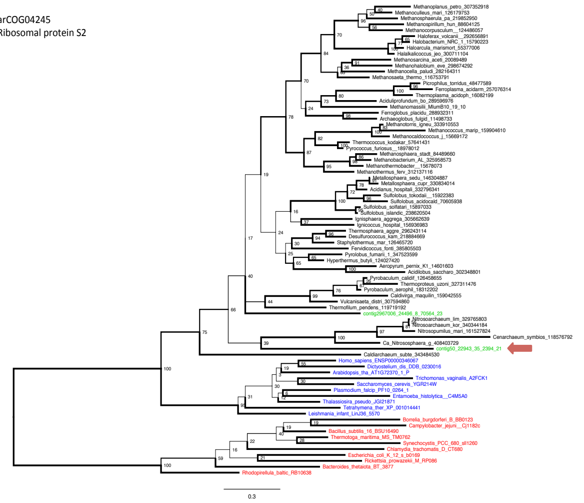

arCOG04254
Ribosomal protein S7

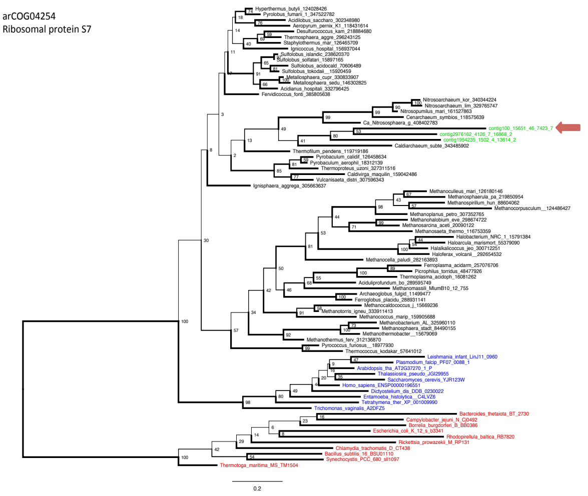

arCOG04255
Ribosomal protein S12

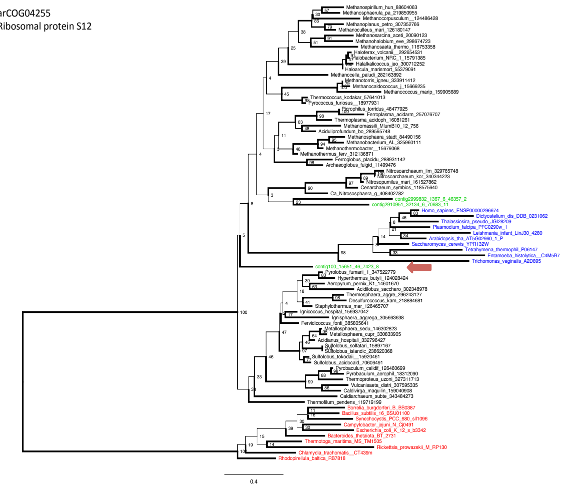

arCOG04256
DNA-directed RNA polymerase subunit A''

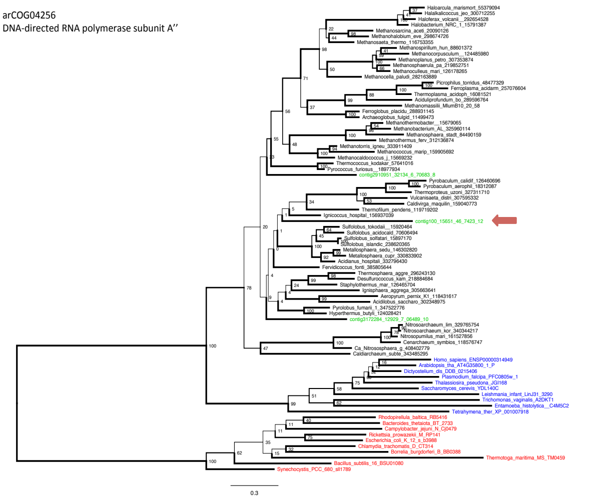

arCOG04257
DNA-directed RNA polymerase subunit A'

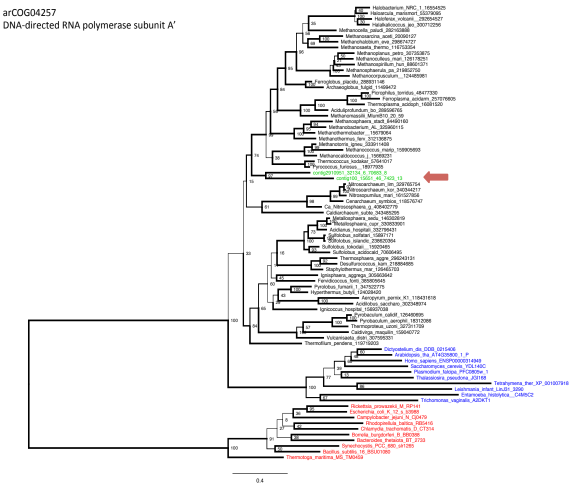

arCOG04289
Ribosomal protein L1

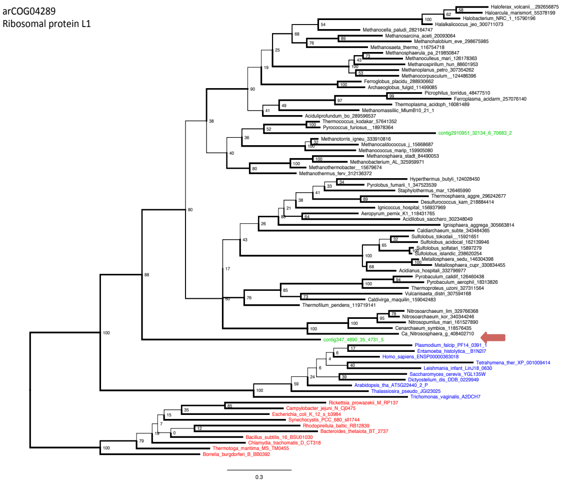

Supplement: S2 Fig — For all trees, the scale-bar indicates the average number of substitutions per site, and values at nodes represent support calculated by nonparametric bootstrap (out of 100). Bacterial and eukaryotic sequences are indicated in red and blue respectively, while lokiarchaeal sequences are indicated in green. In each tree, a red arrow indicates the lokiarchaeal sequence corresponding to Lokiarchaeon 1. The trees corresponding to the arCOG00412, arCOG01183, and arCOG01559 display more colours as they are representative of the different patterns observed among the trees: the lokiarchaeal sequences within Archaea, the lokiarchaeal sequences at different positions with one being sister group to Eukaryotes, and all the lokiarchaeal sequences sister group to Eukarya, respectively. In these trees, Crenarchaeota, Euryarchaeota, and Thaumarchaeota are indicated in orange, green, and pink, respectively. (PDF) [file pgen.1006810.s002.pdf]
