# Supplementary material for: Lokiarchaea are close relatives of Euryarchaeota, not bridging the gap between prokaryotes and eukaryotes
Source: PLoS Genet. 2017 Jun 12;13(6):e1006810. doi: 10.1371/journal.pgen.1006810 (PMC5484517; doi:10.1371/journal.pgen.1006810)
Supplement: S9 Fig — In this tree, bacterial and eukaryotic sequences are indicated in red and blue, respectively. For Archaea, Thaumarchaeota and Aigarchaeota are indicated in pink, Crenarchaeota in orange and Euryarchaeota in olive-green. The Lokiarchaeota are indicated in light-green. Values at nodes indicate the Bayesian posterior probabilities. The scale-bar represents the average number of substitutions per site. (PDF) [file pgen.1006810.s009.pdf]

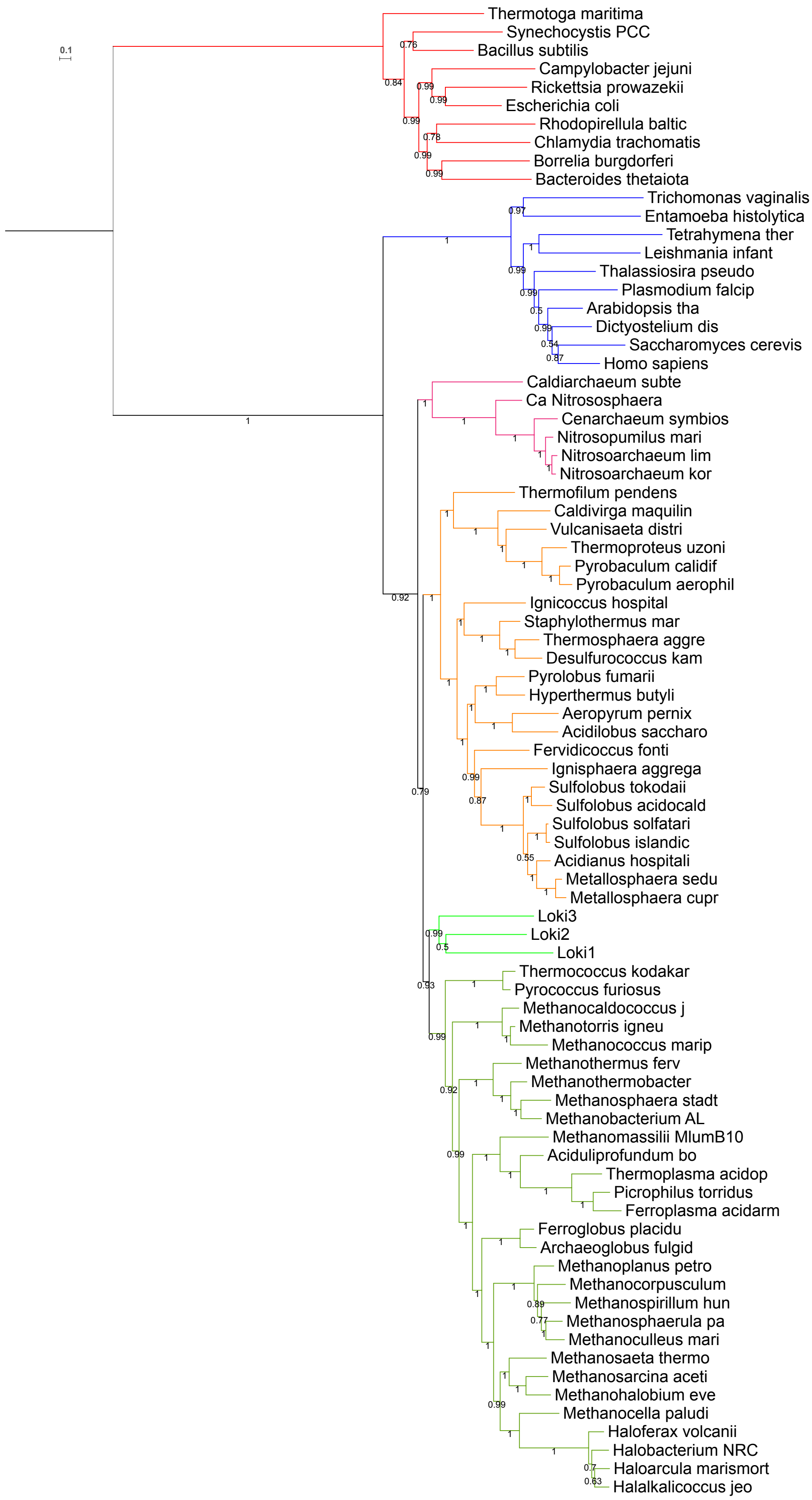

S9 Fig – Bayesian inference phylogeny of the concatenation of the 6 AU-relevant Woese's proteins from the curated datasets (1,857 positions).

In this tree, bacterial and eukaryotic sequences are indicated in red and blue, respectively. For Archaea, Thaumarchaeota and Aigarchaeota are indicated in pink, Crenarchaeota in orange and Euryarchaeota in olive-green. The Lokiarchaeota are indicated in light-green. Values at nodes indicate the Bayesian posterior probabilities. The scale-bar represents the average number of substitutions per site.
