# Supplementary material for: Lokiarchaea are close relatives of Euryarchaeota, not bridging the gap between prokaryotes and eukaryotes
Source: PLoS Genet. 2017 Jun 12;13(6):e1006810. doi: 10.1371/journal.pgen.1006810 (PMC5484517; doi:10.1371/journal.pgen.1006810)

S12 Fig – The Loki 1 genome quality.

a. Table summarizing the results obtained with CheckM and Anvi'o on Loki 1 genome quality. **b.** Graphical view of the Anvi'o interactive display of the Lokiarchaeum genome (Loki 1). The clustering dendrogram in the center displays the hierarchical contigs clustering based on their tetra-nucleotide sequence composition and their differential reads coverage across the different sequencing runs. Each of the 513 tips represents a contig or a split contig as Anvi'o splits contigs too long. These are still located together and noticed by a grey bar on the upper layer ("parent" layer). The length and GC layers show the relative length and GC-content of a contig. The additional layers represent the relative abundance (coverage) of each contig in the different sequencing runs (SRR1555743, SRR1555748, SRR1555750). The green stars indicate the position of the two contigs encoding the RNA polymerase subunits A and B genes used in the different concatenations. The orange star indicate the position of the contig encoding EF2. The table on the bottom gives additional information regarding the sets suggested by this analysis, notably their length and composition, and the results of different combinations of sets.

a.

Software	Number of markers	Completeness	Contamination	Heterogeneity index
**CheckM**	145	90.29%	45.15 %	78.21
**Anvi'o**	162	92,6%	56,8%	-

b.

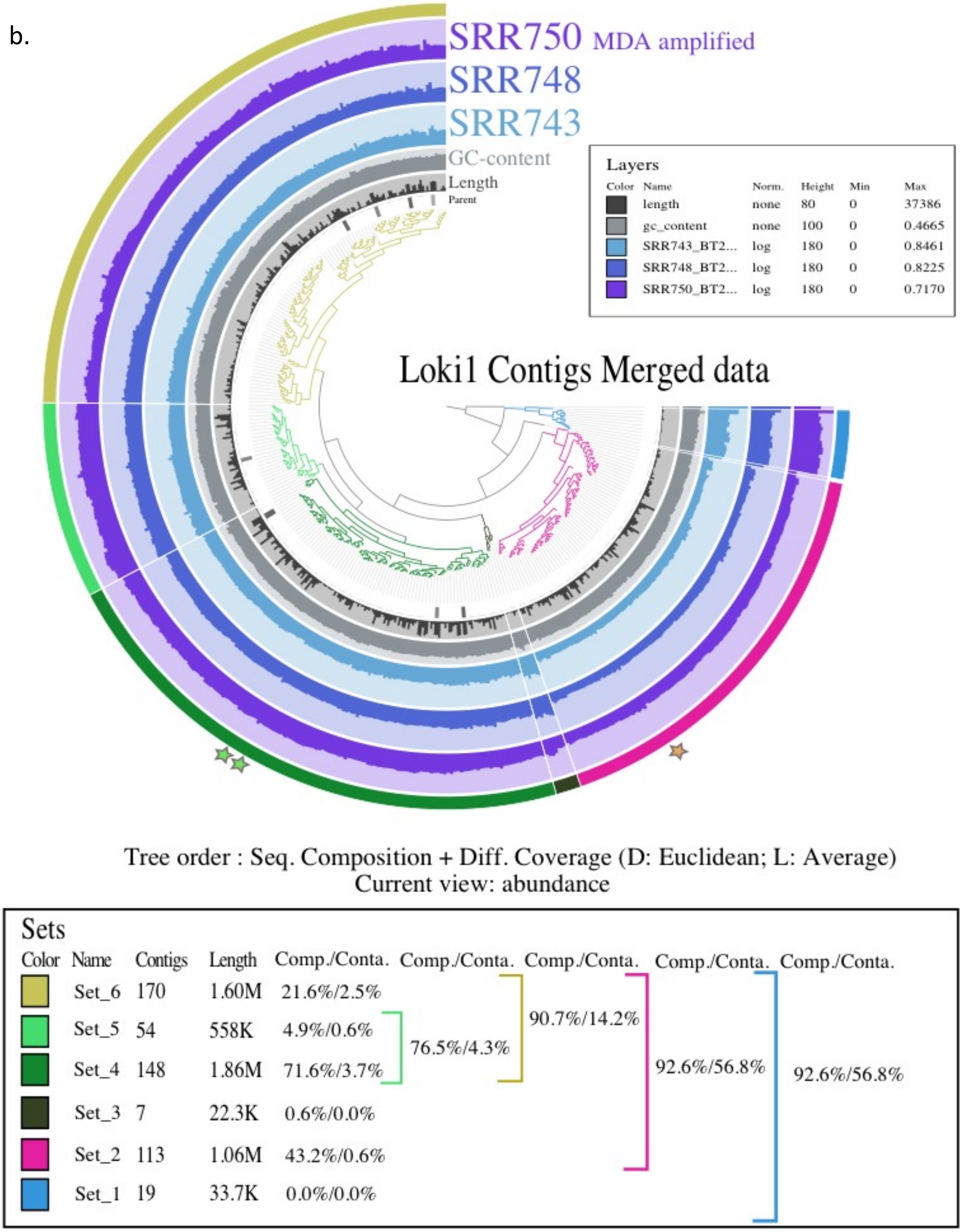

Supplement: S12 Fig — a. Table summarizing the results obtained with CheckM and Anvi’o on Loki 1 genome quality. b. Graphical view of the Anvi’o interactive display of the Lokiarchaeum genome (Loki 1). The clustering dendrogram in the center displays the hierarchical contigs clustering based on their tetra-nucleotide sequence composition and their differential reads coverage across the different sequencing runs. Each of the 513 tips represents a contig or a split contig as Anvi’o splits contigs too long. These are still located together and noticed by a grey bar on the upper layer (“parent” layer). The length and GC layers show the relative length and GC-content of a contig. The additional layers represent the relative abundance (coverage) of each contig in the different sequencing runs (SRR1555743, SRR1555748, SRR1555750). The green stars indicate the position of the two contigs encoding the RNA polymerase subunits A and B genes used in the different concatenations. The orange star indicate the position of the contig encoding EF2. The table on the bottom gives additional information regarding the sets suggested by this analysis, notably their length and composition, and the results of different combinations of sets. (PDF) [file pgen.1006810.s012.pdf]
