# Supplementary material for: Lokiarchaea are close relatives of Euryarchaeota, not bridging the gap between prokaryotes and eukaryotes
Source: PLoS Genet. 2017 Jun 12;13(6):e1006810. doi: 10.1371/journal.pgen.1006810 (PMC5484517; doi:10.1371/journal.pgen.1006810)
Supplement: S13 Fig — Alignment of the region corresponding to the insertion A1, A2 and A3 in lokiarchaeal EF2 sequences, with archaeal EF2 sequences and eukaryotic Ria sequences (EF2 paralog), and with Ria sequences from a subgroup of fungi (bottom alignment). Organisms’ names corresponding to Lokiarchaea/Thorarchaea, Archaea, and Eukarya are respectively indicated in brown, green, and blue. (PDF) [file pgen.1006810.s013.pdf]

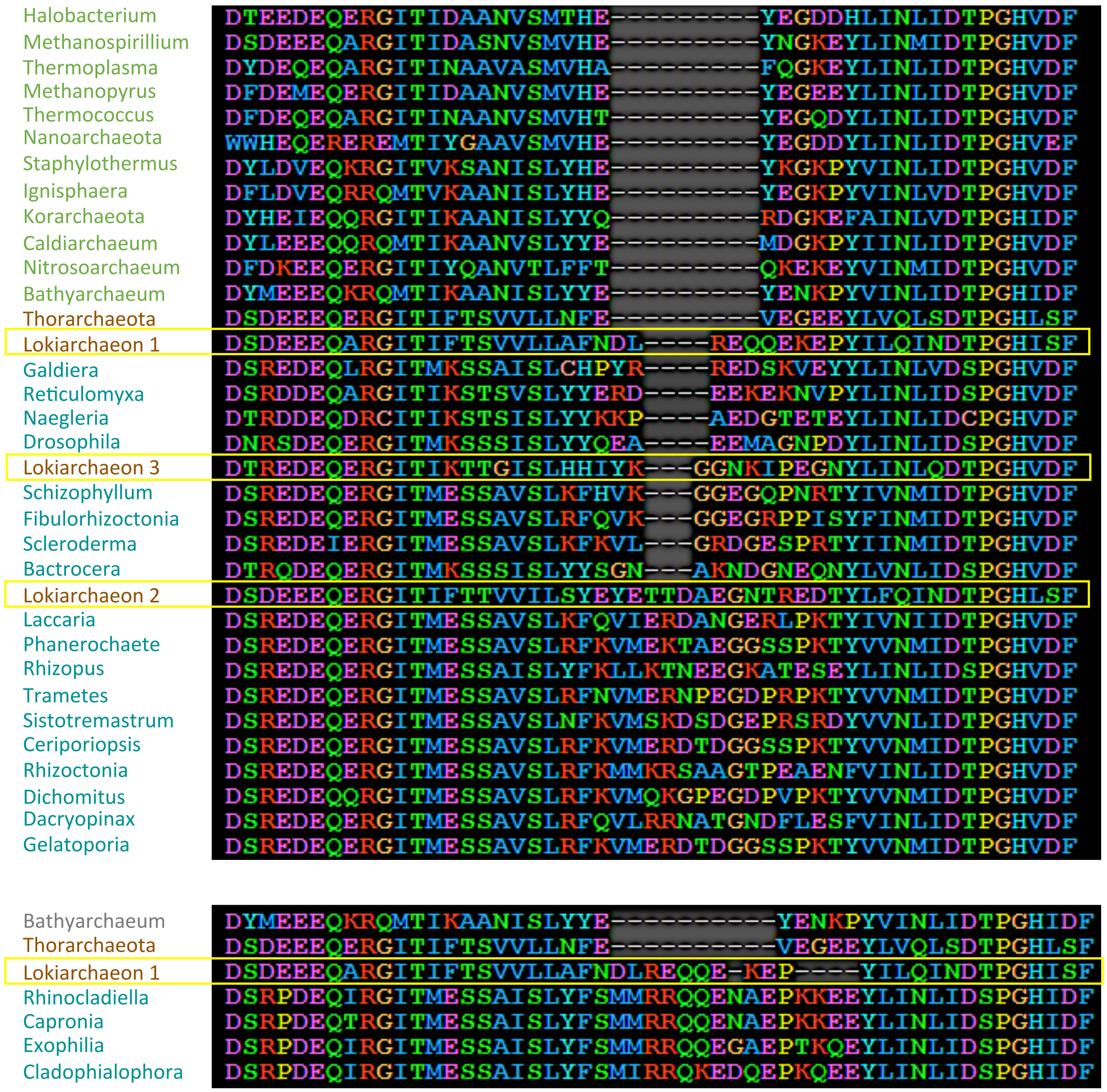

S13 Fig – Alignment of the N-terminal lokiarchaeal EF2 insertion (A1, A2, A3).
Alignment of the region corresponding to the insertion A1, A2 and A3 in lokiarchaeal EF2 sequences, with archaeal EF2 sequences and eukaryotic Ria sequences (EF2 paralog), and with Ria sequences from a subgroup of fungi (bottom alignment). Organisms' names corresponding to Lokiarchaea/Thorarchaea, Archaea, and Eukarya are respectively indicated in brown, green, and blue.
