# Supplementary material for: Lokiarchaea are close relatives of Euryarchaeota, not bridging the gap between prokaryotes and eukaryotes
Source: PLoS Genet. 2017 Jun 12;13(6):e1006810. doi: 10.1371/journal.pgen.1006810 (PMC5484517; doi:10.1371/journal.pgen.1006810)
Supplement: S14 Fig — Alignment of the region corresponding to the B3 insertion (located in positions 268 to 323 of the Loki 3 EF2 protein) with archaeal EF2 sequences and eukaryotic Ria sequences (EF2 paralog). Organisms’ names corresponding to Lokiarchaea/Thorarchaea, Archaea, and Eukarya are respectively indicated in brown, green, and blue. (PDF) [file pgen.1006810.s014.pdf]

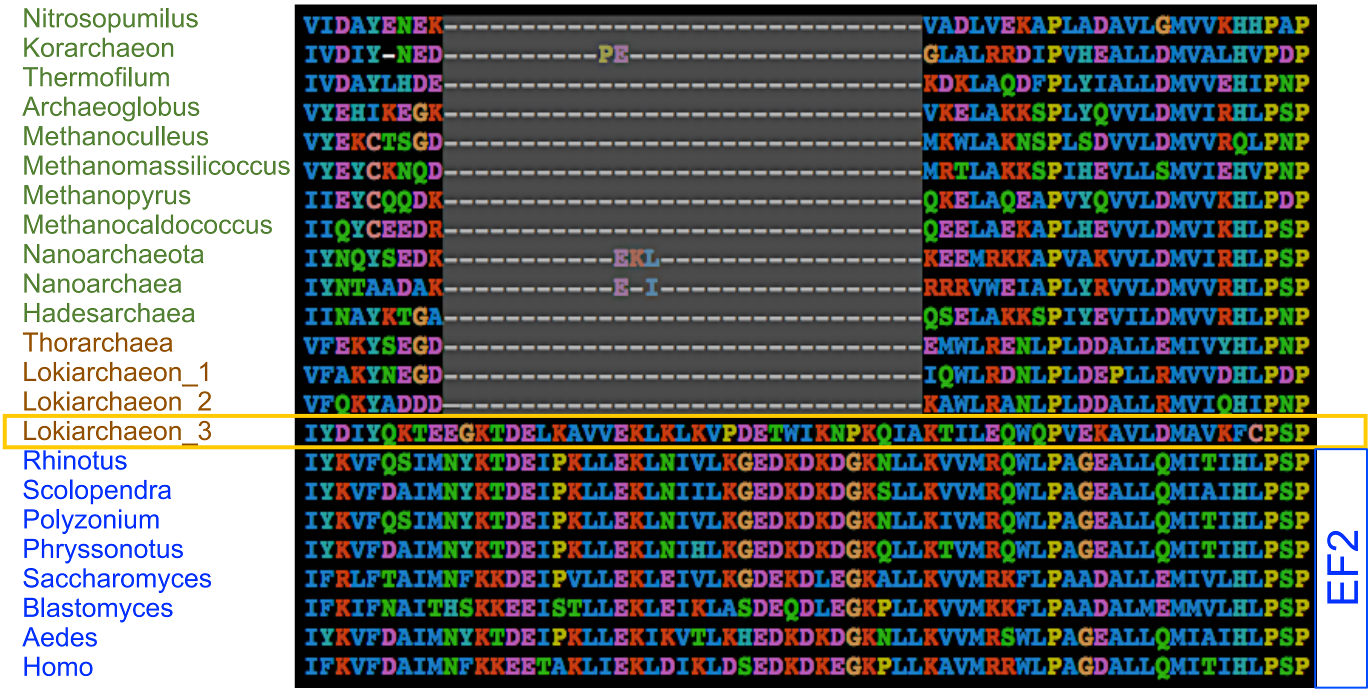

S14 Fig – Alignment of insertion B3 of the Loki 3 EF2 protein.

Alignment of the region corresponding to the B3 insertion (located in positions 268 to 323 of the Loki 3 EF2 protein) with archaeal EF2 sequences and eukaryotic Ria sequences (EF2 paralog). Organisms' names corresponding to Lokiarchaea/Thorarchaea, Archaea, and Eukarya are respectively indicated in brown, green, and blue.
