# Supplementary material for: Lokiarchaea are close relatives of Euryarchaeota, not bridging the gap between prokaryotes and eukaryotes
Source: PLoS Genet. 2017 Jun 12;13(6):e1006810. doi: 10.1371/journal.pgen.1006810 (PMC5484517; doi:10.1371/journal.pgen.1006810)
Supplement: S15 Fig — Alignment of the region corresponding to the C3 insertion (located in positions 373 to 406 of the Loki 3 EF2 protein) with archaeal and eukaryotic EF2 sequences and eukaryotic Ria sequences (EF2 paralog). Organisms’ names corresponding to Bacteria, Lokiarchaea/Thorarchaea, Archaea, and Eukarya are respectively indicated in red, brown, green, and blue. (PDF) [file pgen.1006810.s015.pdf]

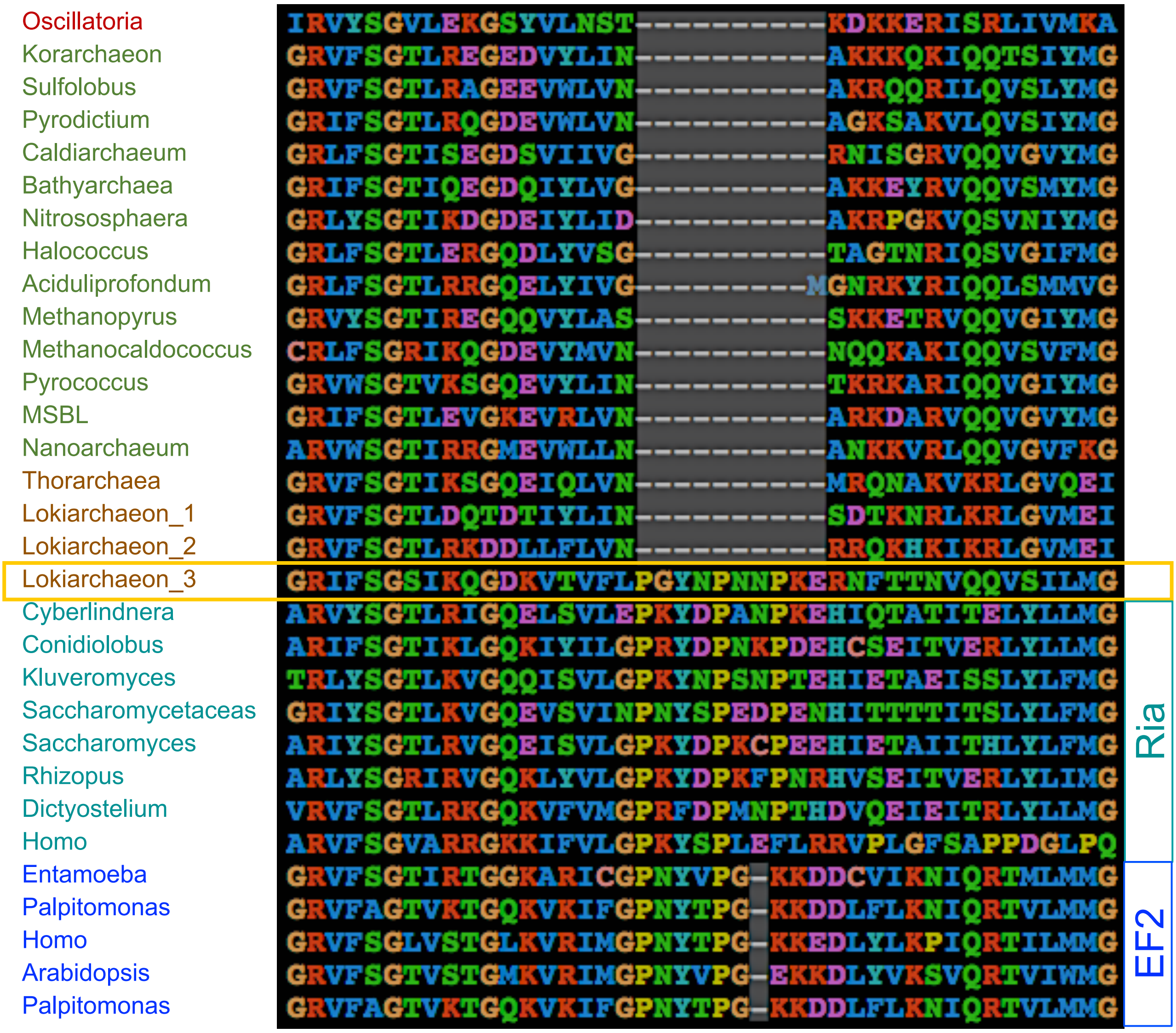

S15 Fig – Alignment of insertion C3 of the Loki 3 EF2 protein.
Alignment of the region corresponding to the C3 insertion (located in positions 373 to 406 of the Loki 3 EF2 protein) with archaeal and eukaryotic EF2 sequences and eukaryotic Ria sequences (EF2 paralog). Organisms' names corresponding to Bacteria, Lokiarchaea/Thorarchaea, Archaea, and Eukarya are respectively indicated in red, brown, green, and blue.
