# Supplementary material for: Lokiarchaea are close relatives of Euryarchaeota, not bridging the gap between prokaryotes and eukaryotes
Source: PLoS Genet. 2017 Jun 12;13(6):e1006810. doi: 10.1371/journal.pgen.1006810 (PMC5484517; doi:10.1371/journal.pgen.1006810)
Supplement: S17 Fig — a. ML phylogeny obtained with the N-terminal section of EF2 (232 sites). b. ML phylogeny obtained with the C-terminal section of the protein (394 sites). c. ML phylogeny obtained with the entire EF2 protein (626 sites). In these trees, bacterial and eukaryotic sequences are indicated in red and blue, respectively. For Archaea, Thaumarchaeota and Aigarchaeota are indicated in pink, Crenarchaeota in orange and Euryarchaeota in olive-green. The Lokiarchaea are indicated in light-green. The scale-bar represents the average number of substitutions per site. Values at nodes represent support calculated by ultrafast bootstrap approximation (out of 100; 1,000 replicates). (PDF) [file pgen.1006810.s017.pdf]

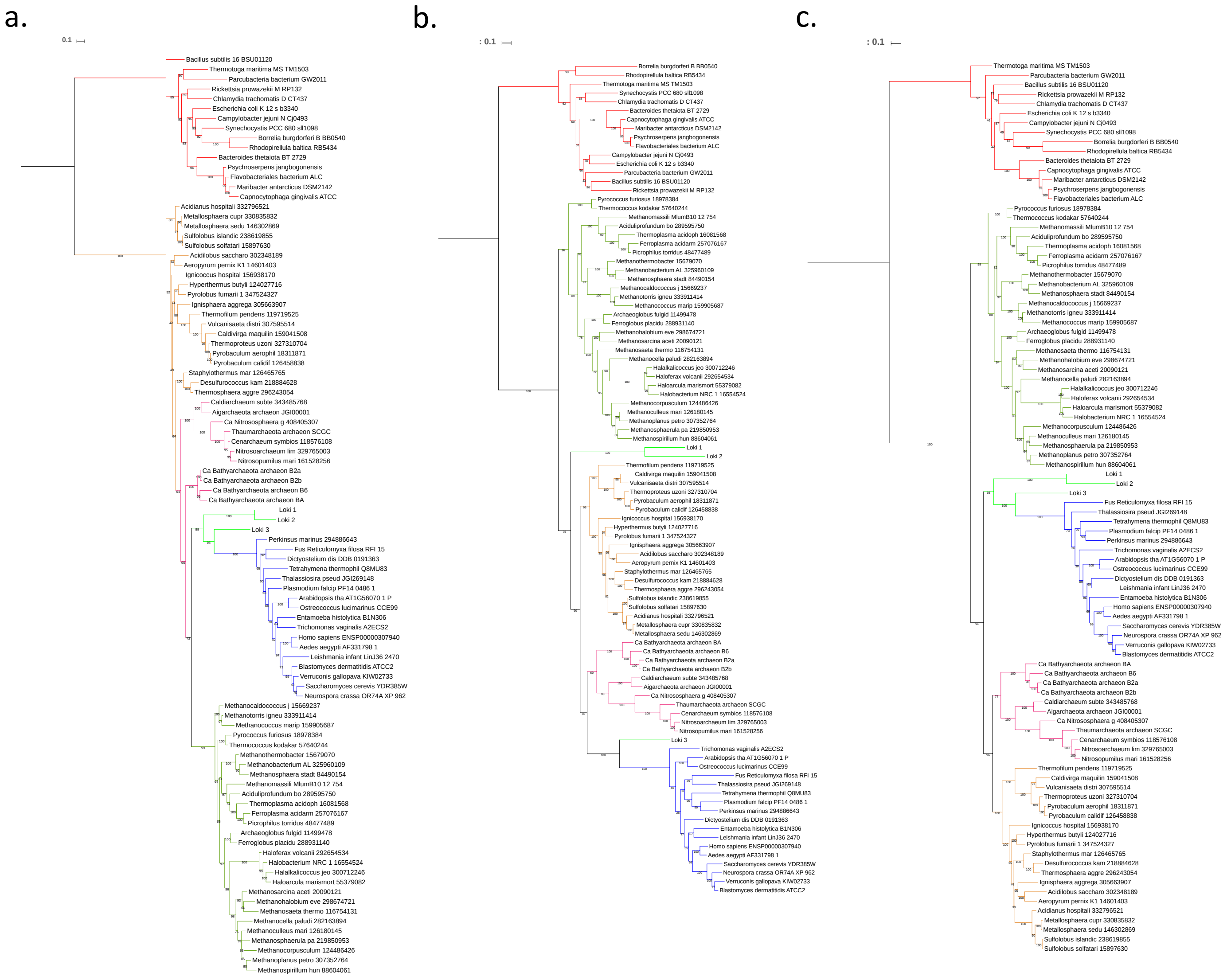

S17 Fig – ML phylogenetic trees of the Elongation Factor 2 (EF2) after inclusion of bathyarchaeal sequences (ultrafast bootstrap approximation).

a. ML phylogeny obtained with the N-terminal section of EF2 (232 sites). **b.** ML phylogeny obtained with the C-terminal section of the protein (394 sites). **c.** ML phylogeny obtained with the entire EF2 protein (626 sites). In these trees, bacterial and eukaryotic sequences are indicated in red and blue, respectively. For Archaea, Thaumarchaeota and Aigarchaeota are indicated in pink, Crenarchaeota in orange and Euryarchaeota in olive-green. The Lokiarchaea are indicated in light-green. The scale-bar represents the average number of substitutions per site. Values at nodes represent support calculated by ultrafast bootstrap approximation (out of 100; 1,000 replicates).
