# Supplementary material for: Lokiarchaea are close relatives of Euryarchaeota, not bridging the gap between prokaryotes and eukaryotes
Source: PLoS Genet. 2017 Jun 12;13(6):e1006810. doi: 10.1371/journal.pgen.1006810 (PMC5484517; doi:10.1371/journal.pgen.1006810)
Supplement: S26 Fig — In this tree, bacterial and eukaryotic sequences are indicated in red and blue, respectively. For Archaea, Thaumarchaeota and Aigarchaeota are indicated in pink, Crenarchaeota in orange and Euryarchaeota in olive-green. The Lokiarchaeota are indicated in light-green. The scale-bar represents the average number of substitutions per site. Values at nodes represent support calculated by nonparametric bootstrap (out of 100). (PDF) [file pgen.1006810.s026.pdf]

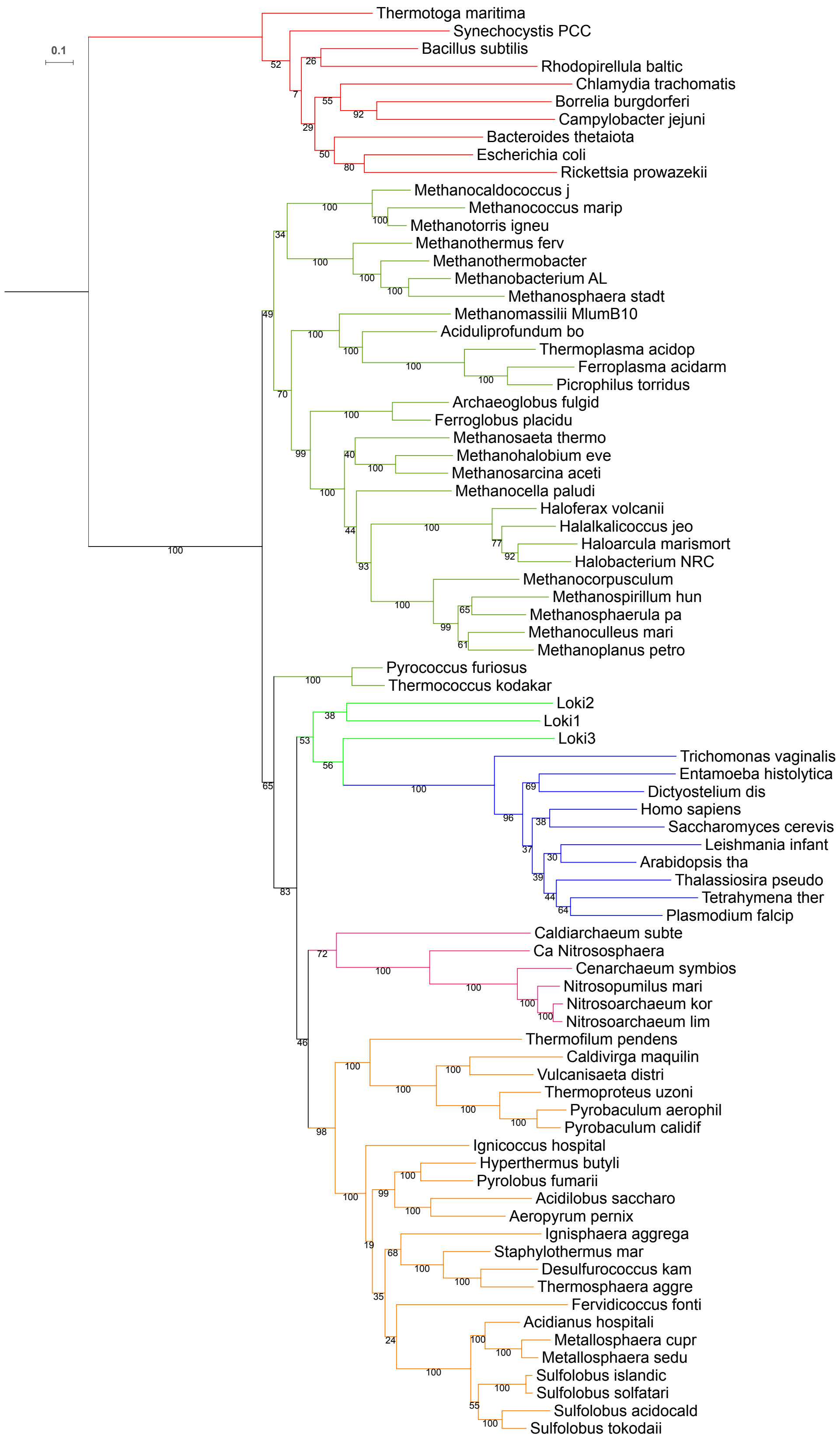

S26 Fig – ML phylogenetic tree of the concatenation of 10 AU-relevant eocyte proteins (all AU-relevant eocyte proteins minus EF2; 2,107 positions).

In this tree, bacterial and eukaryotic sequences are indicated in red and blue, respectively. For Archaea, Thaumarchaeota and Aigarchaeota are indicated in pink, Crenarchaeota in orange and Euryarchaeota in olive-green. The Lokiarchaeota are indicated in light-green. The scale-bar represents the average number of substitutions per site. Values at nodes represent support calculated by nonparametric bootstrap (out of 100).
