# Supplementary material for: Lokiarchaea are close relatives of Euryarchaeota, not bridging the gap between prokaryotes and eukaryotes
Source: PLoS Genet. 2017 Jun 12;13(6):e1006810. doi: 10.1371/journal.pgen.1006810 (PMC5484517; doi:10.1371/journal.pgen.1006810)
Supplement: S28 Fig — In this tree, bacterial and eukaryotic sequences are indicated in red and blue, respectively. For Archaea, Thaumarchaeota and Aigarchaeota are indicated in pink, Crenarchaeota in orange and Euryarchaeota in olive-green. The Thorarchaea (Candidatus Thorarchaeota archaea) are indicated in black. The scale-bar represents the average number of substitutions per site. Values at nodes represent support calculated by nonparametric bootstrap (out of 100). (PDF) [file pgen.1006810.s028.pdf]

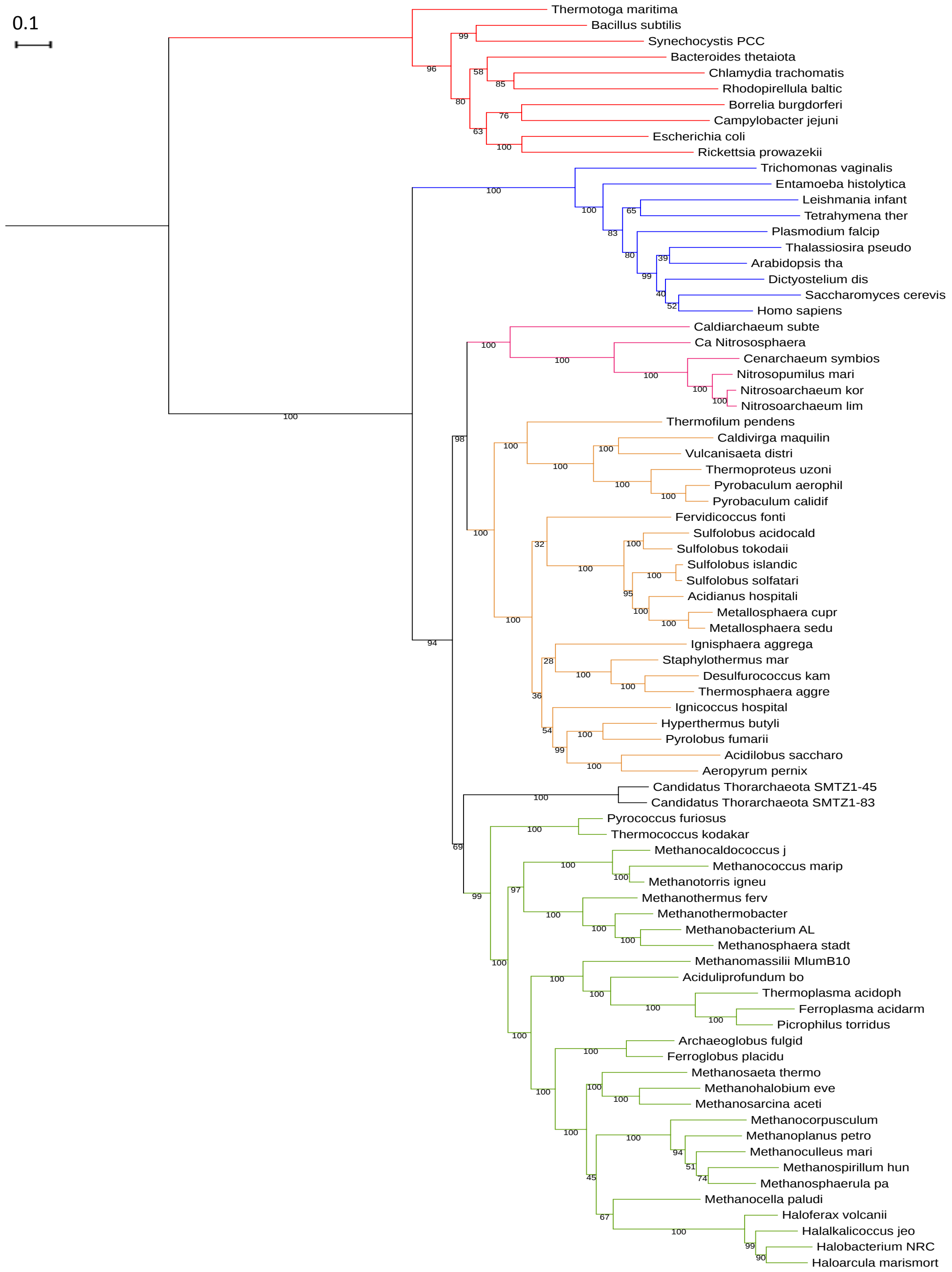


**S28 Fig – ML phylogenetic tree of the concatenation of the 34 arCOGs from the curated datasets present in the two most complete thorarchaeal genomes (8,840 positions).**

In this tree, bacterial and eukaryotic sequences are indicated in red and blue, respectively. For Archaea, Thaumarchaeota and Aigarchaeota are indicated in pink, Crenarchaeota in orange and Euryarchaeota in olive-green. The Thorarchaea (*Candidatus* Thorarchaeota archaea) are indicated in black. The scale-bar represents the average number of substitutions per site. Values at nodes represent support calculated by nonparametric bootstrap (out of 100).
