# Supplementary material for: Lokiarchaea are close relatives of Euryarchaeota, not bridging the gap between prokaryotes and eukaryotes
Source: PLoS Genet. 2017 Jun 12;13(6):e1006810. doi: 10.1371/journal.pgen.1006810 (PMC5484517; doi:10.1371/journal.pgen.1006810)
Supplement: S29 Fig — Organisms’ name corresponding to Bacteria, Lokiarchaea/Thorarchaea, Archaea, and Eukarya are respectively indicated in red, brown, green, and blue. (PDF) [file pgen.1006810.s029.pdf]

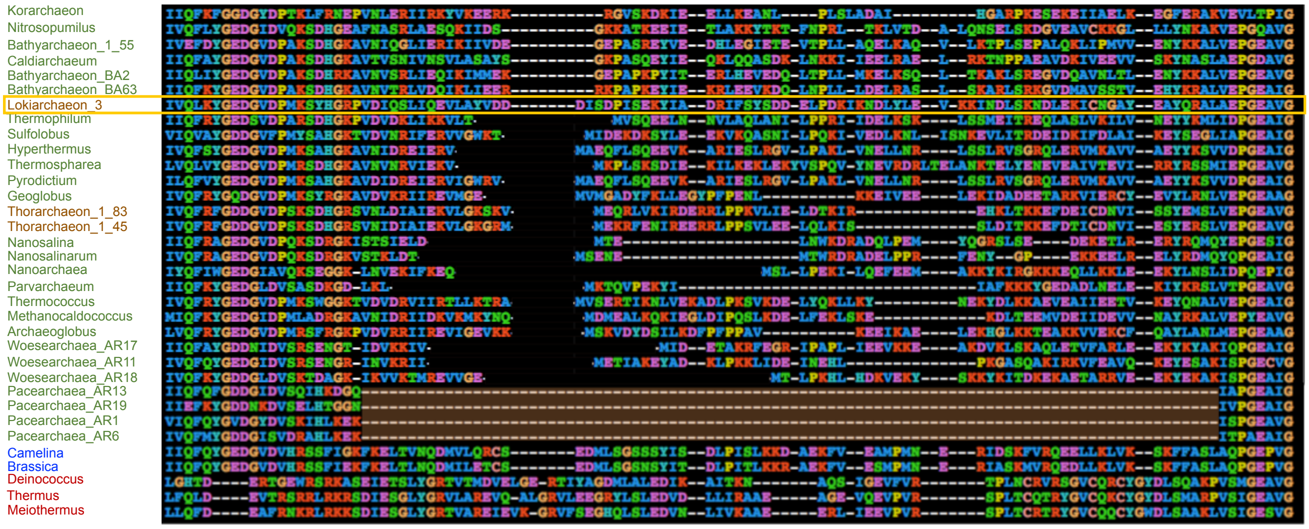

S29 Fig – Alignment of the region corresponding to the split in the RNA polymerase subunit A protein sequence.

Organisms' name corresponding to Bacteria, Lokiarchaea/Thorarchaea, Archaea, and Eukarya are respectively indicated in red, brown, green, and blue.
