# Supplementary material for: Lokiarchaea are close relatives of Euryarchaeota, not bridging the gap between prokaryotes and eukaryotes
Source: PLoS Genet. 2017 Jun 12;13(6):e1006810. doi: 10.1371/journal.pgen.1006810 (PMC5484517; doi:10.1371/journal.pgen.1006810)
Supplement: S33 Fig — a and b. ML phylogenetic trees of the concatenation of the two largest RNA polymerase subunits, using Bacteria as outgroup (1,670 positions) (a) or Eukaryotes (bacterial sequences removed; 2,175 positions) (b). Detailed trees in S34 and S35 Figs. Values at nodes indicate support calculated by nonparametric bootstrap (out of 100). The scale-bars represent the average number of substitutions per site. (PDF) [file pgen.1006810.s033.pdf]

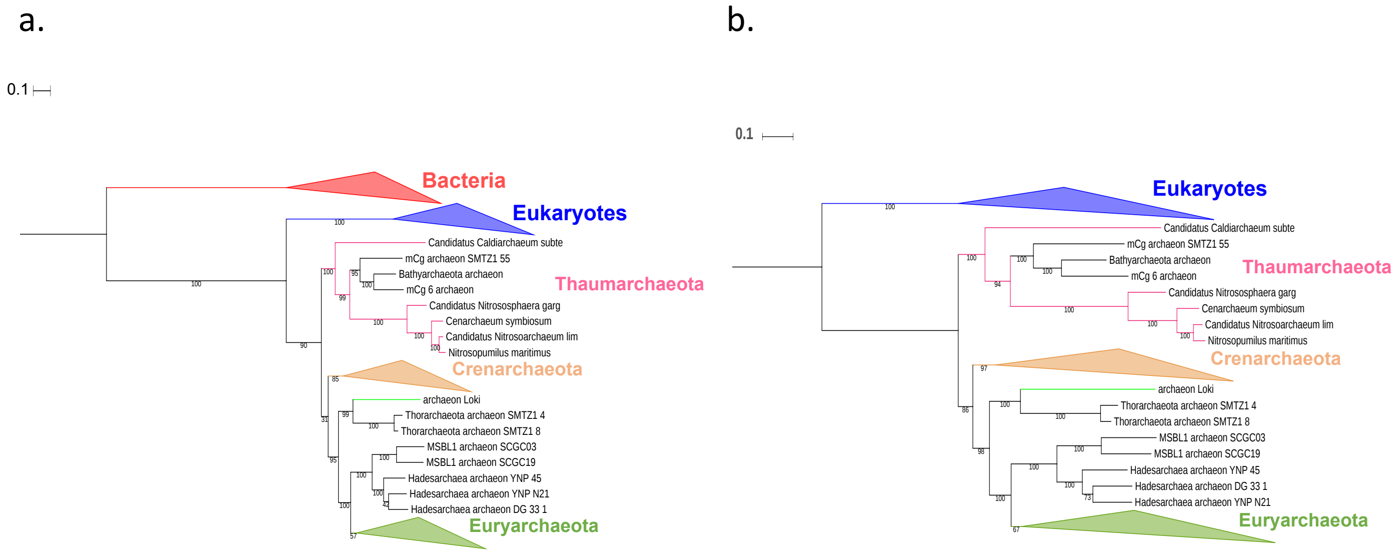

S33 Fig – Positions of Bathyarchaeota, Thorarchaeota, Hadesarchaeota and candidate division MSBL1 archaea based on the concatenation of the two largest RNA polymerase subunits on the new dataset.

a and b. ML phylogenetic trees of the concatenation of the two largest RNA polymerase subunits, using Bacteria as outgroup (1,670 positions) (**a**) or Eukaryotes (bacterial sequences removed; 2,175 positions) (**b**). Detailed trees in S34-S35 Figs. Values at nodes indicate support calculated by nonparametric bootstrap (out of 100). The scale-bars represent the average number of substitutions per site.
