# Supplementary material for: Lokiarchaea are close relatives of Euryarchaeota, not bridging the gap between prokaryotes and eukaryotes
Source: PLoS Genet. 2017 Jun 12;13(6):e1006810. doi: 10.1371/journal.pgen.1006810 (PMC5484517; doi:10.1371/journal.pgen.1006810)
Supplement: S35 Fig — In this tree, eukaryotic sequences are indicated in blue, and are used as outgroup. For Archaea, Thaumarchaeota and Aigarchaeota are indicated in pink, Crenarchaeota in orange and Euryarchaeota in olive-green. The scale-bar represents the average number of substitutions per site. Values at nodes represent support calculated by nonparametric bootstrap (out of 100). (PDF) [file pgen.1006810.s035.pdf]

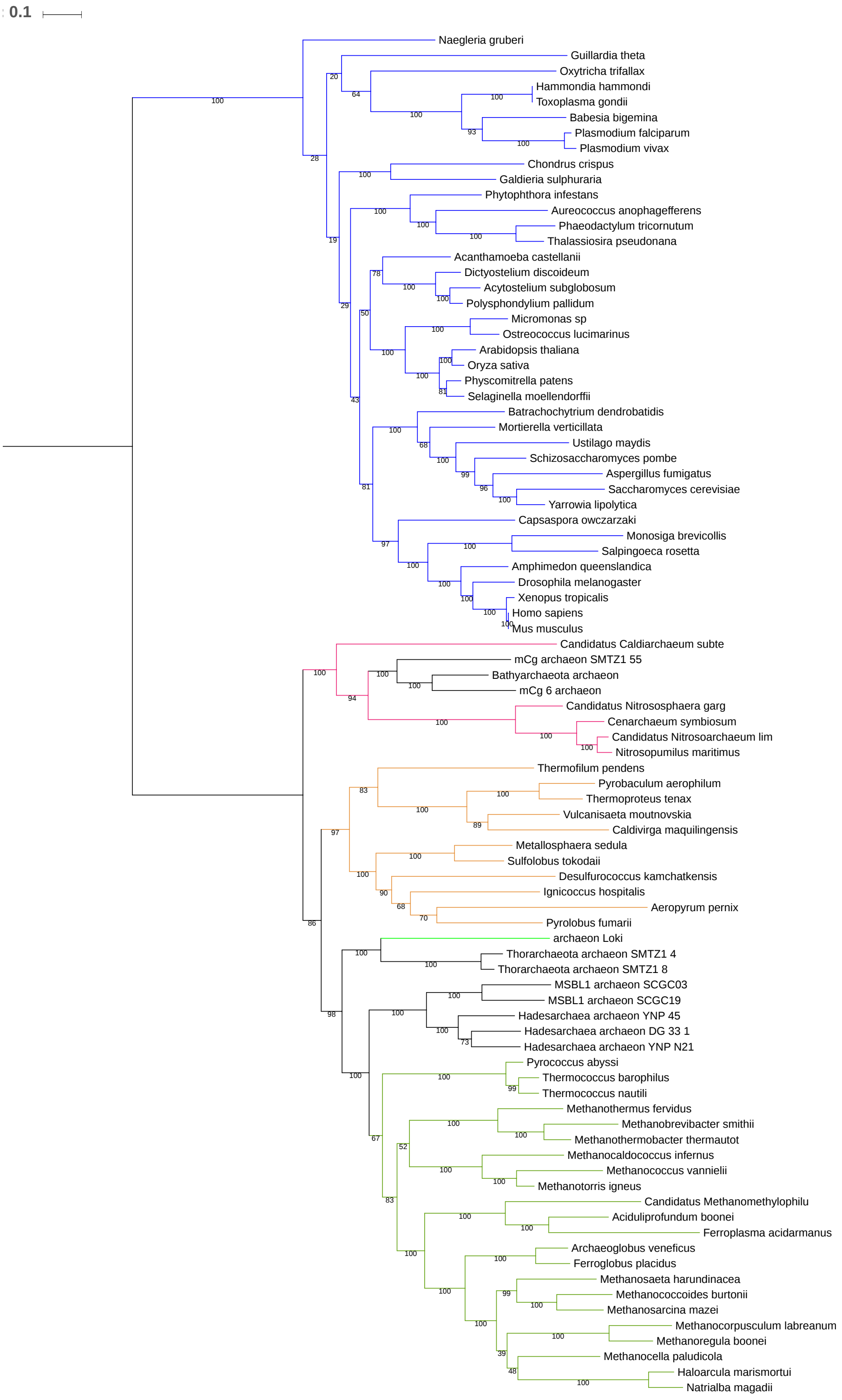

S35 Fig – ML phylogenetic tree of the concatenation of the two largest RNA polymerase subunits with the new dataset after inclusion of Bathyarchaeota, Thorarchaeota, Hadesarchaeota, and candidate division MSBL1 archaea, and removal of bacterial sequences.

In this tree, eukaryotic sequences are indicated in blue, and are used as outgroup. For Archaea, Thaumarchaeota and Aigarchaeota are indicated in pink, Crenarchaeota in orange and Euryarchaeota in olive-green. The scale-bar represents the average number of substitutions per site. Values at nodes represent support calculated by nonparametric bootstrap (out of 100).
