# Supplementary material for: Lokiarchaea are close relatives of Euryarchaeota, not bridging the gap between prokaryotes and eukaryotes
Source: PLoS Genet. 2017 Jun 12;13(6):e1006810. doi: 10.1371/journal.pgen.1006810 (PMC5484517; doi:10.1371/journal.pgen.1006810)
Supplement: S38 Fig — a. Alignments of the regions corresponding to two indels located on the RNA polymerase subunit A (on the left, starting position around 750, on the right around 1200). b. Alignment of the region corresponding to the indel located at the end of the Kae1 protein, with archaeal and eukaryotic sequences. Organisms’ names corresponding to Archaea and Eukaryotes are indicated in black and blue, respectively. The archaea presenting an indel are indicated in pink. (PDF) [file pgen.1006810.s038.pdf]

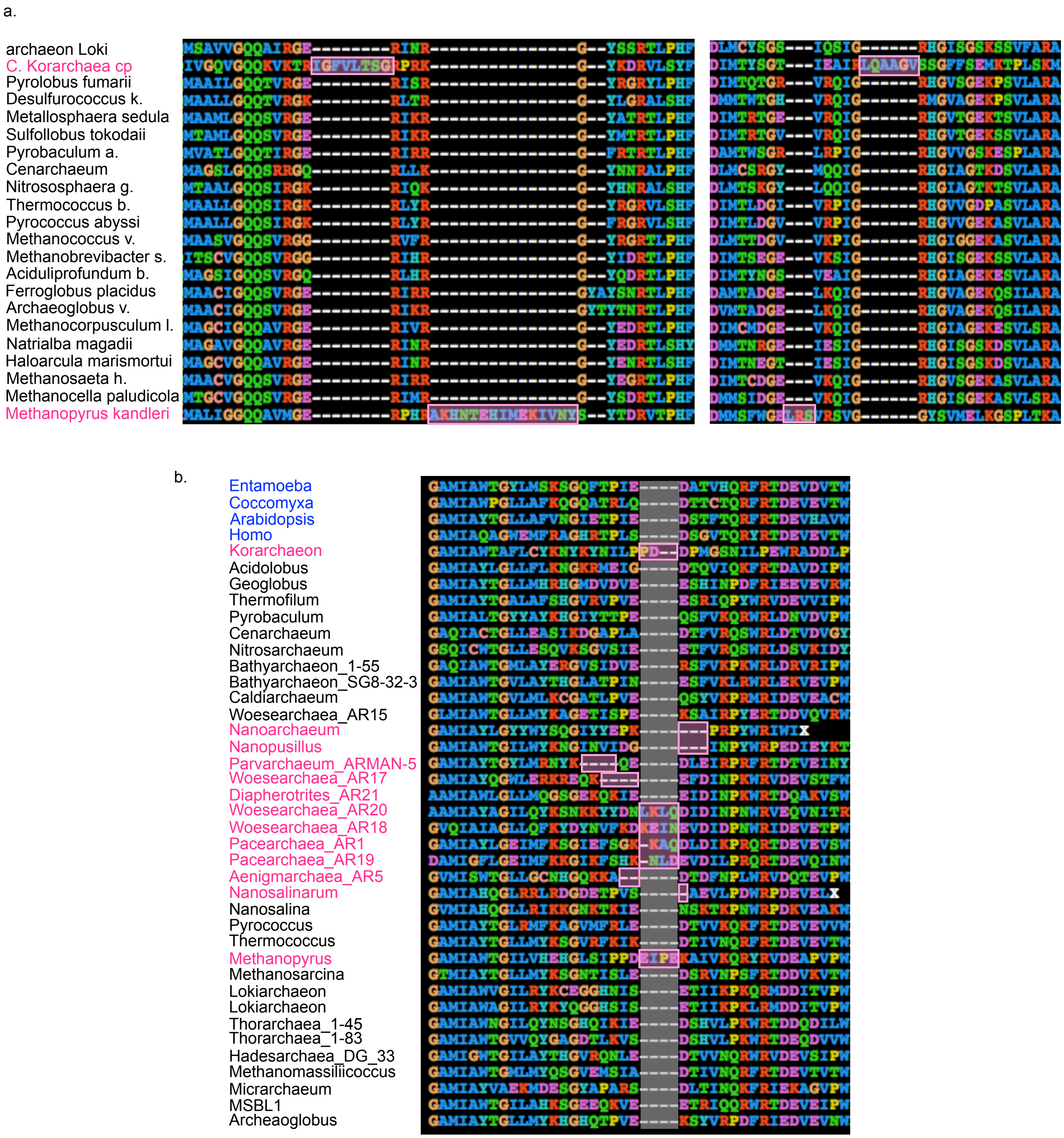

S38 Fig – Alignments of indels of *Candidatus* Korarchaeum cryptofilum and *Methanopyrus kandleri*. **a.** Alignments of the regions corresponding to two indels located on the RNA polymerase subunit A (on the left, starting position around 750, on the right around 1200). **b.** Alignment of the region corresponding to the indel located at the end of the Kae1 protein, with archaeal and eukaryotic sequences. Organisms' names corresponding to Archaea and Eukaryotes are indicated in black and blue, respectively. The archaea presenting an indel are indicated in pink.
