# Supplementary material for: Lokiarchaea are close relatives of Euryarchaeota, not bridging the gap between prokaryotes and eukaryotes
Source: PLoS Genet. 2017 Jun 12;13(6):e1006810. doi: 10.1371/journal.pgen.1006810 (PMC5484517; doi:10.1371/journal.pgen.1006810)
Supplement: S39 Fig — The gene encoding the RNA polymerase subunit is colored in light green. a. Comparison of the two Loki 1 contigs encoding the RNA polymerase B gene, and to their related contigs in the metagenome assembly. The names of the contigs corresponding to the metagenome are indicated in purple and those corresponding to the Loki 1 genome are indicated in pink or green based on their position (Set 2 and Set 4, respectively) in the S12 Fig on the analysis of the quality of the Loki 1 genome. The identity percentage between the contigs by tBLASTx approaches is also indicated. b. The two pairs of graphs correspond to reads coverage of Loki 1 contig 29 and Loki Metagenome contig 946, across the SRR1555743 and SRR1555748 sequencing runs (abbreviated SRR743 and SRR748, and in light blue and mauve, respectively). In theses graphs, the grey bars represent the base frequencies of small nucleotide variants (SNVs) observed in the contigs. The comparison of theses two contigs showed an insertion of five putative genes in the loki 1 contig 29 compared to the loki metagenome contig 946. The values indicated over the red arrows correspond to the total number of mapped pair-end reads (SRR1555743 and SRR1555748 runs combined) that support the absence on the five putative genes insertion and those that support both sides of the insertion. (PDF) [file pgen.1006810.s039.pdf]

**S39 Fig – Comparison of the lokiarchaeal contigs encoding the RNA polymerase subunit B gene in the Loki Castle metagenome assembly and in the Loki 1 genome.**

The gene encoding the RNA polymerase subunit is colored in light green. **a.** Comparison of the two Loki 1 contigs encoding the RNA polymerase B gene, and to their related contigs in the metagenome assembly. The names of the contigs corresponding to the metagenome are indicated in purple and those corresponding to the Loki 1 genome are indicated in pink or green based on their position (Set 2 and Set 4, respectively) in the S12 Fig on the analysis of the quality of the Loki 1 genome. The identity percentage between the contigs by tBLASTx approaches is also indicated. **b.** The two pairs of graphs correspond to reads coverage of Loki 1 contig 29 and Loki Metagenome contig 946, across the SRR1555743 and SRR1555748 sequencing runs (abbreviated SRR743 and SRR748, and in light blue and mauve, respectively). In theses graphs, the grey bars represent the base frequencies of small nucleotide variants (SNVs) observed in the contigs. The comparison of theses two contigs showed an insertion of five putative genes in the loki 1 contig 29 compared to the loki metagenome contig 946. The values indicated over the red arrows correspond to the total number of mapped pair-end reads (SRR1555743 and SRR1555748 runs combined) that support the absence on the five putative genes insertion and those that support both sides of the insertion.

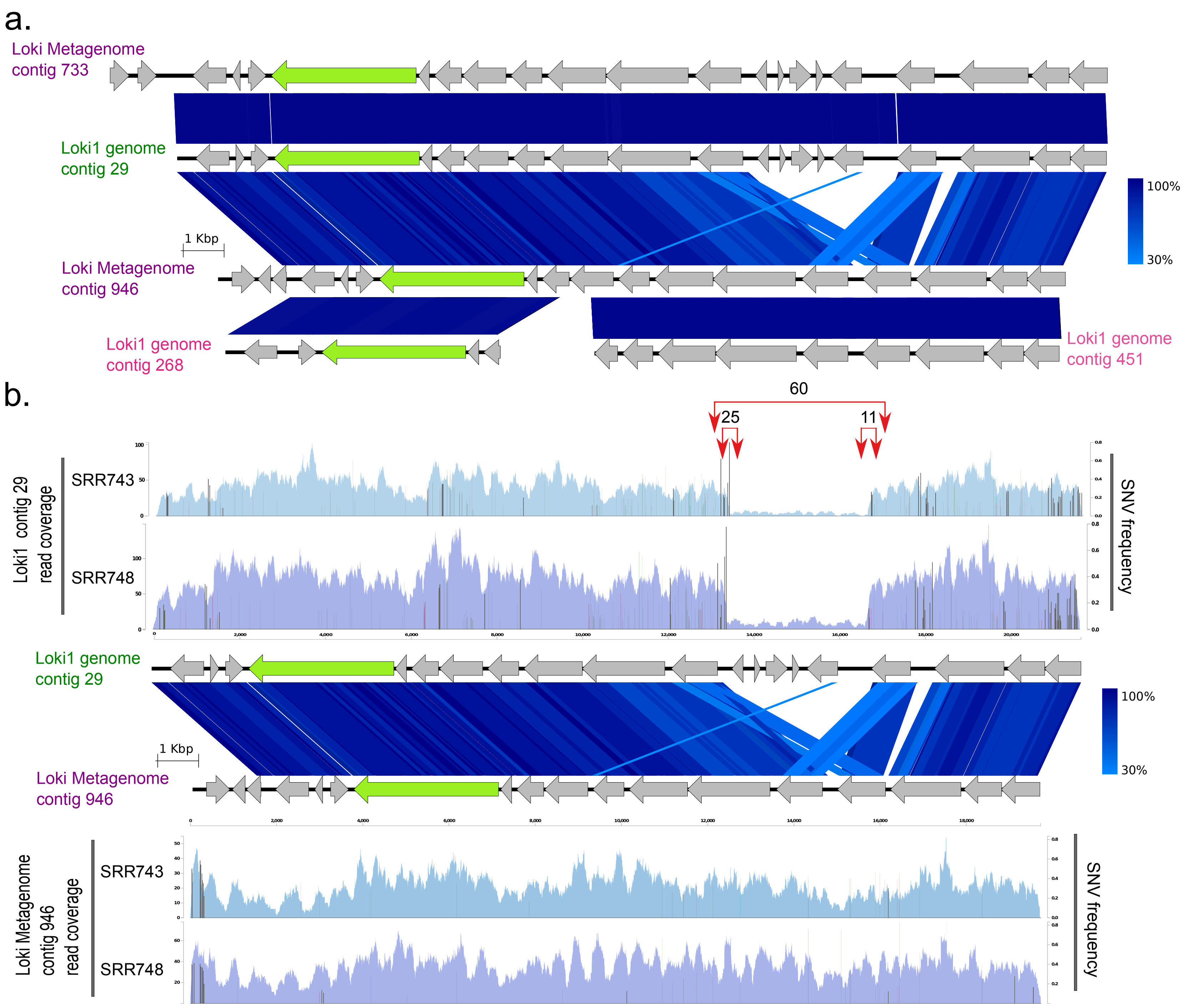
a.
Loki Metagenome contig 733
Loki1 genome contig 29
1 Kbp
Loki Metagenome contig 946
Loki1 genome contig 268
Loki1 genome contig 451
100%
30%
b.
60
25
11
Loki1 contig 29 read coverage
SRR743
SRR748
SNV frequency
Loki1 genome contig 29
1 Kbp
Loki Metagenome contig 946
100%
30%
Loki Metagenome contig 946 read coverage
SRR743
SRR748
SNV frequency
