# Supplementary material for: Lokiarchaea are close relatives of Euryarchaeota, not bridging the gap between prokaryotes and eukaryotes
Source: PLoS Genet. 2017 Jun 12;13(6):e1006810. doi: 10.1371/journal.pgen.1006810 (PMC5484517; doi:10.1371/journal.pgen.1006810)
Supplement: S1 Supporting information — The data related to the Loki 1 genome quality analysis can be interactively browsed through the included index.html file with an internet browser software. (ZIP) [file pgen.1006810.s041.zip › Supporting information 1/index.html]

Anvi'o: Alldata Loki Contigs MERGED


- Anvi'o Project Page
- Anvi'o Repository
- Report an Issue
- Contact
- Anvi'o version: 2.0.2


Bins that were identified in the  **single**  profile database '**Alldata Loki Contigs MERGED**' and stored in the
database as "**Set Collection tetra coverage Loki1**" collection, describe **6** bins accountig for
**5,140,761** nucleotides, which represent **99.95%**
of all nucleotides stored in the contigs database, and
**99.95%** of nucleotides stored in the profile database.

# Basics

The single profile database that was generated
with the minimum contig length of **500**
contained **504** contigs, which correspond to
**100%** of all contigs, and
**100%** of all nucleotides found
in the contigs database.

Profile DB for **Alldata Loki Contigs MERGED** w/ 3 samples.

| Key | Value |
| --- | --- |
| Created on | 2017-01-19 13:36:01 |
| Version | 16 |
| Minimum conting length | 500 |
| Number of contigs | 504 |
| Number of splits | 513 |
| Total nucleotides | 5.14 Mb |

Contigs DB

| Key | Value |
| --- | --- |
| Created on | 2017-01-19 13:36:01 |
| Version | 6 |
| Split length | 20,000 |
| Number of contigs | 504 |
| Number of splits | 513 |
| Total nucleotides | 5.14 Mb |
| K-mer size | 4 |

# Summary (6)

Summary of general characteristics of each bin (download as TAB-delimited file).

|  | Bin | Source | Taxonomy | Total Size | Num Contigs | N50 | GC Content | Compl. | Red. |
| --- | --- | --- | --- | --- | --- | --- | --- | --- | --- |
|  | Set\_4 | anvi-interactive | N/A | 1.86 Mb | 144 | 20,695 | 30.79% | 71.60% | 3.70% |
|  | Set\_2 | anvi-interactive | N/A | 1.06 Mb | 113 | 12,452 | 30.93% | 43.21% | 0.62% |
|  | Set\_6 | anvi-interactive | N/A | 1.60 Mb | 166 | 13,501 | 31.35% | 21.60% | 2.47% |
|  | Set\_5 | anvi-interactive | N/A | 558.20 Kb | 53 | 20,269 | 31.56% | 4.94% | 0.62% |
|  | Set\_3 | anvi-interactive | N/A | 22.26 Kb | 7 | 3,907 | 34.48% | 0.62% | 0.00% |
|  | Set\_1 | anvi-interactive | N/A | 33.66 Kb | 19 | 1,614 | 34.09% | 0.00% | 0.00% |

×

#### Taxonomy calls for Set 1

| Taxon call | Relative occurrence |
| --- | --- |

Taxonomy calls for splits in your 19 contigs

Close

×

#### Static files for Set 1

| File | Location |
| --- | --- |
| **Contigs FASTA** | bin\_by\_bin/Set\_1/Set\_1-contigs.fa |
| abundance | bin\_by\_bin/Set\_1/Set\_1-abundance.txt || N50 | bin\_by\_bin/Set\_1/Set\_1-N50.txt || mean\_coverage\_Q1Q3 | bin\_by\_bin/Set\_1/Set\_1-mean\_coverage\_Q1Q3.txt || variability | bin\_by\_bin/Set\_1/Set\_1-variability.txt || relative\_abundance | bin\_by\_bin/Set\_1/Set\_1-relative\_abundance.txt || original\_split\_names | bin\_by\_bin/Set\_1/Set\_1-original\_split\_names.txt || functions | bin\_by\_bin/Set\_1/Set\_1-functions.txt || Rinke\_archaeal\_HMM | bin\_by\_bin/Set\_1/Set\_1-Rinke\_archaeal\_HMM-hmm-sequences.txt || num\_contigs | bin\_by\_bin/Set\_1/Set\_1-num\_contigs.txt || portion\_covered | bin\_by\_bin/Set\_1/Set\_1-portion\_covered.txt || percent\_redundancy | bin\_by\_bin/Set\_1/Set\_1-percent\_redundancy.txt || max\_normalized\_ratio | bin\_by\_bin/Set\_1/Set\_1-max\_normalized\_ratio.txt || total\_length | bin\_by\_bin/Set\_1/Set\_1-total\_length.txt || GC\_content | bin\_by\_bin/Set\_1/Set\_1-GC\_content.txt || percent\_complete | bin\_by\_bin/Set\_1/Set\_1-percent\_complete.txt || std\_coverage | bin\_by\_bin/Set\_1/Set\_1-std\_coverage.txt || mean\_coverage | bin\_by\_bin/Set\_1/Set\_1-mean\_coverage.txt |

Files that were generated for your bin.

Close

×

#### Completeness estimations for "Set 1"

| Source | Percent completeness | Percent redundancy |
| --- | --- | --- |
| Rinke archaeal HMM | 0.00% | 0.00% |

See the documentation for more information on single-copy gene data.

Close

×

#### Taxonomy calls for Set 2

| Taxon call | Relative occurrence |
| --- | --- |

Taxonomy calls for splits in your 113 contigs

Close

×

#### Static files for Set 2

| File | Location |
| --- | --- |
| **Contigs FASTA** | bin\_by\_bin/Set\_2/Set\_2-contigs.fa |
| abundance | bin\_by\_bin/Set\_2/Set\_2-abundance.txt || N50 | bin\_by\_bin/Set\_2/Set\_2-N50.txt || mean\_coverage\_Q1Q3 | bin\_by\_bin/Set\_2/Set\_2-mean\_coverage\_Q1Q3.txt || variability | bin\_by\_bin/Set\_2/Set\_2-variability.txt || relative\_abundance | bin\_by\_bin/Set\_2/Set\_2-relative\_abundance.txt || original\_split\_names | bin\_by\_bin/Set\_2/Set\_2-original\_split\_names.txt || functions | bin\_by\_bin/Set\_2/Set\_2-functions.txt || Rinke\_archaeal\_HMM | bin\_by\_bin/Set\_2/Set\_2-Rinke\_archaeal\_HMM-hmm-sequences.txt || num\_contigs | bin\_by\_bin/Set\_2/Set\_2-num\_contigs.txt || portion\_covered | bin\_by\_bin/Set\_2/Set\_2-portion\_covered.txt || percent\_redundancy | bin\_by\_bin/Set\_2/Set\_2-percent\_redundancy.txt || max\_normalized\_ratio | bin\_by\_bin/Set\_2/Set\_2-max\_normalized\_ratio.txt || total\_length | bin\_by\_bin/Set\_2/Set\_2-total\_length.txt || GC\_content | bin\_by\_bin/Set\_2/Set\_2-GC\_content.txt || percent\_complete | bin\_by\_bin/Set\_2/Set\_2-percent\_complete.txt || std\_coverage | bin\_by\_bin/Set\_2/Set\_2-std\_coverage.txt || mean\_coverage | bin\_by\_bin/Set\_2/Set\_2-mean\_coverage.txt |

Files that were generated for your bin.

Close

×

#### Completeness estimations for "Set 2"

| Source | Percent completeness | Percent redundancy |
| --- | --- | --- |
| Rinke archaeal HMM | 43.21% | 0.62% |

See the documentation for more information on single-copy gene data.

Close

×

#### Taxonomy calls for Set 3

| Taxon call | Relative occurrence |
| --- | --- |

Taxonomy calls for splits in your 7 contigs

Close

×

#### Static files for Set 3

| File | Location |
| --- | --- |
| **Contigs FASTA** | bin\_by\_bin/Set\_3/Set\_3-contigs.fa |
| abundance | bin\_by\_bin/Set\_3/Set\_3-abundance.txt || N50 | bin\_by\_bin/Set\_3/Set\_3-N50.txt || mean\_coverage\_Q1Q3 | bin\_by\_bin/Set\_3/Set\_3-mean\_coverage\_Q1Q3.txt || variability | bin\_by\_bin/Set\_3/Set\_3-variability.txt || relative\_abundance | bin\_by\_bin/Set\_3/Set\_3-relative\_abundance.txt || original\_split\_names | bin\_by\_bin/Set\_3/Set\_3-original\_split\_names.txt || functions | bin\_by\_bin/Set\_3/Set\_3-functions.txt || Rinke\_archaeal\_HMM | bin\_by\_bin/Set\_3/Set\_3-Rinke\_archaeal\_HMM-hmm-sequences.txt || num\_contigs | bin\_by\_bin/Set\_3/Set\_3-num\_contigs.txt || portion\_covered | bin\_by\_bin/Set\_3/Set\_3-portion\_covered.txt || percent\_redundancy | bin\_by\_bin/Set\_3/Set\_3-percent\_redundancy.txt || max\_normalized\_ratio | bin\_by\_bin/Set\_3/Set\_3-max\_normalized\_ratio.txt || total\_length | bin\_by\_bin/Set\_3/Set\_3-total\_length.txt || GC\_content | bin\_by\_bin/Set\_3/Set\_3-GC\_content.txt || percent\_complete | bin\_by\_bin/Set\_3/Set\_3-percent\_complete.txt || std\_coverage | bin\_by\_bin/Set\_3/Set\_3-std\_coverage.txt || mean\_coverage | bin\_by\_bin/Set\_3/Set\_3-mean\_coverage.txt |

Files that were generated for your bin.

Close

×

#### Completeness estimations for "Set 3"

| Source | Percent completeness | Percent redundancy |
| --- | --- | --- |
| Rinke archaeal HMM | 0.62% | 0.00% |

See the documentation for more information on single-copy gene data.

Close

×

#### Taxonomy calls for Set 4

| Taxon call | Relative occurrence |
| --- | --- |

Taxonomy calls for splits in your 144 contigs

Close

×

#### Static files for Set 4

| File | Location |
| --- | --- |
| **Contigs FASTA** | bin\_by\_bin/Set\_4/Set\_4-contigs.fa |
| abundance | bin\_by\_bin/Set\_4/Set\_4-abundance.txt || N50 | bin\_by\_bin/Set\_4/Set\_4-N50.txt || mean\_coverage\_Q1Q3 | bin\_by\_bin/Set\_4/Set\_4-mean\_coverage\_Q1Q3.txt || variability | bin\_by\_bin/Set\_4/Set\_4-variability.txt || relative\_abundance | bin\_by\_bin/Set\_4/Set\_4-relative\_abundance.txt || original\_split\_names | bin\_by\_bin/Set\_4/Set\_4-original\_split\_names.txt || functions | bin\_by\_bin/Set\_4/Set\_4-functions.txt || Rinke\_archaeal\_HMM | bin\_by\_bin/Set\_4/Set\_4-Rinke\_archaeal\_HMM-hmm-sequences.txt || num\_contigs | bin\_by\_bin/Set\_4/Set\_4-num\_contigs.txt || portion\_covered | bin\_by\_bin/Set\_4/Set\_4-portion\_covered.txt || percent\_redundancy | bin\_by\_bin/Set\_4/Set\_4-percent\_redundancy.txt || max\_normalized\_ratio | bin\_by\_bin/Set\_4/Set\_4-max\_normalized\_ratio.txt || total\_length | bin\_by\_bin/Set\_4/Set\_4-total\_length.txt || GC\_content | bin\_by\_bin/Set\_4/Set\_4-GC\_content.txt || percent\_complete | bin\_by\_bin/Set\_4/Set\_4-percent\_complete.txt || std\_coverage | bin\_by\_bin/Set\_4/Set\_4-std\_coverage.txt || mean\_coverage | bin\_by\_bin/Set\_4/Set\_4-mean\_coverage.txt |

Files that were generated for your bin.

Close

×

#### Completeness estimations for "Set 4"

| Source | Percent completeness | Percent redundancy |
| --- | --- | --- |
| Rinke archaeal HMM | 71.60% | 3.70% |

See the documentation for more information on single-copy gene data.

Close

×

#### Taxonomy calls for Set 5

| Taxon call | Relative occurrence |
| --- | --- |

Taxonomy calls for splits in your 53 contigs

Close

×

#### Static files for Set 5

| File | Location |
| --- | --- |
| **Contigs FASTA** | bin\_by\_bin/Set\_5/Set\_5-contigs.fa |
| abundance | bin\_by\_bin/Set\_5/Set\_5-abundance.txt || N50 | bin\_by\_bin/Set\_5/Set\_5-N50.txt || mean\_coverage\_Q1Q3 | bin\_by\_bin/Set\_5/Set\_5-mean\_coverage\_Q1Q3.txt || variability | bin\_by\_bin/Set\_5/Set\_5-variability.txt || relative\_abundance | bin\_by\_bin/Set\_5/Set\_5-relative\_abundance.txt || original\_split\_names | bin\_by\_bin/Set\_5/Set\_5-original\_split\_names.txt || functions | bin\_by\_bin/Set\_5/Set\_5-functions.txt || Rinke\_archaeal\_HMM | bin\_by\_bin/Set\_5/Set\_5-Rinke\_archaeal\_HMM-hmm-sequences.txt || num\_contigs | bin\_by\_bin/Set\_5/Set\_5-num\_contigs.txt || portion\_covered | bin\_by\_bin/Set\_5/Set\_5-portion\_covered.txt || percent\_redundancy | bin\_by\_bin/Set\_5/Set\_5-percent\_redundancy.txt || max\_normalized\_ratio | bin\_by\_bin/Set\_5/Set\_5-max\_normalized\_ratio.txt || total\_length | bin\_by\_bin/Set\_5/Set\_5-total\_length.txt || GC\_content | bin\_by\_bin/Set\_5/Set\_5-GC\_content.txt || percent\_complete | bin\_by\_bin/Set\_5/Set\_5-percent\_complete.txt || std\_coverage | bin\_by\_bin/Set\_5/Set\_5-std\_coverage.txt || mean\_coverage | bin\_by\_bin/Set\_5/Set\_5-mean\_coverage.txt |

Files that were generated for your bin.

Close

×

#### Completeness estimations for "Set 5"

| Source | Percent completeness | Percent redundancy |
| --- | --- | --- |
| Rinke archaeal HMM | 4.94% | 0.62% |

See the documentation for more information on single-copy gene data.

Close

×

#### Taxonomy calls for Set 6

| Taxon call | Relative occurrence |
| --- | --- |

Taxonomy calls for splits in your 166 contigs

Close

×

#### Static files for Set 6

| File | Location |
| --- | --- |
| **Contigs FASTA** | bin\_by\_bin/Set\_6/Set\_6-contigs.fa |
| abundance | bin\_by\_bin/Set\_6/Set\_6-abundance.txt || N50 | bin\_by\_bin/Set\_6/Set\_6-N50.txt || mean\_coverage\_Q1Q3 | bin\_by\_bin/Set\_6/Set\_6-mean\_coverage\_Q1Q3.txt || variability | bin\_by\_bin/Set\_6/Set\_6-variability.txt || relative\_abundance | bin\_by\_bin/Set\_6/Set\_6-relative\_abundance.txt || original\_split\_names | bin\_by\_bin/Set\_6/Set\_6-original\_split\_names.txt || functions | bin\_by\_bin/Set\_6/Set\_6-functions.txt || Rinke\_archaeal\_HMM | bin\_by\_bin/Set\_6/Set\_6-Rinke\_archaeal\_HMM-hmm-sequences.txt || num\_contigs | bin\_by\_bin/Set\_6/Set\_6-num\_contigs.txt || portion\_covered | bin\_by\_bin/Set\_6/Set\_6-portion\_covered.txt || percent\_redundancy | bin\_by\_bin/Set\_6/Set\_6-percent\_redundancy.txt || max\_normalized\_ratio | bin\_by\_bin/Set\_6/Set\_6-max\_normalized\_ratio.txt || total\_length | bin\_by\_bin/Set\_6/Set\_6-total\_length.txt || GC\_content | bin\_by\_bin/Set\_6/Set\_6-GC\_content.txt || percent\_complete | bin\_by\_bin/Set\_6/Set\_6-percent\_complete.txt || std\_coverage | bin\_by\_bin/Set\_6/Set\_6-std\_coverage.txt || mean\_coverage | bin\_by\_bin/Set\_6/Set\_6-mean\_coverage.txt |

Files that were generated for your bin.

Close

×

#### Completeness estimations for "Set 6"

| Source | Percent completeness | Percent redundancy |
| --- | --- | --- |
| Rinke archaeal HMM | 21.60% | 2.47% |

See the documentation for more information on single-copy gene data.

Close

# Across Samples (3)

- abundance
- variability
- relative abundance
- mean coverage Q1Q3
- portion covered
- mean coverage
- max normalized ratio
- std coverage

TAB-delimited matrix file for abundance: bins\_across\_samples/abundance.txt

|  | Bin | SRR74 ... | SRR74 ... | SRR75 ... |
| --- | --- | --- | --- | --- |
|  | Set\_4 | 1.12 | 1.12 | 1.16 |
|  | Set\_2 | 0.57 | 0.57 | 0.67 |
|  | Set\_6 | 0.93 | 0.93 | 0.82 |
|  | Set\_5 | 1.36 | 1.36 | 1.55 |
|  | Set\_3 | 1.82 | 1.67 | 0.88 |
|  | Set\_1 | 2.02 | 1.92 | 2.40 |

TAB-delimited matrix file for variability: bins\_across\_samples/variability.txt

|  | Bin | SRR74 ... | SRR74 ... | SRR75 ... |
| --- | --- | --- | --- | --- |
|  | Set\_4 | 36.95 | 42.94 | 18.66 |
|  | Set\_2 | 12.76 | 17.13 | 8.16 |
|  | Set\_6 | 36.76 | 44.27 | 14.93 |
|  | Set\_5 | 38.86 | 43.85 | 25.84 |
|  | Set\_3 | 78.10 | 81.55 | 33.89 |
|  | Set\_1 | 51.75 | 53.90 | 47.80 |

TAB-delimited matrix file for relative\_abundance: bins\_across\_samples/relative\_abundance.txt

|  | Bin | SRR74 ... | SRR74 ... | SRR75 ... |
| --- | --- | --- | --- | --- |
|  | Set\_4 | 0.33 | 0.33 | 0.34 |
|  | Set\_2 | 0.31 | 0.32 | 0.37 |
|  | Set\_6 | 0.35 | 0.35 | 0.31 |
|  | Set\_5 | 0.32 | 0.32 | 0.36 |
|  | Set\_3 | 0.42 | 0.39 | 0.18 |
|  | Set\_1 | 0.32 | 0.31 | 0.38 |

TAB-delimited matrix file for mean\_coverage\_Q1Q3: bins\_across\_samples/mean\_coverage\_Q1Q3.txt

|  | Bin | SRR74 ... | SRR74 ... | SRR75 ... |
| --- | --- | --- | --- | --- |
|  | Set\_4 | 41.05 | 68.78 | 47.87 |
|  | Set\_2 | 20.17 | 33.67 | 26.75 |
|  | Set\_6 | 32.96 | 54.71 | 32.38 |
|  | Set\_5 | 49.88 | 83.48 | 64.30 |
|  | Set\_3 | 69.40 | 106.76 | 33.63 |
|  | Set\_1 | 79.11 | 124.25 | 102.87 |

TAB-delimited matrix file for portion\_covered: bins\_across\_samples/portion\_covered.txt

|  | Bin | SRR74 ... | SRR74 ... | SRR75 ... |
| --- | --- | --- | --- | --- |
|  | Set\_4 | 1.00 | 1.00 | 1.00 |
|  | Set\_2 | 1.00 | 1.00 | 0.99 |
|  | Set\_6 | 1.00 | 1.00 | 0.99 |
|  | Set\_5 | 1.00 | 1.00 | 1.00 |
|  | Set\_3 | 1.00 | 1.00 | 1.00 |
|  | Set\_1 | 1.00 | 1.00 | 1.00 |

TAB-delimited matrix file for mean\_coverage: bins\_across\_samples/mean\_coverage.txt

|  | Bin | SRR74 ... | SRR74 ... | SRR75 ... |
| --- | --- | --- | --- | --- |
|  | Set\_4 | 42.18 | 70.07 | 47.88 |
|  | Set\_2 | 21.55 | 35.65 | 27.62 |
|  | Set\_6 | 35.27 | 57.96 | 33.78 |
|  | Set\_5 | 51.41 | 85.00 | 64.02 |
|  | Set\_3 | 68.61 | 103.93 | 36.24 |
|  | Set\_1 | 76.20 | 119.64 | 99.57 |

TAB-delimited matrix file for max\_normalized\_ratio: bins\_across\_samples/max\_normalized\_ratio.txt

|  | Bin | SRR74 ... | SRR74 ... | SRR75 ... |
| --- | --- | --- | --- | --- |
|  | Set\_4 | 0.90 | 0.92 | 0.94 |
|  | Set\_2 | 0.83 | 0.84 | 0.99 |
|  | Set\_6 | 0.94 | 0.94 | 0.84 |
|  | Set\_5 | 0.86 | 0.87 | 0.99 |
|  | Set\_3 | 1.00 | 0.93 | 0.44 |
|  | Set\_1 | 0.82 | 0.79 | 0.97 |

TAB-delimited matrix file for std\_coverage: bins\_across\_samples/std\_coverage.txt

|  | Bin | SRR74 ... | SRR74 ... | SRR75 ... |
| --- | --- | --- | --- | --- |
|  | Set\_4 | 16.91 | 25.78 | 21.07 |
|  | Set\_2 | 10.26 | 15.64 | 12.62 |
|  | Set\_6 | 17.82 | 27.45 | 18.45 |
|  | Set\_5 | 18.31 | 27.74 | 23.41 |
|  | Set\_3 | 21.24 | 31.05 | 17.18 |
|  | Set\_1 | 22.55 | 35.02 | 31.51 |

# Percent Recruitment

This panel shows how much of the mapped data is recruited by each bin (and how much of the mapped data was not binned under 'splits\_not\_binned' column). The way these percents calculated is quite simple: summarize the mean coverage of each split in each bin, and normalize every bin with respect to each other. It is critical to remember that these values do not take the unasssambled data into account. This is how you should read this table: *"X percent of all mapped reads in Sample Y mapped to splits that were binned into bin Z"*.

TAB-delimited matrix file for percent recruitment: bins\_across\_samples/bins\_percent\_recruitment.txt

| Sample | Set\_1 | Set\_2 | Set\_3 | Set\_4 | Set\_5 | Set\_6 | \_\_splits\_not\_binned\_\_ |
| --- | --- | --- | --- | --- | --- | --- | --- |
| SRR743\_BT2\_CONT\_LOKI\_INIT | 7.36% | 12.39% | 2.44% | 31.75% | 14.12% | 30.50% | 1.44% |
| SRR748\_BT2\_CONT\_LOKI\_INIT | 7.04% | 12.48% | 2.25% | 32.12% | 14.22% | 30.52% | 1.36% |
| SRR750\_BT2\_CONT\_LOKI\_INIT | 8.74% | 14.41% | 1.17% | 32.72% | 15.96% | 26.52% | 0.48% |

# Genes & Functions

An overview of genes and functions based on the information found in the contigs database. If you haven't assigned any functions, these tables will only contain coverages of ORFs across samples.

|  | Bin | Total Size | Num Genes Identified | Tabular data |
| --- | --- | --- | --- | --- |
|  | Set\_4 | 1.86 Mb | 1,939 | 🔗 Link |
|  | Set\_2 | 1.06 Mb | 1,124 | 🔗 Link |
|  | Set\_6 | 1.60 Mb | 1,667 | 🔗 Link |
|  | Set\_5 | 558.20 Kb | 524 | 🔗 Link |
|  | Set\_3 | 22.26 Kb | 22 | 🔗 Link |
|  | Set\_1 | 33.66 Kb | 35 | 🔗 Link |

### Hidden Markov Model Hits (none found)
