# Supplementary material for: Lokiarchaea are close relatives of Euryarchaeota, not bridging the gap between prokaryotes and eukaryotes
Source: PLoS Genet. 2017 Jun 12;13(6):e1006810. doi: 10.1371/journal.pgen.1006810 (PMC5484517; doi:10.1371/journal.pgen.1006810)
Supplement: S1 Supporting information — The data related to the Loki 1 genome quality analysis can be interactively browsed through the included index.html file with an internet browser software. (ZIP) [file pgen.1006810.s041.zip › Supporting information 1/.html/chartsjs/samples/line.html]

Line Chart
Randomize Data
