# Supplementary material for: Lokiarchaea are close relatives of Euryarchaeota, not bridging the gap between prokaryotes and eukaryotes
Source: PLoS Genet. 2017 Jun 12;13(6):e1006810. doi: 10.1371/journal.pgen.1006810 (PMC5484517; doi:10.1371/journal.pgen.1006810)
Supplement: S1 Table — (PDF) [file pgen.1006810.s042.pdf]

S1 Table – Comparative analysis of the 36 universal proteins phylogenetic trees obtained with the initial dataset (ID) and the FES-curated dataset (CD).

			Monophyly of the 3 major archaeal phyla (bootstrap value)						Paraphyly vs. Monophyly of Archaea (bootstrap value)			
			Euryarchaeota		Crenarchaeota		Thaumarchaeota		Archaeal paraphyly, with sister group to Eukarya		Archaeal monophyly	
**arCOG**	**name**	**Size[a]**	**ID**	**CD**	**ID**	**CD**	**ID**	**CD**	**ID**	**CD**	**ID**	**CD**
01762	RPB	899/910	+ (4)	+ (96)	+ (64)	+ (87)	+ (48)	+ (97)	*Korarchaeon* (47)	-	-	+ (100)
04257	RPA'	675/726	-	+ (90)	+ (19)	+ (84)	+ (66)	+ (61)	*Korarchaeon* (60)	-	-	+ (100)
01559	EFG	626/643	+ (46)	+ (96)	+ (81)	+ (76)	+ (99)	+ (100)	Loki 3 (100) Loki 1 Loki 2 (75)*	Loki 3 (100) Loki 1 Loki 2 (66)*	-	-
01560	IF2	499/502	-	+ (45)	+ (11)	+ (51)	-	+ (18)	Loki 2 (30)*	Loki 2 (57)*	-	-
01228	SRP	365/370	-	-	+ (10)	+ (73)	+ (18)	+ (18)	wA (1)	wA (26)	-	-
04169	SecY	363/363	-	+ (59)	+ (75)	-	+ (34)	+ (57)	Loki 1, Loki 3 (84)*	Loki 1, Loki 3 (62)*	-	-
00412	Phe tRNA S	329/332	-	-	+ (19)	+ (46)	+ (55)	+ (73)	wA (8)	-	-	+ (100)
00415	RecA	279/285	-	+ (19)	-	-	+ (98)	+	wA (52)	-	-	+ (100)
01183	Kae1/YgjD	277/264	-	-	+ (20)	+ (35)	-	-	Loki 2 (59)*	Loki 2 (74)*	-	-
00987	Pseudo US	251/257	-	-	-	-	-	-	Thermofilum (32)	Thermofilum (28)	-	-
04256	RPA''	243/281	-	+ (55)	-	+ (4)	-	+ (47)	*Methanopyrus*/A (37)	-	-	+ (100)
01227	SRP	233/250	-	-	-	-	-	-	wA (1)	Loki 1, Loki 3 (8)*	-	-
04254	RPS7	178/178	-	+ (57)	-	-	+ (49)	+ (49)	*Korarchaeon* (47)	-	-	+ (100)
04289	RPL1	175/191	+ (0)	+ (38)	-	-	-	-	-	-	+ (100)	+ (100)
04245	RPS2	168/175	-	+ (78)	+ (2)	-	+ (14)	-	Caldiarchaeum (17)	-	-	+ (100)
04064	Zn protease	165/184	-	-	-	+ (13)	+ (10)	+ (25)	*Nano* (22)	wA (13)	-	
04097	RPS3	160/174	-	+ (67)	-	+ (63)	+ (34)	+ (36)	Loki 1, Loki 3 (25)*	Loki 1, Loki 3 (25)*	-	+ (100)[b]
04092	RPL5	152/159	-	-	-	+ (11)	-	-	Geaoarchaea (31)	Loki 3 (20)*	-	-
04241	RPD	142/149	-	-	-	-	-	+ (67)	Bacteria/Archaea (100/20)	-	-	+ (100)
04090	RPL6	142/142	-	-	-	+ (53)	-	+ (22)	wA(0)	Loki 3 (32)*	-	-
04255	RPS12	133/135	+ (0)	+ (17)	+ (14)	+ (34)	-	-	Loki 1 (11)*	wA (8)	-	-
01722	RPS13	131/131	-	-	-	-	-	-	wA (2)	wA (19)	-	-
04239	RPS4	129/146	-	+ (28)	+ (16)	+ (36)	-	+ (5)	Caldiarchaeum/Geoarchaea (4)	Caldiarchaeum (14)	-	-
04113	RPL10/16	126/140	-	+ (8)	-	-	+ (45)	+ (36)	Loki 3 (25) Loki 1 Loki 2 (7)*	Loki 1 (34)*	-	-
04240	RPS11	124/121	-	-	+ (55)	+ (67)	-	+ (67)	Loki 2 (46)*	Loki 2 (62)*	-	-
04091	RPS8	122/122	-	-	+ (4)	+ (8)	+ (53)	+ (57)	Loki 3 (40)*	Methanocellales (15)	-	-
04243	RPS9	120/119	+ (0)	+ (4)	-	+ (51)	-	+ (35)	wA (0)	Loki2 Loki 3 (16)*	-	-
04095	RPL14	118/126	-	-	-	-	+ (44)	+ (76)	*Korarchaeon* (11)	Thaumarchaeota (24)	-	-
04098	RPL22	116/123	-	-	-	-	-	-	*Korarchaeon, Micra* (47)	Thermoplasmatales (3)	-	-
04121	Rnase H II	115/113	-	-	-	-	-	-	wA (0)	Sulfolobales (13)	-	-
04099	RPS19	109/117	-	-	-	-	-	+ (70)	*Micra, Parva, Nano* (0)	wA (3)	-	-
04094	RPL24	107/103	-	-	-	-	-	-	*Nano* (24)	Loki 1 Loki 3 Acidilobus (4)	-	-
01758	RPS10	98/98	-	+ (36)	-	-	-	-	*Parva* (3)	-	-	+ (100)
04096	RPS17	93/92	-	-	+ (8)	+ (57)	-	-	Loki 1 (6)*	Thaumarchaeota (9)	-	-
04242	RPL13	88/87	-	-	-	-	-	-	wA (0)	Loki 1, Loki 2 (26)*	-	-
00785	RPL29	56/59	-	+ (3)	-	-	-	+ (11)	wA (3)	Acidilobus (9)	-	-

The analyses with the initial (ID) and curated datasets (CD) are respectively indicated within the white and grey columns.

[a] The indicated values correspond to the number of aligned amino acid positions obtained after trimming with the initial and curated datasets (initial/curated).

[b] With the curated arCOG04097 dataset (RPS3), Archaea appeared monophyletic except the two Loki sequences that were branching as sister group to Eukarya (see S2 Fig).

The * symbol indicates the cases in which at least one Loki sequence is sister group to Eukaryotes. Fast-evolving sequences are indicated in italic.

"wA" stand for "within Archaea" (no specific position), "Nano" for "Nanoarchaea", "Parva" for "Parvarchaea", "Micra" for "Micrarchaea".
