# Supplementary material for: Lokiarchaea are close relatives of Euryarchaeota, not bridging the gap between prokaryotes and eukaryotes
Source: PLoS Genet. 2017 Jun 12;13(6):e1006810. doi: 10.1371/journal.pgen.1006810 (PMC5484517; doi:10.1371/journal.pgen.1006810)
Supplement: S2 Table — (PDF) [file pgen.1006810.s043.pdf]

S2 Table – Results of Approximately Unbiased test with single protein alignments on the 11 or 25 proteins tree topology selection.

Single proteins		Alignment length Post-trimming	AU Test 11 proteins tree topology	AU Test 25 proteins tree topology
arCOG01762	RPB	910	0.9344 +	0.0656 +
**arCOG04257**	**RPA'**	**726**	**0.9810 +**	**0.0189 -**
**arCOG01559**	**EFG**	**643**	**0.0000 -**	**1.0000 +**
**arCOG01560**	**IF2**	**502**	**0.0419 -**	**0.9581 +**
**arCOG01228**	**SRP**	**370**	**0.0014 -**	**0.9986 +**
arCOG04169	SecY	363	0.1290 +	0.8710 +
**arCOG00412**	**Phe tRNA**	**332**	**0.9999 +**	**0.0001 -**
arCOG00415	RecA	285	0.6747 +	0.3653 +
**arCOG04256**	**RPA ''**	**281**	**0.9841 +**	**0.0157 -**
arCOG01183	Kae1/YgjD	264	0.1136 +	0.8864 +
arCOG00987	Pseudo US	257	0.3835 +	0.6165 +
**arCOG01227**	**SRP**	**250**	**0.0121 -**	**0.9878 +**
**arCOG04289**	**RPL1**	**191**	**0.9519 +**	**0.0481 -**
**arCOG04064**	**Zn protease**	**184**	**0.0376 -**	**0.9624 +**
**arCOG04254**	**RPS7**	**178**	**0.9895 +**	**0.0105 -**
arCOG04245	RPS2	175	0.7554 +	0.2446 +
arCOG04097	RPS3	174	0.8367 +	0.1633 +
**arCOG04092**	**RPL5**	**159**	**0.0202 -**	**0.9798 +**
**arCOG04241**	**RPD**	**149**	**0.9893 +**	**0.0107 -**
**arCOG04239**	**RPS4**	**146**	**0.0222 -**	**0.9778 +**
arCOG04090	RPL6	142	0.3588 +	0.6412 +
**arCOG04113**	**RPL10/16**	**140**	**0.0249 -**	**0.9751 +**
arCOG04255	RPS12	135	0.2418 +	0.7582 +
arCOG01722	RPS13	131	0.1999 +	0.8001 +
arCOG04095	RPL14	126	0.1169 +	0.8831 +
arCOG04098	RPL22	123	0.5119 +	0.4881 +
**arCOG04091**	**RPS8**	**122**	**0.0096 -**	**0.9904 +**
**arCOG04240**	**RPS11**	**121**	**0.0299 -**	**0.9701 +**
arCOG04243	RPS9	119	0.3267 +	0.6733 +
arCOG04099	RPS19	117	0.1447 +	0.8553 +
**arCOG04121**	**Rnase H II**	**113**	**0.0196 -**	**0.9804 +**
arCOG04094	RPL24	103	0.1023 +	0.8977 +
arCOG01758	RPS10	98	0.9165 +	0.0835 +
arCOG04096	RPS17	92	0.0966 +	0.9034 +
arCOG04242	RPL13	87	0.0710 +	0.9290 +
arCOG00785	RPL29	59	0.5640 +	0.4360 +

The + symbol indicates the 95% confidence sets. The - symbol indicates significant exclusion. Proteins from the Woese and the eocyte subsets are colored in red and black, respectively. The proteins that significantly support a topology (i.e. that also reject the other) are written in bold.
