# Supplementary material for: Lokiarchaea are close relatives of Euryarchaeota, not bridging the gap between prokaryotes and eukaryotes
Source: PLoS Genet. 2017 Jun 12;13(6):e1006810. doi: 10.1371/journal.pgen.1006810 (PMC5484517; doi:10.1371/journal.pgen.1006810)
Supplement: S3 Table — (PDF) [file pgen.1006810.s044.pdf]

S3 Table – Results of Approximately Unbiased test with 27 single protein alignments on the different single protein topologies.

	arCOG00412	arCOG01183	arCOG01227	arCOG01228	arCOG01559	arCOG01722	arCOG01758	arCOG01762	arCOG04090	arCOG04091	arCOG04092	arCOG04095	arCOG04096	arCOG04097	arCOG04098	arCOG04099	arCOG04113	arCOG04239	arCOG04240	arCOG04242	arCOG04243	arCOG04245	arCOG04254	arCOG04255	arCOG04256	arCOG04257	arCOG04289
arCOG00412	1.000	3E-50	2E-44	5E-07	9E-43	3E-09	5E-54	3E-11	1E-09	2E-63	1E-07	1E-64	2E-72	2E-37	2E-09	9E-80	2E-48	1E-50	3E-10	1E-37	3E-10	2E-14	4E-46	2E-55	4E-45	2E-50	3E-06
arCOG01183	3E-72	1.000	3E-06	4E-09	3E-05	4E-06	1E-04	3E-72	5E-07	4E-07	2E-09	1E-08	2E-54	6E-59	2E-46	2E-10	1E-91	7E-43	2E-73	1E-09	7E-74	3E-06	6E-97	2E-46	1E-31	3E-85	2E-61
arCOG01227	6E-07	8E-30	1.000	9E-36	2E-56	4E-05	4E-07	2E-56	1E-08	2E-06	6E-53	6E-07	1E-09	3E-103	2E-43	3E-05	6E-32	2E-09	3E-34	2E-05	3E-38	2E-05	2E-62	4E-06	2E-64	8E-08	2E-87
arCOG01228	4E-09	1E-09	1E-07	1.000	3E-48	2E-05	2E-04	3E-87	4E-09	1E-93	6E-06	1E-07	2E-53	1E-06	9E-06	3E-33	4E-104	6E-05	6E-05	1E-06	1E-05	5E-16	2E-07	4E-06	1E-05	2E-05	2E-09
arCOG01559	1E-12	3E-47	2E-06	2E-05	1.000	1E-05	4E-05	2E-06	6E-05	4E-06	5E-06	3E-07	1E-59	7E-07	9E-05	1E-05	4E-06	9E-06	0.001	8E-07	3E-11	2E-06	1E-06	3E-09	7E-57	2E-06	3E-43
arCOG01722	5E-24	2E-54	2E-06	8E-64	9E-38	1.000	4E-88	2E-12	1E-07	4E-93	8E-65	4E-07	2E-79	2E-09	2E-40	1E-08	3E-09	1E-06	5E-05	5E-45	1E-07	8E-06	3E-37	7E-66	2E-43	6E-14	4E-70
arCOG01758	5E-34	8E-29	6E-21	3E-164	2E-06	1E-46	1.000	1E-46	6E-07	6E-09	2E-51	4E-07	5E-33	8E-72	4E-54	8E-46	2E-06	2E-05	7E-07	4E-54	4E-84	3E-07	4E-108	7E-43	9E-09	1E-04	6E-06
arCOG01762	4E-65	2E-07	5E-05	2E-53	5E-59	3E-05	4E-05	1.000	1E-04	2E-33	5E-05	1E-05	1E-07	1E-05	8E-05	1E-06	1E-12	3E-07	3E-04	2E-05	1E-05	2E-04	2E-05	2E-05	4E-04	1E-04	2E-06
arCOG04090	4E-36	3E-08	4E-09	2E-39	4E-54	1E-12	6E-110	1E-10	1.000	6E-35	2E-52	4E-49	6E-47	1E-08	2E-32	3E-71	6E-60	3E-10	1E-60	1E-37	2E-08	5E-06	3E-57	7E-10	8E-72	1E-05	1E-05
arCOG04091	2E-05	2E-43	1E-47	3E-52	4E-45	9E-07	3E-41	1E-50	2E-50	1.000	2E-65	2E-47	1E-31	3E-06	7E-11	1E-06	2E-46	6E-101	1E-71	9E-51	1E-67	9E-44	6E-06	3E-99	7E-40	2E-36	4E-08
arCOG04092	6E-38	1E-08	1E-07	6E-57	2E-06	3E-60	7E-08	1E-28	9E-135	6E-05	1.000	4E-40	2E-07	2E-05	4E-37	9E-72	4E-53	4E-09	1E-04	1E-08	8E-76	2E-44	4E-78	2E-49	6E-07	4E-06	3E-25
arCOG04095	2E-60	1E-38	2E-126	9E-81	1E-12	3E-05	4E-04	2E-50	1E-51	3E-29	6E-40	1.000	7E-12	6E-06	6E-82	6E-09	6E-62	2E-89	6E-46	2E-07	2E-67	2E-05	4E-55	3E-80	2E-106	2E-08	1E-68
arCOG04096	2E-68	7E-05	2E-69	7E-06	2E-05	1E-87	3E-08	5E-35	3E-12	3E-88	4E-06	3E-35	1.000	1E-49	5E-08	8E-55	4E-68	5E-32	9E-74	1E-06	4E-14	2E-51	5E-46	2E-29	2E-06	6E-07	1E-06
arCOG04097	7E-67	2E-26	1E-38	1E-74	1E-68	8E-07	2E-04	5E-73	2E-05	2E-28	7E-06	4E-39	4E-06	1.000	3E-08	4E-06	9E-08	6E-44	2E-05	1E-05	3E-08	1E-41	1E-11	2E-06	1E-06	3E-56	2E-06
arCOG04098	1E-59	4E-105	3E-59	6E-107	6E-60	1E-59	4E-06	2E-07	3E-49	1E-57	4E-05	1E-96	1E-68	3E-07	1.000	9E-51	1E-65	9E-09	4E-07	3E-122	3E-67	6E-44	2E-41	1E-35	5E-91	1E-10	1E-45
arCOG04099	3E-27	7E-94	5E-05	0.001	1E-56	1E-05	1E-35	4E-86	8E-123	2E-110	1E-07	7E-15	2E-07	2E-22	6E-08	1.000	5E-09	1E-48	3E-06	5E-28	6E-08	4E-13	9E-49	2E-11	7E-08	3E-64	1E-46
arCOG04113	2E-10	6E-48	2E-52	9E-24	3E-12	8E-42	5E-05	4E-35	7E-40	3E-97	2E-94	7E-43	1E-45	3E-09	3E-08	2E-06	1.000	3E-50	1E-75	2E-10	9E-51	3E-05	2E-50	3E-83	8E-99	2E-05	3E-53
arCOG04239	3E-06	2E-09	4E-63	1E-26	2E-62	1E-39	7E-06	1E-54	2E-26	3E-07	2E-05	1E-58	2E-51	2E-43	6E-29	3E-08	6E-06	1.000	3E-05	4E-55	5E-06	1E-06	7E-103	1E-78	3E-07	2E-08	2E-08
arCOG04240	8E-75	4E-19	8E-05	2E-59	5E-54	7E-37	1E-19	1E-09	7E-08	6E-07	7E-12	6E-09	1E-06	2E-06	8E-92	5E-07	1E-46	4E-11	1.000	2E-66	2E-09	2E-06	9E-06	2E-25	7E-07	1E-07	1E-08
arCOG04242	3E-71	3E-06	2E-32	1E-06	6E-42	1E-48	4E-66	5E-06	1E-44	6E-06	2E-77	4E-35	4E-96	2E-05	4E-05	1E-25	2E-44	4E-72	6E-70	1.000	7E-37	1E-121	7E-71	8E-57	2E-58	4E-09	5E-60
arCOG04243	9E-92	3E-35	7E-06	3E-06	2E-04	3E-09	9E-08	4E-07	3E-13	2E-38	4E-28	1E-63	2E-38	2E-05	6E-06	3E-05	1E-09	1E-51	1E-09	5E-60	1.000	3E-06	3E-17	1E-05	7E-76	3E-07	2E-06
arCOG04245	2E-54	2E-06	3E-07	2E-73	3E-07	1E-08	8E-05	2E-80	6E-08	2E-10	8E-06	3E-63	3E-62	4E-10	1E-07	1E-14	5E-48	2E-68	6E-06	7E-06	3E-49	1.000	3E-07	4E-05	6E-130	3E-35	4E-78
arCOG04254	1E-49	1E-54	1E-11	2E-34	4E-07	2E-13	6E-07	5E-88	2E-08	5E-42	7E-65	9E-05	3E-11	1E-05	6E-06	3E-53	9E-07	1E-49	9E-56	2E-89	3E-06	1E-05	1.000	4E-08	9E-06	4E-69	2E-07
arCOG04255	6E-07	5E-62	3E-56	5E-77	0.001	3E-13	3E-55	5E-08	4E-07	7E-33	1E-50	9E-75	6E-07	8E-43	2E-05	1E-05	4E-41	9E-12	2E-58	1E-07	8E-66	1E-04	1E-05	1.000	8E-90	2E-08	5E-84
arCOG04256	1E-46	1E-09	1E-83	3E-72	6E-63	7E-08	6E-06	3E-86	3E-09	5E-47	2E-05	7E-10	3E-83	3E-46	2E-06	2E-106	2E-04	5E-38	2E-114	7E-46	2E-67	3E-08	5E-09	9E-66	1.000	9E-61	4E-06
arCOG04257	3E-47	3E-13	2E-12	4E-08	3E-22	4E-09	4E-04	3E-04	2E-05	2E-08	5E-06	1E-10	3E-58	8E-07	4E-07	4E-13	6E-06	2E-07	2E-04	2E-06	3E-05	4E-06	5E-06	5E-05	5E-05	1.000	6E-06
arCOG04289	6E-07	7E-13	8E-48	3E-11	1E-42	1E-55	3E-11	3E-38	1E-95	2E-80	8E-15	7E-22	7E-54	1E-46	1E-51	4E-05	2E-71	2E-08	8E-07	1E-91	5E-46	5E-08	1E-76	3E-50	1E-06	4E-57	1.000
