# Supplementary material for: Lokiarchaea are close relatives of Euryarchaeota, not bridging the gap between prokaryotes and eukaryotes
Source: PLoS Genet. 2017 Jun 12;13(6):e1006810. doi: 10.1371/journal.pgen.1006810 (PMC5484517; doi:10.1371/journal.pgen.1006810)
Supplement: S4 Table — (PDF) [file pgen.1006810.s045.pdf]

S4 Table – List of accession numbers of the Lokiarchaeal and Thorarchaeal proteins included in the concatenation-based analyses.

		Loki 1	Loki 2	Loki 3	Thor 1_83	Thor 1_45	Location of Loki1 protein in FigS12
arCOG00412	Phe tRNA	KKK44393.1		KP869700 (AKC94971.1)	KXH76274	KXH71486	Set_6
arCOG00415	RecA	KKK45633.1		KP869669 (AKC94940.1)	KXH75335	KXH71493	Set_4
arCOG00785	RPL29	KKK44315.1	KP869671 (AKC94942.1)	KP869704 (AKC94975.1)	KXH70452.1	KXH74610.1	Set_4
arCOG00987	Pseudo US	KKK46194.1	KP869689 (AKC94960.1)		KXH70431.1	KXH71150.1	Set_4
arCOG01183	Kae1/YgjD	KKK41557.1	KP869695 (AKC94966.1)	KP869721 (AKC94992.1)	KXH70364.1	KXH72633	Set_4
arCOG01227	SRP	KKK44958.1		KP869607 (AKC94879.1)	KXH77808.1	KXH74111.1	Set_4
arCOG01228	SRP	KKK40885.1	KP869631 (AKC94903.1)	KP869592 (AKC94864.1)	KXH77551.1	KXH73387.1	Set_5
arCOG01559	EFG	KKK44407.1	KP869716 (AKC94987.1)	KP869713 (AKC94984.1)	KXH71555.1	KXH72312.1	Set_2
arCOG01560	IF2	KKK43248.1	KP869605 (AKC94877.1)		KXH77376	KXH74767	Set_4
arCOG01722	RPS13	KKK45076.1	KP869650 (AKC94921.1)	KP869617 (AKC94889.1)	KXH70426	KXH75347	Set_6
arCOG01758	RPS10	KKK42106.1	KP869690 (AKC94961.1)	KP869579 (AKC94852.1)	KXH72173	KXH72666	Set_4
arCOG01762	RPB	KKK46191.1	KP869672 (AKC94943.1)	KP869636 (AKC94908.1)	KXH77356	KXH72733.1	Set_4
arCOG04064	Zn protease	KKK42588.1		KP869687 (AKC94958.1)	KXH70900.1		Set_5
arCOG04090	RPL6	KKK42866.1		KP869601 (AKC94873.1)	KXH70445.1		Set_6
arCOG04091	RPS8	KKK42865.1		KP869600 (AKC94872.1)	KXH70446.1		Set_6
arCOG04092	RPL5	KKK41278.1		KP869708 (AKC94979.1)	KXH70447.1		Set_4
arCOG04094	RPL24	KKK41280.1		KP869707 (AKC94978.1)	KXH70449.1	KXH74613.1	Set_4
arCOG04095	RPL14	KKK41281.1	KP869598 (AKC94870.1)	KP869706 (AKC94977.1)	KXH70466.1	KXH74617.1	Set_4
arCOG04096	RPS17	KKK44313.1	KP869597 (AKC94869.1)	KP869705 (AKC94976.1)	KXH70450.1	KXH74612.1	Set_4
arCOG04097	RPS3	KKK44316.1		KP869703 (AKC94974.1)			Set_4
arCOG04098	RPL22	KKK44813.1		KP869702 (AKC94973.1)	KXH70453.1	KXH74609.1	Set_6
arCOG04099	RPS19	KKK44812.1	KP869596 (AKC94868.1)	KP869701 (AKC94972.1)	KXH75360.1	KXH72153.1	Set_6
arCOG04113	RPL10/16	KKK41736.1	KP869619 (AKC94891.1)	KP869591 (AKC94863.1)	KXH77557	KXH73391.1	Set_6
arCOG04121	Rnase H II	KKK43184.1	KP869630 (AKC94902.1)	KP869694 (AKC94965.1)	KXH78084.1	KXH74688.1	Set_4
arCOG04169	SecY	KKK46587.1		KP869723 (AKC94994.1)	KXH70440.1	KXH73725.1	Set_4
arCOG04239	RPS4	KKK45075.1		KP869616 (AKC94888.1)			Set_6
arCOG04240	RPS11	KKK45074.1	KP869659 (AKC94930.1)	KP869615 (AKC94887.1)	KXH70427.1		Set_6
arCOG04241	RPD	KKK46447.1	KP869691 (AKC94962.1)	KP869668 (AKC94939.1)	KXH77381.1	KXH74760.1	Set_4
arCOG04242	RPL13	KKK46445.1	KP869692 (AKC94963.1)	KP869667 (AKC94938.1)	KXH77383.1	KXH74758.1	Set_4
arCOG04243	RPS9	KKK46444.1	KP869693 (AKC94964.1)	KP869666 (AKC94937.1)	KXH77384.1		Set_4
arCOG04245	RPS2	KKK41824.1		KP869618 (AKC94890.1)	KXH77386		Set_4
arCOG04254	RPS7	KKK42224.1	KP869620 (AKC94892.1)	KP869578 (AKC94851.1)	KXH77406.1	KXH74783.1	Set_4
arCOG04255	RPS12	KKK42225.1	KP869626 (AKC94898.1)	KP869609 (AKC94881.1)	KXH77360.1	KXH74782	Set_4
arCOG04256	RPA ‘’	KKK42229.1	KP869652 (AKC94923.1)	KP869608 (AKC94880.1)	KXH77405	KXH74784.1	Set_4
arCOG04257	RPA’	KKK42230.1		KP869608 (AKC94880.1)	KXH77404	KXH74778.1	Set_4
arCOG04289	RPL1	KKK46590.1		KP869606 (AKC94878.1)	KXH77815.1	KXH74331.1	Set_4

For Loki 2 and Loki 3, the CDS accession numbers are indicated, while the protein accession numbers are between brackets.
