# Supplementary material for: Lokiarchaea are close relatives of Euryarchaeota, not bridging the gap between prokaryotes and eukaryotes
Source: PLoS Genet. 2017 Jun 12;13(6):e1006810. doi: 10.1371/journal.pgen.1006810 (PMC5484517; doi:10.1371/journal.pgen.1006810)
Supplement: S5 Table — (PDF) [file pgen.1006810.s046.pdf]

## S5 Table - List of the species sampled for the different phylogenetic analyses in this study.

| | Phylum | Species | Taxon ID | Figure |
|---|---|---|---|---|
| **Eukaryotes** | | | | |
| **Opisthokonta** | insertae sedis | Capsaspora owczarzaki | 595528 | 7 |
| | Metazoa | Homo sapiens | 9606 | 3,7 |
| | | Drosophila melanogaster | 7227 | 7 |
| | | Xenopus (Silurana) tropicalis | 8364 | 7 |
| | | Amphimedon queenslandica | 400682 | 7 |
| | | mus musculus domesticus C57BL/6J | 10092 | 7 |
| | | Aedes aegypti | 1424507 | 3 |
| | Choanoflagellida | Salpingoeca rosetta | 946362 | 7 |
| | | Monosiga brevicollis | 431895 | 7 |
| | Fungi | Aspergillus fumigatus Af293 | 330879 | 7 |
| | | Schizosaccharomyces pombe 972h | 284812 | 7 |
| | | Saccharomyces cerevisae | 765312 | 3,7 |
| | | Batrachochytrium dendrobatidis | 684364 | 7 |
| | | Yarrowia lipolytica | 284591 | 7 |
| | | Ustilago maydis | 237631 | 7 |
| | | Mortierella verticillata | 1069443 | 7 |
| | | Neurospora crassa OR74A | 367110 | 3 |
| | | Verruconis gallopava | 253628 | 3 |
| | | Blastomyces dermatitidis ATCC 26199 | 447095 | 3 |
| **Amoebozoa** | Mycetozoa | Dictyostelium discoideum | 352472 | 3,7 |
| | | Polysphondylum pallidum | 670386 | 7 |
| | | Acytostelium subglobosum | 1410327 | 7 |
| | Archamoebae | Entamoeba histolytica | 5759 | 3 |
| | Discosea | Acanthamoeba castellanii | 1257118 | 7 |
| **Euglenozoa** | Kinetoplastida | Leishmania donovani | 5671 | 3 |
| **Parabasalia** | Trichomonadida | Trichomonas vaginalis | 412133 | 3 |
| **Discoba** | Heterolobosea | Naegleria gruberi | 744533 | 7 |
| **Viridiplantae** | Sreptophyta | Physcomitrella patens | 3218 | 7 |
| | | Oryza sativa | 39946 | 7 |
| | | Arabidopsis thaliana | 3702 | 3,7 |
| | | Selaginella moellendorfii | 88036 | 7 |
| | Chlorophyta | Ostreococcus lucimarinus | 436017 | 3,7 |
| | | Micromonas sp. | 296587 | 7 |
| | Rhodophyta | Galdieria sulphuraria | 130081 | 7 |
| | | Chondrus crispus | 2769 | 7 |
| | Pyrenomonadales | Guillardia theta | 905079 | 7 |
| **SAR** | Stramenopiles | Phytophthora infestans | 403677 | 7 |
| | | Thalassiosira pseudonana | 296543 | 3,7 |
| | | Phaeodactylum tricornutum | 556484 | 7 |
| | | Aureococcus anophagefferens | 44056 | 7 |
| | Alveolata | Oxytricha trifallax | 1172189 | 7 |
| | | Toxoplasma gondii | 508771 | 7 |
| | | Plasmodium falciparum | 36329 | 3,7 |
| | | Plasmodium vivax | 126793 | 7 |
| | | Babesia bigemina | 5866 | 7 |
| | | Hammondia hammondi | 99158 | 7 |
| | | Perkinsus marinus | 423536 | 3 |
| | | Tetrahymena thermophila | 5911 | 3 |
| | Rhizaria | Reticulomyxa filosa | 46433 | 3 |

| | | | |
|---|---|---|---|
| **Bacteria** | | | |
| **PVC Planctomycetes** | Gemmata obscuriglobus UQM 2246 | 214688 | 7 |
| | Rhodopirellula baltica strain SH 1 | 243090 | 7 |
| **Bacterioidetes** | Rhodothermus marinus DSM 4252 | 518766 | 7 |
| | Bacteriodes fragilis | 862962 | 7 |
| | Chlorobaculum parvum NCIB 8327 | 517417 | 7 |
| | Psychroserpens jangbogonensis PAMC27130 | 1484460 | 3 |
| | Flavobacteriales bacterium ALC-1 | 391603 | 3 |
| | Maribacter antarcticus DSM 21422 | 1122191 | 3 |
| | Capnocytophaga gingivalis ATCC 33624 | 553178 | 3 |
| **Gammaproteobacteria** | Escherichia coli str. K-12 substr. MG1655 (W3110) | 511145 | 7 |
| | Legionella longbeachae NSW150 | 661367 | 7 |
| | Acinetobacter baumannii 1656-2 | 696749 | 7 |
| **Firmicutes** | Bacillus subtilis subsp. Subtilis str. 168 | 224308 | 7 |
| | Natranaerobius thermophilus JW/NM-WN-LF | 457570 | 7 |
| | Listeria innocua Clip11262 | 272626 | 7 |
| **Cyanobacteria** | Synechocystis sp. PCC 6714 | 1147 | 7 |
| | Prochloron didemni | 1216 | 7 |
| | Cyanothece sp. PCC 7424 | 65393 | 7 |
| **Deinococcus-thermus** | Deinococcus radiodurans R1 | 243230 | 7 |
| | Truepera radiovictrix DSM 17093 | 649638 | 7 |
| | Marinithermus hydrothermalis DSM 14884 | 869210 | 7 |
| **Thermotogae** | Kosmotoga olearia TBF 19.5.1 | 521045 | 7 |
| | Fervidobacterium nodosum Rt17-B1 | 381764 | 7 |
| | Thermotoga maritima MSB8 | 243274 | 7 |
| **Chloroflexi** | Anaerolinea thermophila UNI-1 | 926569 | 7 |
| | Thermomicrobium roseum DSM 5159 | 309801 | 7 |
| **Actinobacteria** | Catenulispora acidiphila DSM 44928 | 479433 | 7 |
| | Streptosporangium roseum DSM 43021 | 479432 | 7 |
| | Kineococcus radiotolerans SRS30216 | 266940 | 7 |
| **Spirochaetes** | Brachyspira hyodysenteriae WA1 | 565034 | 7 |
| | Treponema azotonutricium ZAS-9 | 545695 | 7 |
| | Borrelia afzelii Pko | 390236 | 7 |
| **PVC Verrucomicrobia** | Coraliomargarita akajimensis DSM 45221 | 583355 | 7 |
| | Opitutus terrae PB90-1 | 452637 | 7 |
| **PVC Chlamydiae** | Simkania negevensis Z | 331113 | 7 |
| | Chlamydia muridarum Nigg | 1434773 | 7 |
| **Deltaproteobacteria** | Pelobacter carbinolicus DSM 2380 | 338963 | 7 |
| | Desulfobulbus propionicus DSM 2032 | 577650 | 7 |
| **Alphaproteobacteria** | Acetobacter pasteurianus IFO 3283-01-42C | 634458 | 7 |
| | Dinoroseobacter shibae DFL 12 | 398580 | 7 |
| | Bartonella bacilliformis KC583 | 360095 | 7 |
| **Betaproteobacteria** | Thiobacillus denitrificans ATCC 25259 | 292415 | 7 |
| | Burkholderia ambifaria AMMD | 339670 | 7 |
| **Parcubacteria** | Parcubacteria bacterium | 1618833 | 3 |

| | | | | |
|---|---|---|---|---|
| **Archaea** | | | | |
| **Crenarchaeota** | Desulfurococcales | Pyrolobus fumarii 1A | 694429 | 7 |
| | | Aeropyrum pernix K1 | 272557 | 7 |
| | | Desulfurococcus kamchatkensis 1221n | 490899 | 7 |
| | | Ignicoccus hospitalis KIN4_I | 453591 | 7 |
| | Sulfolobales | Metallosphaera sedula DSM 5348 | 399549 | 7 |
| | | Sulfolobus tokodaii str.7 | 273063 | 7 |
| | Thermoproteales | Thermoproteus tenax Kra 1 | 768679 | 7 |
| | | Thermofilum pendens Hrk 5 | 368408 | 7 |
| | | Vulcanisaeta moutnovskia 768-28 | 985053 | 7 |
| | | Caldivirga maquilingensis IC-167 | 397948 | 7 |
| | | Pyrobaculum aerophilum str. IM2 | 178306 | 7 |
| **Thaumarchaeota** | | Nitrosopumilus maritimus SCM1 | 436308 | 7 |
| | | Cenarchaeum symbiosum A | 414004 | 7 |
| | | Candidatus Nitrosoarchaeum limnia SFB1 | 886738 | 7 |
| | | Candidatus Nitrososphaera gargensis Ga9.2 | 1237085 | 7 |
| | Aigarchaeota | Candidatus Caldiarchaeum subterraneum ASM27032 | 311458 | 7 |
| | | Candidatus Bathyarchaeota archaeon BA2 | 1700836 | 3,S3 |
| | | Candidatus Bathyarchaeota archaeon B63 | 1779372 | 3 |
| | | Candidatus Bathyarchaeota archaeon B26-1 | 1779370 | 3 |
| | | Candidatus Bathyarchaeota archaeon B26-2 | 1779371 | |
| **Euryarchaeota Cluster I** | Thermococcales | Thermococcus nautili 30-1 | 195522 | 7 |
| | | Thermococcus barophilus MP | 391623 | 7 |
| | | Pyrococcus abyssi GE5 | 272844 | 7 |
| | Methanococcales | Methanotorris igneus Kol 5 | 880724 | 7 |
| | | Methanococcus vannielii SB | 406327 | 7 |
| | | Methanocaldococcus infernus ME | 573063 | 7 |
| | Methanobacteriales | Methanothermus fervidus DSM 2088 | 523846 | 7 |
| | | Methanobrevibacter smithii ATCC 35061 | 420247 | 7 |
| | | Methanothermobacter thermautotrophicus str. Delta H | 187420 | 7 |
| **Euryarchaeota Cluster II** | Archaeoglobales | Ferroglobus placidus DSM 10642 | 589924 | 7 |
| | | Archaeoglobus veneficus | 693661 | 7 |
| | Thermoplasmatales | Ferroplasma acidarmanus fer1 | 333146 | 7 |
| | Methanomassiliicoccales | Candidatus Methanomethylophilus alvus Mx1201 | 1236689 | 7 |
| | DHEV2 | Aciduliprofundum boonei T469 | 439481 | 7 |
| | Methanosarcinales | Methanosarcina mazei Go1 | 192952 | 7 |
| | | Methanococcoides burtonii DSM 6242 | 259564 | 7 |
| | | Methanosaeta harundinacea 6Ac | 1110509 | 7 |
| | Methanomicrobiales | Methanocorpusculum labreanum Z | 410358 | 7 |
| | | Methanoregula boonei 6A8 | 456442 | 7 |
| | Halobacteriales | Natrialba magadii ATCC 43099 | 547559 | 7 |
| | | Haloarcula marismortui ATCC 43049 | 272569 | 7 |
| | Methanocellales | Methanocella paludicola SANAE | 304371 | 7 |
| **Unclassified** | | misc. Crenarchaeota group-6 archaeon AD8-1 | 1685126 | S33 |
| | | misc. Crenarchaeota group archaeon SMTZ1-55 | 1685133 | S33 |
| | | MSBL1 archaeon SCGC AAA261F19 | 1698275 | S33 |
| | | MSBL1 archaeon SCGC AAA259J03 | 1698269 | S33 |
| | | Hadesarchaea archaeon DG-33-1 | 1775755 | S33 |
| | | Hadesarchaea archaeon YNP-N21 | 1776333 | S33 |
| | | Hadesarchaea archaeon YNP-45 | 1776334 | S33 |
| | Asgard group | Lokiarchaeum sp. GC14_75 (archaeon Loki) | 1538547 | 7 |
| | | Candidatus Thorarchaeota archaeon SMTZ1-83 | 1706445 | 8,S33 |
| | | Candidatus Thorarchaeota archaeon SMTZ 1-45 | 1706444 | 8,S33 |
| | | Candidatus Thorarchaeota archaeon AB_25 | 1837170 | 8 |
| | | Candidatus Odinarchaeota archaeon LCB_4 | 1841599 | 8 |
| | | Candidatus Heimdallarchaeota archaeon AB_125 | 1841596 | 8 |
| | | Candidatus Heimdallarchaeota archaeon LC2 | 1841597 | 8 |
