# Supplementary material for: Lokiarchaea are close relatives of Euryarchaeota, not bridging the gap between prokaryotes and eukaryotes
Source: PLoS Genet. 2017 Jun 12;13(6):e1006810. doi: 10.1371/journal.pgen.1006810 (PMC5484517; doi:10.1371/journal.pgen.1006810)
Supplement: S6 Table — (PDF) [file pgen.1006810.s047.pdf]

**S6 Table – Number of ESPs located in the different sets of contigs suggested from the genome quality analysis.**

| Set of contigs | Number of ESP* |
|---|---|
| Set_1 | 1 |
| Set_2 | 33 |
| Set_3 | 2 |
| Set_4 | 48 |
| Set_5 | 32 |
| Set_6 | 56 |

*The 3 ESPs absent from the table among the 175 proposed ESPs (in [14]) are those related to integrated giant viruses proteins.
