# Supplementary material for: Lokiarchaea are close relatives of Euryarchaeota, not bridging the gap between prokaryotes and eukaryotes
Source: PLoS Genet. 2017 Jun 12;13(6):e1006810. doi: 10.1371/journal.pgen.1006810 (PMC5484517; doi:10.1371/journal.pgen.1006810)
Supplement: S30 Fig — The same number (39) of Archaea (green), Eukaryotes (blue) and Bacteria (red) were selected (1,463 positions; see S5 Table for the dataset). Values at nodes indicate the Bayesian posterior probabilities. The scale-bar represents the average number of substitutions per site. (PDF) [file pgen.1006810.s030.pdf]

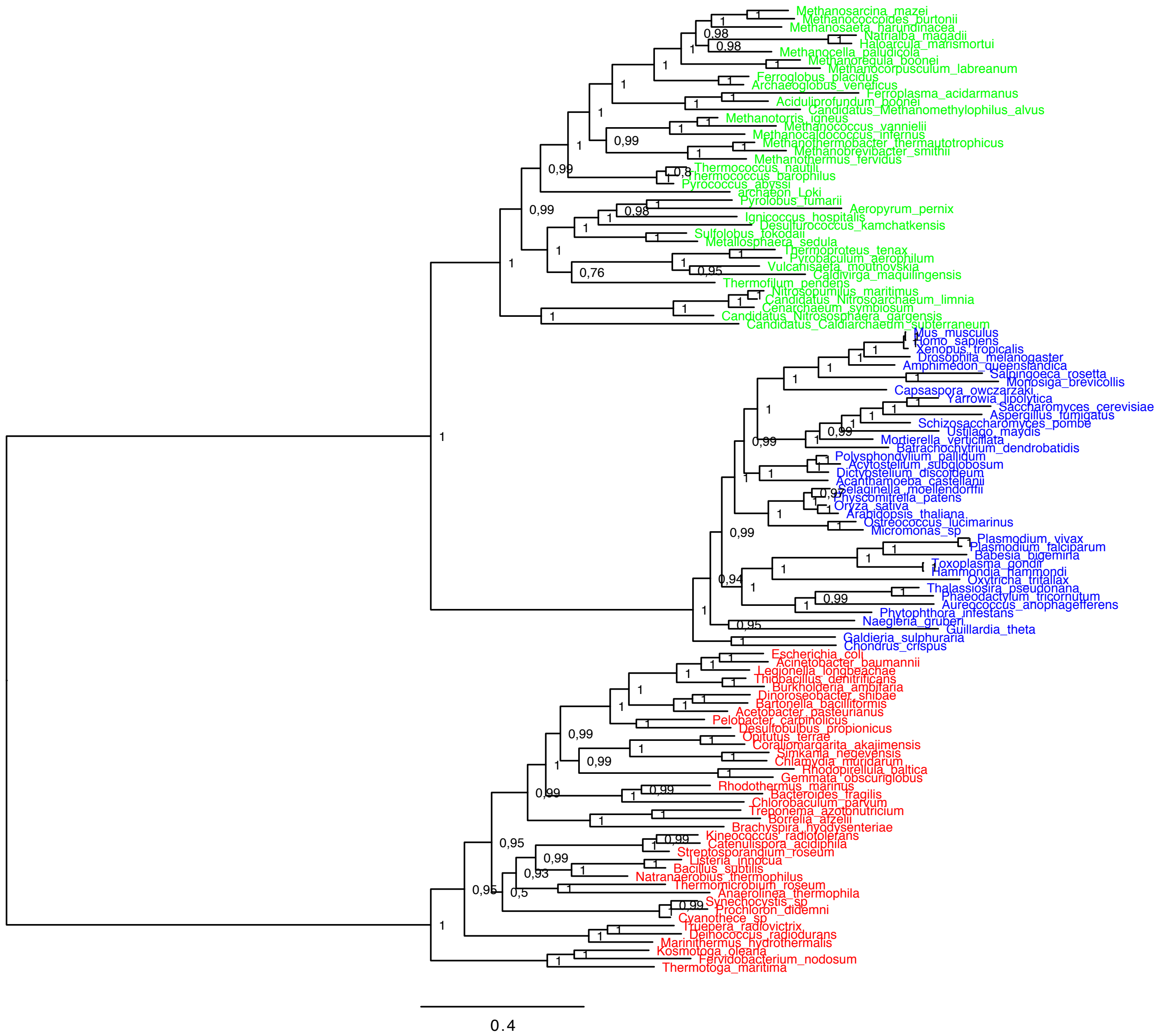

S30 Fig – Bayesian inference phylogeny of the concatenation of the two largest RNA polymerase subunits with LG substitution model (Γ4) on the new dataset.

The same number (39) of Archaea (green), Eukaryotes (blue) and Bacteria (red) were selected (1,463 positions; see S5 Table for the dataset). Values at nodes indicate the Bayesian posterior probabilities. The scale-bar represents the average number of substitutions per site.
