# Supplementary material for: Lokiarchaea are close relatives of Euryarchaeota, not bridging the gap between prokaryotes and eukaryotes
Source: PLoS Genet. 2017 Jun 12;13(6):e1006810. doi: 10.1371/journal.pgen.1006810 (PMC5484517; doi:10.1371/journal.pgen.1006810)
Supplement: S31 Fig — The same number (39) of Archaea (green), Eukaryotes (blue) and Bacteria (red) were selected (1,463 positions; see S5 Table for the dataset). Values at nodes indicate the Bayesian posterior probabilities. The scale-bar represents the average number of substitutions per site. (PDF) [file pgen.1006810.s031.pdf]

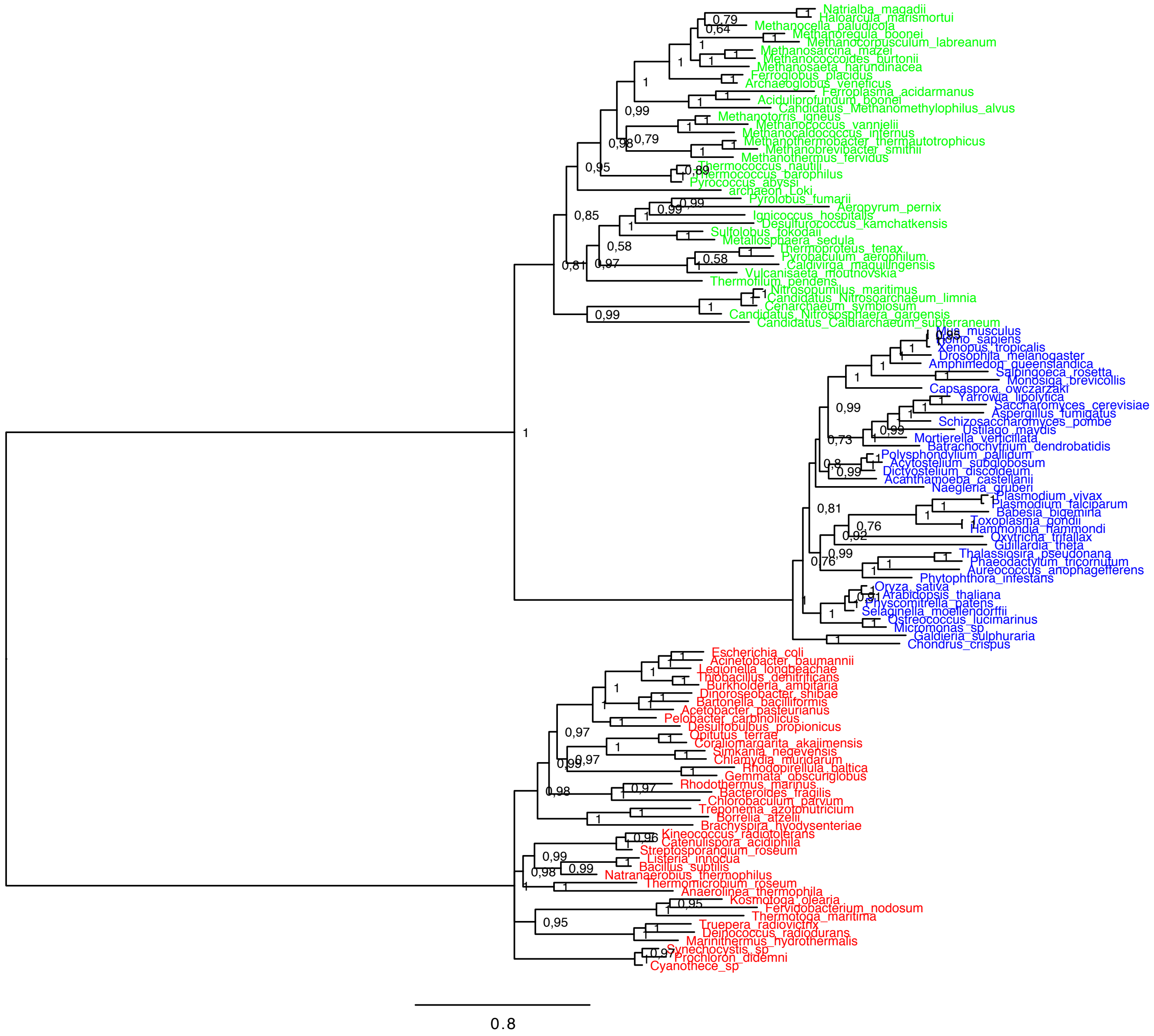

S31 Fig – Bayesian inference phylogeny of the concatenation of the two largest RNA polymerase subunits with CAT-GTR evolution model (Γ4) on the new dataset.

The same number (39) of Archaea (green), Eukaryotes (blue) and Bacteria (red) were selected (1,463 positions; see S5 Table for the dataset). Values at nodes indicate the Bayesian posterior probabilities. The scale-bar represents the average number of substitutions per site.
